# Genome-Wide Analysis Reveals the Unique Stem Cell Identity of Human Amniocytes

**DOI:** 10.1371/journal.pone.0053372

**Published:** 2013-01-10

**Authors:** Colin T. Maguire, Bradley L. Demarest, Jonathon T. Hill, James D. Palmer, Arthur R. Brothman, H. Joseph Yost, Maureen L. Condic

**Affiliations:** 1 Department of Neurobiology and Anatomy, University of Utah, School of Medicine, Salt Lake City, Utah, United States of America; 2 Department of Pediatrics, University of Utah, School of Medicine, Salt Lake City, Utah, United States of America; 3 Department of Human Genetics, University of Utah, School of Medicine, Salt Lake City, Utah, United States of America; 4 Department of Pathology, University of Utah, School of Medicine, Salt Lake City, Utah, United States of America; 5 University of Utah Molecular Medicine Program, University of Utah, School of Medicine, Salt Lake City, Utah, United States of America; 6 ARUP Institute for Clinical and Experimental Pathology, ARUP Laboratories, Salt Lake City, University of Utah, School of Medicine, Salt Lake City, Utah, United States of America; University of Houston, United States of America

## Abstract

Human amniotic fluid contains cells that potentially have important stem cell characteristics, yet the programs controlling their developmental potency are unclear. Here, we provide evidence that amniocytes derived from multiple patients are marked by heterogeneity and variability in expression levels of pluripotency markers. Clonal analysis from multiple patients indicates that amniocytes have large pools of self-renewing cells that have an inherent property to give rise to a distinct amniocyte phenotype with a heterogeneity of pluripotent markers. Significant to their therapeutic potential, genome-wide profiles are distinct at different gestational ages and times in culture, but do not differ between genders. Based on hierarchical clustering and differential expression analyses of the entire transcriptome, amniocytes express canonical regulators associated with pluripotency and stem cell repression. Their profiles are distinct from human embryonic stem cells (ESCs), induced-pluripotent stem cells (iPSCs), and newborn foreskin fibroblasts. Amniocytes have a complex molecular signature, coexpressing trophoblastic, ectodermal, mesodermal, and endodermal cell-type-specific regulators. In contrast to the current view of the ground state of stem cells, ESCs and iPSCs also express high levels of a wide range of cell-type-specific regulators. The coexpression of multilineage differentiation markers combined with the strong expression of a subset of ES cell repressors in amniocytes suggests that these cells have a distinct phenotype that is unlike any other known cell-type or lineage.

## Introduction

Amniocytes are a fascinating fetal cell-type whose precise developmental role remains unclear. Recent findings have sparked a surge of excitement among scientists looking for patient-derived sources of therapeutic stem cells, yet current knowledge is compromised by the small number of patient samples studied and the limited analyses performed. Consequently, the literature is incomplete and at times contradictory. The difficulty of driving amniocytes directly into specific lineages hampers the ultimate goal of transplanting and functionally engrafting them into diverse tissues in order to treat specific congenital defects in utero or in children [1]–[4]. While amniocytes may hold promising therapeutic potential [5]–[10], the molecular mechanisms controlling their developmental status are not understood, and a comprehensive characterization of these cells is clearly required before patient-derived amniocyte stem cell therapy becomes a clinical reality.

Human amniocytes are considered an embryonic or fetal multipotent stem cell due to expression of transcriptional regulators [11]–[14] and cell surface antigens [15]–[18] characteristic of stem cells. Interestingly, amniocytes can be efficiently reprogrammed into a primitive pluripotent state by DNA-integrating [19]–[25] and non-integrating methods [18], and subsequently differentiated along multiple lineages [17], [18], [22], [26]–[32]. Alternatively, they can be reprogrammed through direct methods, which are thought to bypass pluripotency altogether [33], or as our data suggests, use some of the innate pluripotency of amniocytes. Like human embryonic stem cells (hESCs), amniocytes are highly proliferative, but unlike ESCs, they do not produce tumors *in vivo* and are not immortal [17]. Despite these important findings, the regulatory networks controlling the developmental status of amniocytes are still undefined.

To better define the developmental status of amniocytes, we examined samples from a large number of patients by immunostaining, flow cytometry, clonal analysis, qPCR and RNA-seq whole-genome profiling. Our bioinformatic analyses of amniocyte, hESC and hIPSC transcriptomes reveal clear distinctions among these populations. Relevant to clinical applications, we asked whether amniotic stem cell dynamics are dependent on gestation, gender, or time in culture. Strikingly, amniocyte profiles resemble transitioning cell-types that co-express markers for both undifferentiated and differentiated derivatives. Clonal analysis indicates that amniocytes are capable of self-renewal and generating multiple distinct pluripotent lineages. Together, our findings suggest molecular mechanisms maintain amniocytes in a stem cell state while simultaneously activating and repressing diverse sets of signaling and differentiation programs.

## Results

### Amniocytes Uniformly Express Pluripotency Transcription Factors, but Cell Surface Pluripotency Antigens Are Heterogeneous

**Figure 1:**
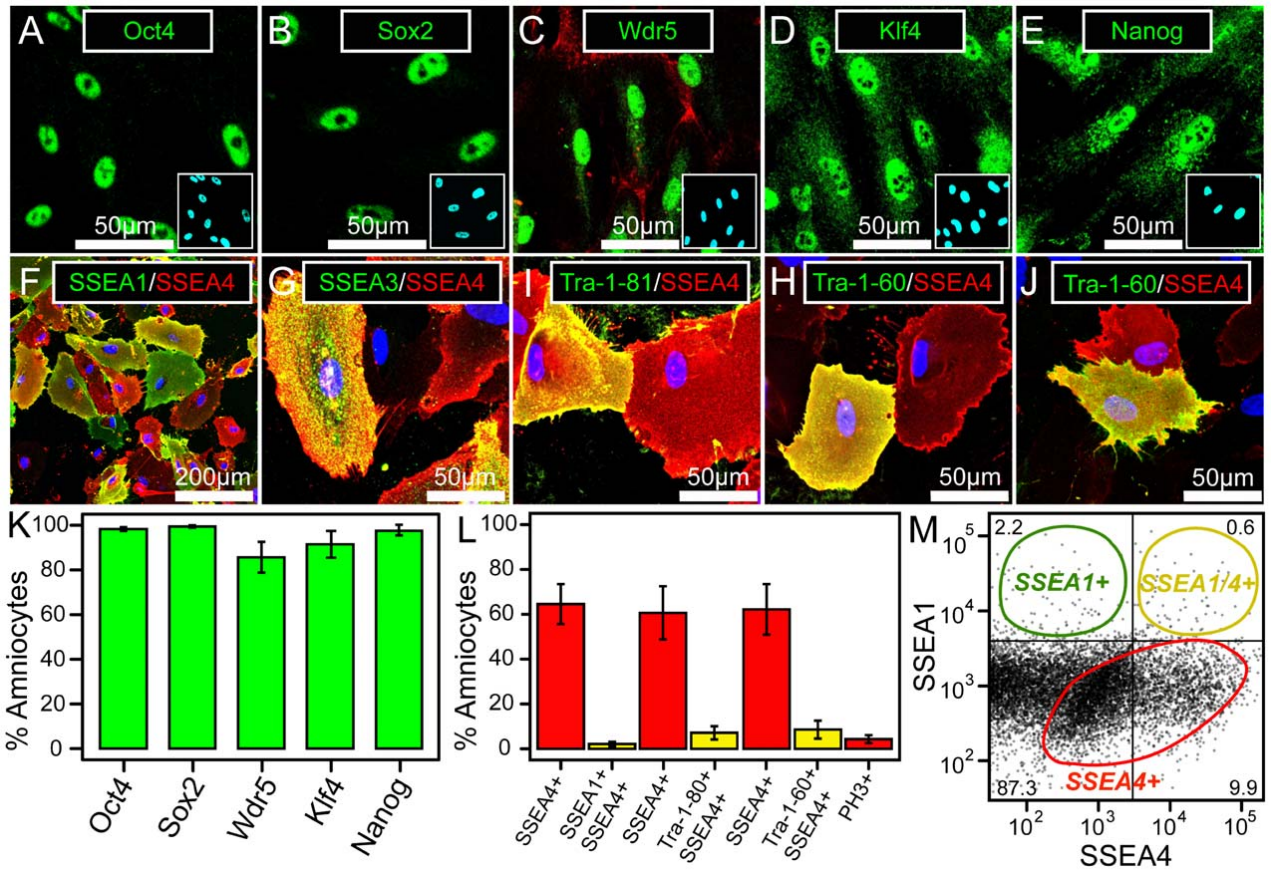
Amniocytes have properties of pluripotent stem cells. (A–E) Confocal images of amniocytes immunostained (green) for transcription factors as indicated. Hoechst dye was used to label nuclei (cyan-colored insets) in all panels and cells in panel C were stained with α-actinin to visualize the lateral cell border and cytoskeletal remodeling (red in panel C). 6,143 cells were counted for all conditions. (F–J) Confocal images of amniocytes co-stained for cell surface antigens as indicated. (H) SSEA4 and Tra-1-60 staining in an (H) undifferentiated population and (J) staining from clonal analysis reveals that individual amniocyte clones give rise to a heterogeneous population of progeny that had similar properties to the parent population. (H–J) Each of these panels show two cells, both expressing SSEA4 but only one coexpressing Tra-1-60. (K) Amniocyte isolates that are positive for transcriptional markers associated with pluripotency express these markers in >90% of nuclei. 19,010 cells were counted for all conditions. (L) The average percent amniocytes per isolate co-expressing surface stem cell markers, ± standard error of the mean. More than 60% of amniocytes stained positive for SSEA4, whereas far fewer cells co-stained for SSEA1 (2.1%, N = 11 isolates), Tra-1-60 (8.5%, N = 7 isolates), and Tra-1-81 (7.1%, N = 7 isolates). Amniocytes exhibit a high rate of proliferation (4.3%), as counted by anti-phospho-histoneH3 (PH3; N = 7 isolates). (M) FACS analysis of SSEA1/SSEA4 amniocytes reveals three distinct populations: low-to-high expressing SSEA4-positive (red circle); high-expressing SSEA1-positive (green circle); and high-expressing double-stained SSEA1+/SSEA4-positive (yellow circle). Percent of cells are indicated in each quadrant.

Previous reports have indicated that cultured amniocytes exhibit many properties of multipotent [2], [17], [27], [34] and pluripotent [18] stem cells. However, it is unclear whether amniocyte subpopulations occupy distinct pluripotent states. We therefore examined the distribution of core transcription factors known to regulate pluripotency by immunofluorescent staining (Figure 1A–E).

Amniocytes expressed cytoplasmic and nuclear Oct4 (Pou5f1), Sox2, Nanog, and Klf4. Low levels of cKit (*Kit*) were detected, consistent with previous reports [17], [30]. We also detected nuclear expression of Wdr5 (Figure 1C), a key member of the mammalian Trithorax complex that interacts with Oct4 [35]. When an amniocyte isolate was positive for these pluripotency factors, expression was consistent, with over 90% of cells showing nuclear localization (Figure 1A–E; Figure 1K). However, expression was highly variable across isolates, with many samples being negative for one or more of these factors: Oct4 (present in 19/34 isolates tested), Sox2 (2/25), Wdr5 (11/13), Klf4 (8/10), or Nanog (9/15). Taken together, our results indicate most amniocytes show nuclear localization of multiple transcription factors associated with pluripotency, but expression levels of these factors are highly variable among samples derived from different patients.

**Figure 2:**
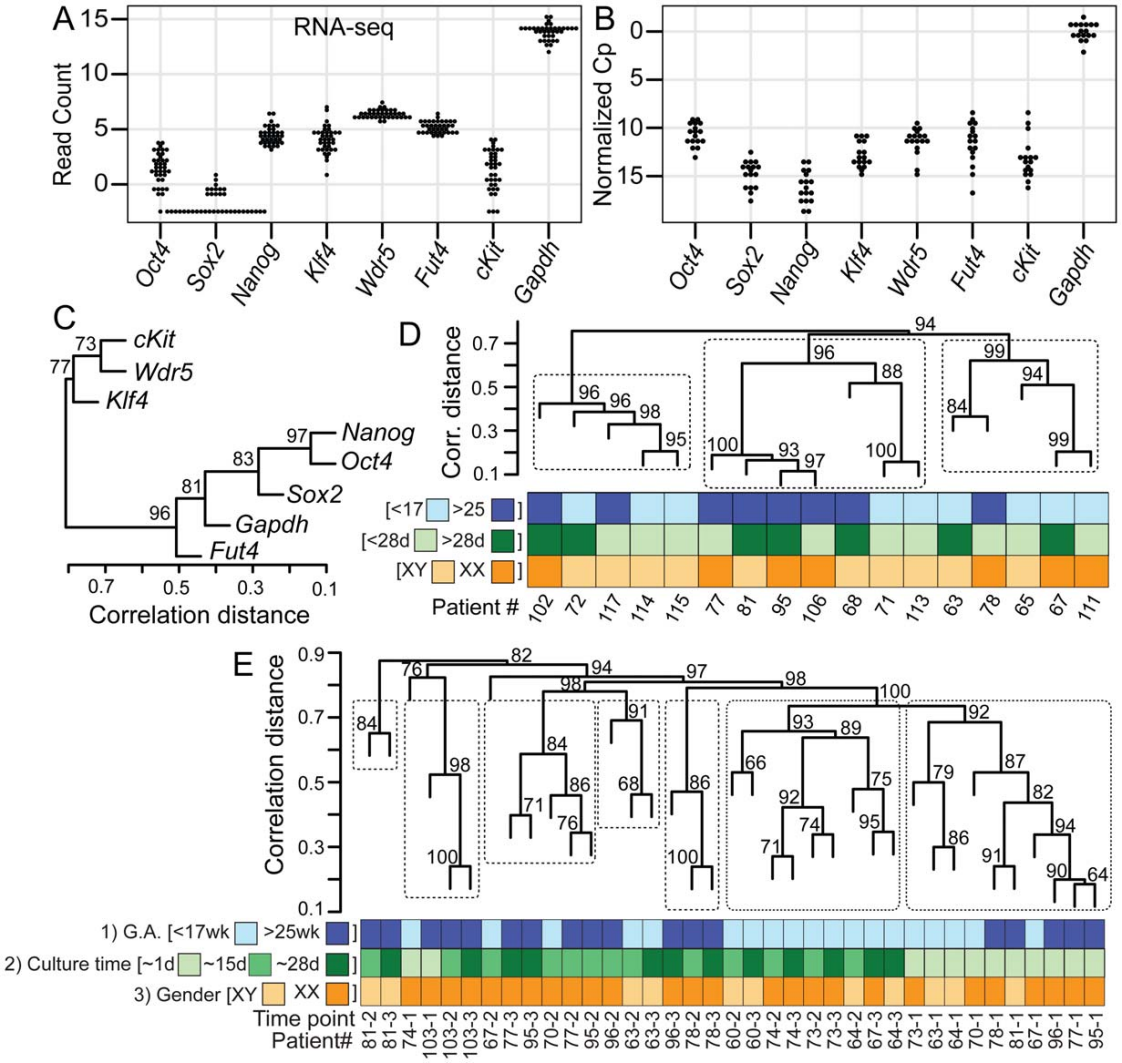
Core stem cell markers are variably expressed, depending on GA and time in culture. (A–B) Dot plots of (A) RNA-seq and (B) qPCR results reveal significant variability in transcript levels for key genes known to be required for establishment and maintenance of pluripotency. (A) RNA-seq measurements for 37 datasets are presented as variance-stabilized read counts. The string of horizontal dots at the lower detection limit for genes Oct4, Sox2 and cKit indicates samples that had no reads in those genes. (B) qPCR units for 17 datasets are presented as normalized Cp values (Cp value of target gene minus Cp value of reference gene Gapdh). (C–E) Hierarchical clustering of C) qPCR results for eight genes; (D) qPCR results for 17 patients; and (E) RNA-seq results for 37 datasets using measurements of 250 stem cell markers. Clustering similarities in transcript levels were calculated by Pearson's r^2^ correlation coefficient as a measure of dendrogramatic distance and bootstrapping values were calculated from 10,000 random replications. (E) Culture time point T1 was taken on average from 1.3 days (0–8 range), T2 was taken on average from 15.2 days (13–22 range), and T3 was taken on average from 28.0 days (24–36 range).

The cell surface antigens SSEA1, SSEA3, SSEA4, Tra-1-60, and Tra-1-81 are important markers for pluripotency. During ES cell differentiation, disappearance kinetics for each marker differ. Therefore, expression of these factors cannot be taken as an absolute indicator of pluripotency [36], and overall patterns of expression must be considered. Subsets of amniocytes express all five of these markers (Figure 1F–H), strongly suggesting the population is a heterogeneous mixture containing some ESC-like cells. Consistent with SSEA-1 immunoreactivity, *Fut4* mRNA transcripts were detected in amniocytes by RNA-seq and by qPCR (Figure 2A–B). The *Fut4* gene encodes a fucosyltransferase that forms SSEA1-containing (also known as Lewis X and CD15) glycoconjugate chains [37], .

FACS analysis of the surface antigens SSEA1 and SSEA4 revealed three distinct subpopulations: a large group of low-to-high expressing SSEA4+ cells, and two smaller populations containing high-expressing SSEA1 or double positive high-expressing SSEA1+/SSEA4+ (Figure 1M). To confirm this observation, we double-stained amniocytes for combinations of cell surface markers for pluripotency. The expression pattern of SSEA1, SSEA3, SSEA4, Tra-1-60, and Tra-1-81 was strikingly heterogeneous (Figure 1F–H). On average, 60% of amniocytes were SSEA4+ (Figure 1L), albeit the prevalence of this marker varied from 8% to 96% among different amniocyte isolates. Interestingly, subpopulations of SSEA1, SSEA3, Tra-1-60, and Tra-1-81 almost always co-stained positive for SSEA4 (over 90%). Tra-1-60 and Tra-1-81 subpopulations appear in parallel and are likely to overlap, but we could not verify this because both of these antibodies are only available as the same isotype, preventing costaining. Thus, amniocytes contain a large subpopulation of SSEA4+ cells and smaller populations of SSEA1+/SSEA4+, Tra-1-60+/SSEA4+, and Tra-1-81+/SSEA4+ cells.

To determine whether amniocytes are capable of self-renewal of their undifferentiated character when expanded, we performed clonal analysis. Singly dissociated amniocytes from seven patient isolates were plated into 96-well plates and wells containing a single cell identified. After one week, a subset of cells in all of the clones expressed SSEA4, with some clones also containing Tra-1-60+ cells. Cell counting of these co-stained clones revealed that 30% of cells were positive for SSEA4 alone and 0.5% were double-positive for Tra-1-60 and SSEA4 (n = 3129 cells from 5 independent clones). A majority of Tra-1-60-positive cells (81%) were double-positive for SSEA4. Differences in absolute percentages between clones and the parental population may reflect differences in time in culture (7 days versus 19 days) or differences between patients, yet the heterogeneous state of the original population is clearly recovered after cloning (Figure 1J). If amniocytes were not able to self-renew the distinct subpopulations seen with multiple markers (Fig. 1 F–I, L), individual clones would produce a relatively homogenous population of progeny, with only a single subset of markers. Moreover, we did not observe decreased expression of stem cell markers over time in culture within the parent populations (see Figure 2), suggesting the heterogeneity we see in clonal populations is not likely to reflect loss of these markers due to cell differentiation. The simplest interpretation of these results is that amniocytes contain self-renewing cells that have a distinct amniocyte phenotype.

### Individual Patient Isolates Exhibit Significant Variability in Expression of Stem Cell Markers

To precisely measure transcript levels of stem cell markers across individual patients, we analyzed 37 RNA-seq datasets, representing 11 isolates from patients at different gestational ages and different times in culture. RNA-seq results revealed significant variability across our patient pool in expression of stem cell markers (Figure 2A). We confirmed that inter-patient variability is present among amniocyte samples by performing qPCR on RNA from 17 independent amniocyte isolates (17 patients) (Figure 2B). Gene expression was highly variable for the seven stem cell markers. To assess whether there might be any similarities in transcript levels for these seven marker genes, we analyzed the qPCR dataset by hierarchical clustering (Figure 2C). *Oct4*, *Nanog*, *and Sox2* clustered closest in a node together. However, when we performed a comparable analysis with the RNA-seq dataset, the branches of the dendrogram shifted significantly, likely influenced by non-detection of *Sox2* in 24/37 samples. The discordance between the two datasets may be due to differences in the ability of RNA-seq and qPCR to detect transcripts expressed at low abundance, with qPCR being less quantitative, but more sensitive. Additionally, it is important to note that different genes have different amplification efficiencies, and comparing numbers of transcripts across multiple genes or patients without proper compensation for those differences can be problematic. Both qPCR and RNA-seq methods have inherent technical disadvantages that should be carefully considered so that the data is not over-interpreted. Despite these limitations, results from qPCR and RNA-seq can lend concurrence and support each other. Nonetheless, these results indicate that every amniotic isolate expresses distinct levels of stem cell markers.

### Expression of Stem Cell Markers Is Dynamic in Amniocytes and Changes with Gestational Age and Culture Time

Normal amniotic fluid is complex and dynamic [39]. Due to the broad range of Cp values observed for stem cell markers (Figure 2B), we asked whether gestational age, culture time, or gender could account for those differences. Despite significant variation, the median transcript levels for early and late gestational ages were essentially the same for stem cell markers examined by qPCR. Hierarchical clustering analysis of qPCR data showed three main cluster groups having little correlation between gestational age, time in culture, or gender (Figure 2D).

Examining our panel of seven stem cell markers by RNA-seq, only *Nanog* transcript levels (adjusted *p*-value = 0.04) were higher in older gestational isolates than younger gestational isolates, whereas *Oct4, Sox2, Klf4, Wdr5, Fut4*, and *cKit* were unchanged (Table S1). To further examine whether gestational changes exist in amniocytes isolates, we expanded our list of stem cell markers from seven to 250 genes that have been implicated in playing functional roles in stem cell maintenance (see Table S2 for supporting references). Hierarchical clustering of 37 RNA-seq datasets for the 250 stem cell markers (Figure 2E) revealed that similar gestational ages showed stronger clustering than the qPCR data (Figure 2C). Taken together, these results suggest that some aspects of amniocyte stem cell state are dynamically regulated during gestation, but not the entire stem cell signature.

We next asked whether the expression profile of shorter cultured isolates (less than four weeks) also differed from longer cultured isolates (over four weeks). Hierarchical clustering of qPCR data from 17 independent patients showed weak grouping among the three main nodes for time in culture (Figure 2C). However, when we analyzed our panel of 250 stem cell markers in our RNA-seq datasets, strong clustering occurred among shorter cultured isolates (Culture time: T1) and separately, among longer cultured (Culture time: T2 and T3) isolates (Figure 2E). These results emphasize the important influence of length of culture time on stem cell transcript levels in cultured amniocytes, which has important implications in clinical applications.

### The Amniocyte Transcriptome Segregates by Gestational Age, Culture Time, and Gender

**Figure 3:**
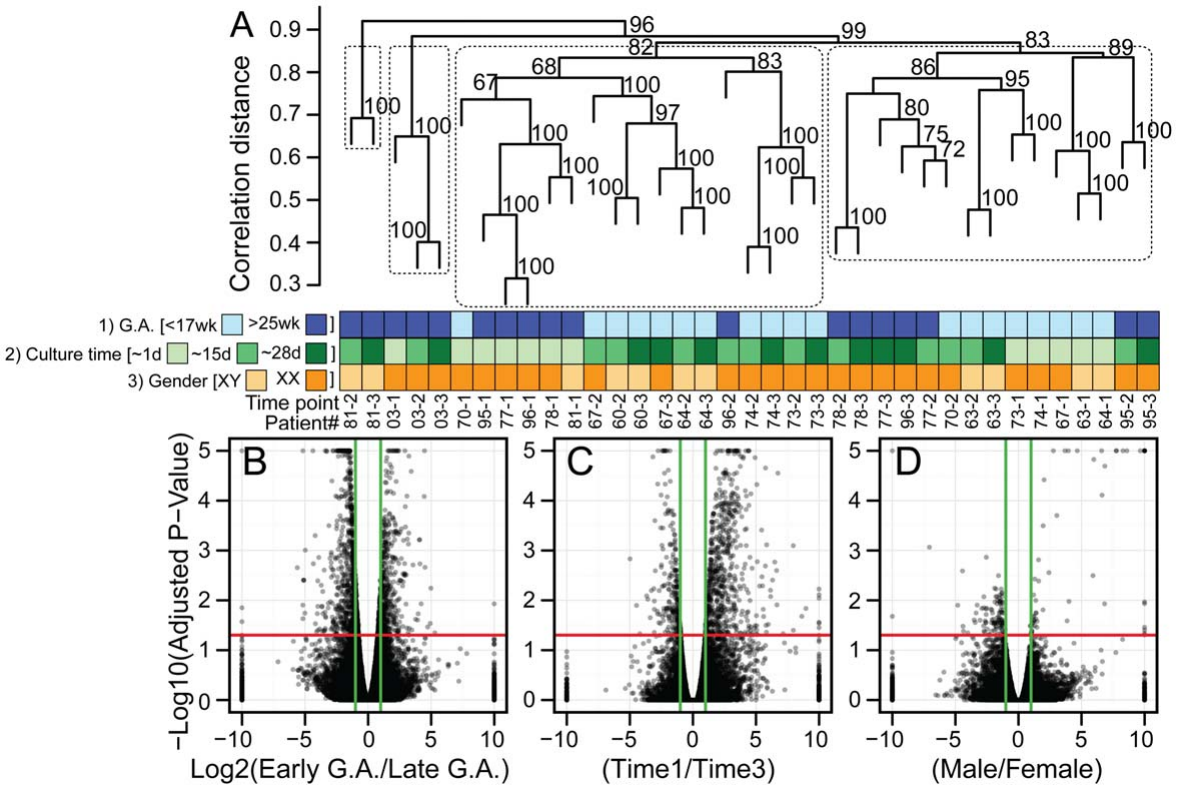
Amniocyte genome-wide transcriptional profile varies depending on GA and time in culture. (A) Hierarchical clustering of 11 independent amniocyte isolates at different times in culture (37 RNA-seq datasets; 24,609 ensemble genes per dataset). Four major clusters correlate with gestational age and culture time since amniocentesis. (B–D) Volcano plots display the results of differential expression analyses using these three variables (n = 49,235 Ensembl genes). Genes plotted above the red line have adjusted *p*-values<0.05, and genes plotted outside of the green lines are >2-fold differentially expressed. Comparing (B) early vs. late gestational age revealed the most differentially expressed genes (n = 2,197), followed by (C) time in culture (n = 1039) and (D) gender (n = 208). (A, C) Culture time point T1 was taken on average from 1.3 days (0–8 range), T2 was taken on average from 15.2 days (13–22 range), and T3 was taken on average from 28.0 days (24–36 range).

In agreement with our stem cell marker findings (Figure 2), on a genome-wide scale (total 49235 ensemble coding genes and non-coding products), individual patients segregated by gestational age, culture time, and (to a lesser extent) gender (Figure 3A). To determine which variables were driving clustering, we performed differential expression analyses. Volcano plots [40] identified significant numbers of differentially enriched genes (Figure 3B, 3C, 3D). Comparison of early to late gestation samples revealed the largest number of differentially expressed genes (n = 2,197 genes with p-adjusted<0.05), followed by early to late times in culture (n = 1039 genes with p-adjusted<0.05). Comparison of male to female samples revealed the fewest number of differentially expressed genes (n = 208 genes with p-adjusted<0.05), most of which were X- or Y-linked genes. These data indicate that gene expression is dependent on gestational age, culture time, and to a lesser extent gender.

### Amniocyte Isolates Have a Distinct Expression Signature with Limited Similarities to ESC and iPSC Lines

Although amniocyte isolates are enriched for well-accepted pluripotent markers (Figure 1 and 2A–B), whether they reside closer to a multipotent state or to a more primitive pluripotent state has not been clearly distinguished. To elucidate whether amniocytes share any resemblance to a primed (epiblastic) pluripotent state, we used several bioinformatic approaches to compare our full amniocyte transcriptome RNA-seq datasets to publicly available datasets from ESC, iPSC, and human newborn foreskin fibroblasts (NFF: the parental line for the reference iPSC line). Hierarchical clustering of RNA-seq datasets generated in different laboratories clustered together (Figure S1), suggesting that technical variation might be a significant variable. Despite this limitation, variation in gene expression between primed pluripotent stem cells (ESC and iPSC lines), amniocytes and NFFs segregates these cell-types into distinct groups.

**Figure 4:**
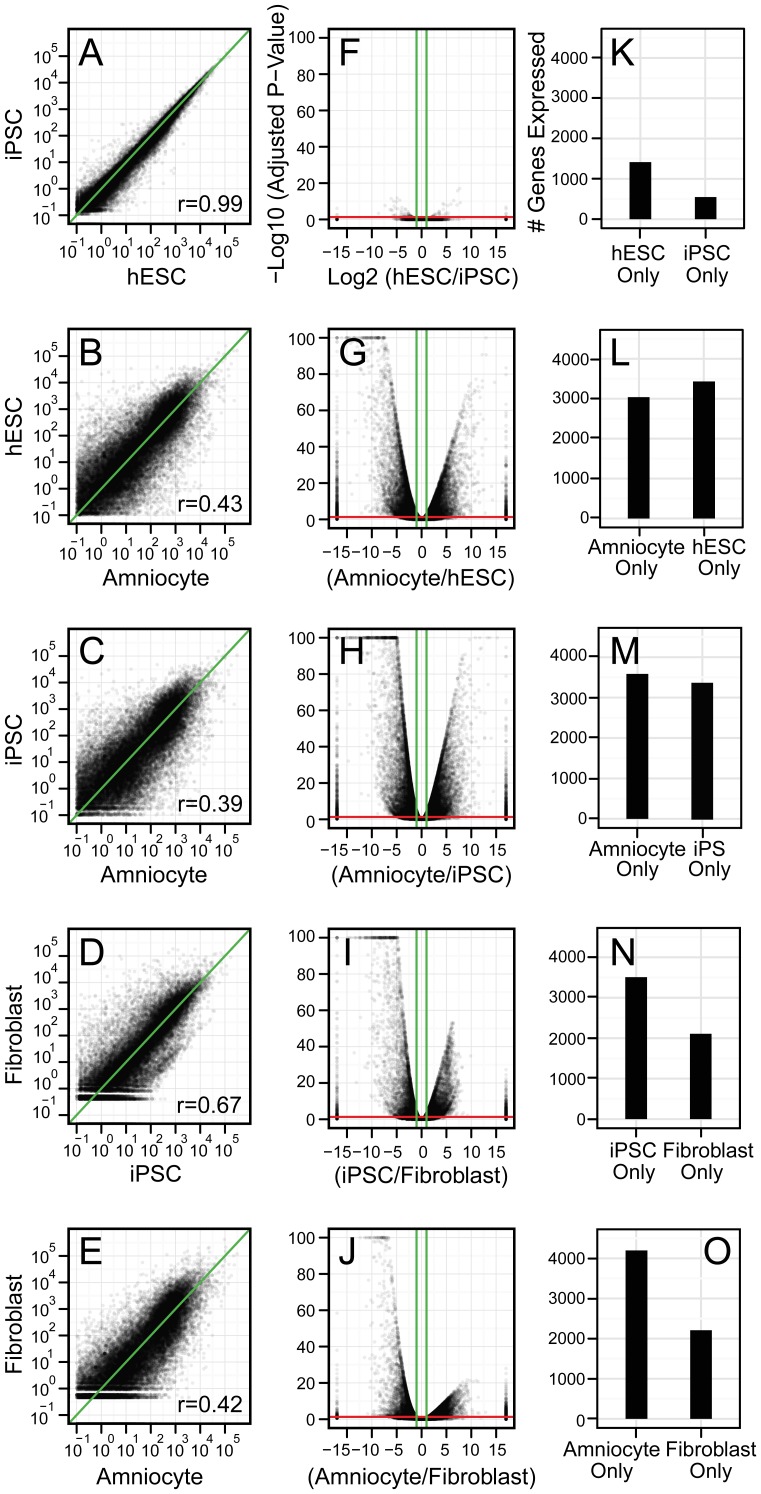
Amniocytes are not transcriptionally similar to ESCs and iPSCs. (A) Genome-wide scatterplots (49,235 Ensembl genes) confirm that ESC and iPSC cells lines are remarkably similar in gene expression profiles. (B–C) In contrast, genome-wide scatterplots comparing amniocytes to (B) ESC lines and (C) iPSC lines show weak similarity. (D–E) The transcriptomes of (D) iPSC cell lines and human newborn foreskin fibroblasts (NFF) are also weakly similar, (E) as are amniocytes and NFF. (F–J) Volcano plots (as in Figure 3) display the results of differential expression analyses. (K–O) Bar graphs show the number of genes that were expressed exclusively in either cell type.

To better understand the transcription signature of amniocytes, we used a genome-wide comparison. Confirming results previously published by Phanstiel et al [41], the transcriptomes of ESC and iPSC lines show remarkable similarity (Figure 4A). In contrast, the transcriptome of amniocytes showed only limited similarity to ESC or to iPSC lines (Figure 4B and 4C). Comparing iPSC lines with their parental NFFs demonstrates how dramatically reprogramming alters the epigenetic signature in iPSC lines (Figure 4D). The transcriptional profile of amniocytes was as equally dissimilar with NFFs (Figure 4E) as ESC and iPSC lines (Figure B–C).

To identify which transcripts were specifically increased or decreased, we reanalyzed our datasets as volcano plots (Figure 4F–J). Very few genes differed between ESC and iPSC lines (Figure 4F). Previous work [42] identified *Tcerg1l* and *Fam19a5* as transcripts that were consistently higher in ESC lines, whereas *Tbx15* and *Pitx2* were consistently higher in iPSC lines. Interestingly, relative transcript levels for all of these genes in amniocytes were closer to iPSC than ESC lines (Table S3). In contrast, thousands of genes were either 2 fold higher or lower in amniocyte versus ESC (Figure 4G), amniocyte versus iPSC (Figure 4H), or amniocyte versus NFF (Figure 4J) comparisons. Similar to amniocyte versus NFF, a large number of transcripts were differentially regulated between iPSC and NFF lines (Figure 4I).

In total, we found 20,512 (adjusted *p*-value<0.05) differentially regulated transcripts between ESCs and amniocytes, 22,443 transcripts between iPSCs and amniocytes, and 16,264 transcripts between NFFs and amniocytes. The ten transcripts most enriched in ESC and iPSC lines (compared to amniocytes) contain a large number of well-characterized stem cell markers (Table S3). In contrast, the top ten genes enriched in amniocytes are generally involved in more differentiated states. These results indicate that the expression signature of ESC and iPSC lines is highly similar, but distinct from that of amniocytes.

### The Stem Cell State of Amniocytes is Unique and Distinct from ESC and iPSC Lines

**Figure 5:**
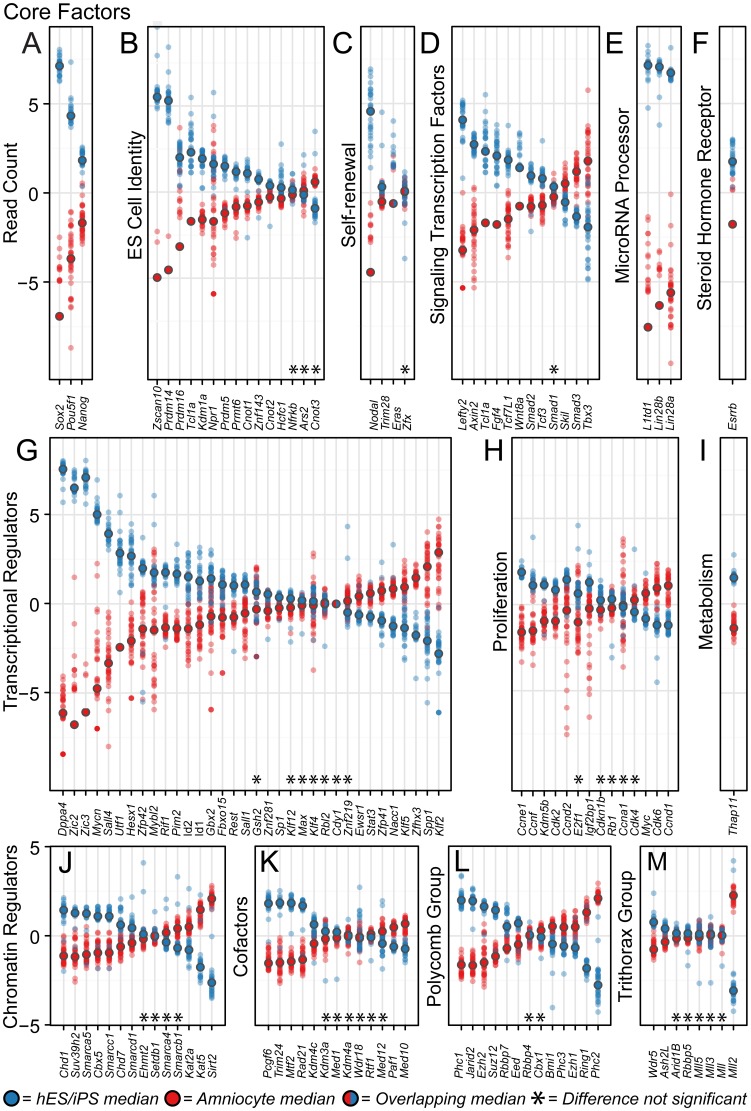
Amniocytes have a distinct transcriptional profile for key pluripotency genes. (A–M) Differential expression analysis of RNA-seq datasets reveals the transcriptional profile controlling the stem cell state in amniocytes conspicuously differs from well-characterized ESC or iPSC lines. The selected 135 genes (Table S2) were grouped into transcriptional regulatory circuits based on their major functional role. Dot plots show RNA-seq read counts (variance corrected) that were median centered for 37 amniocyte (red dots) isolates and 31 ESC and iPSC replicates (blue dots). Circles outlined in black indicate the median value for each group. Within each panel, genes were sorted raw RNA-seq counts first by variance correction, second by the difference between the group medians [median (stem cell samples) – median (amniocyte samples)] and third by ordering the values from highest difference to lowest. All panels use the same y-axis scale, but unused portions of panels K-M were cropped (over +5 and under −5 read counts) because these areas were blank. Asterisks indicate genes with read counts that are not statistically different between amniocytes and ESC/iPSC (defined as the adjusted *p*-value is greater than 0.05).

Based on their expression of key pluripotency factors (Figures 1–2), amniocytes occupy an embryonic stem cell-like state, but this state is clearly distinct from true primitive pluripotency (Figure 4). To more precisely define the stem cell state in amniocytes, we selectively analyzed 135 regulatory genes (Figure 5) required for the pluripotent state (see Table S2 for references).

We first asked whether the three core factors, *Sox2*, *Oct4* and *Nanog*, have comparable transcript levels in amniocytes and ESC/iPSC lines. Interestingly, *Sox2* (adjusted *p*-value = 3.19×10^−219^), *Oct4* (7.56×10^−29^), and *Nanog* (7.48×10^−32^) were expressed at many fold lower levels in amniocytes than in ESC and iPSC lines (Figure 5A). In fact, *Sox2* transcripts were undetectable in a majority of amniocyte samples (23/36) and barely detectable in the other 13 samples. Despite the low transcript levels of Sox2, most amniocyte samples express Oct4 and Nanog at detectable levels, but overall these key pluripotent genes, which sit hierarchically at the top of the ESC core regulatory circuit, are expressed at much lower levels than observed in true pluripotent cells.

Similar to the core factors that regulate ESC pluripotency, the majority of transcription factors, cofactors, chromatin regulators and microRNA processors known to contribute to the ESC state [43] are expressed at significantly higher levels in ESC and iPSC lines (Figure 5B–M), but some are expressed higher in amniocytes (27/135). In total, the ordering of differentially enriched genes revealed that only 34 of 135 genes have similar transcript levels between ESCs and amniocytes. These results indicate that the core factors controlling embryonic stem cell identity are remarkably dissimilar between amniocytes and well-characterized pluripotent cells. Furthermore, marked differences in transcript levels for a wide range of important transcriptional, chromatin and micro-RNA regulators suggest that the downstream molecular mechanisms maintaining stem cell state and differentiation potential are distinct in amniocytes.

### The Stem Cell State of Amniocytes Has Unique Repressors

**Figure 6:**
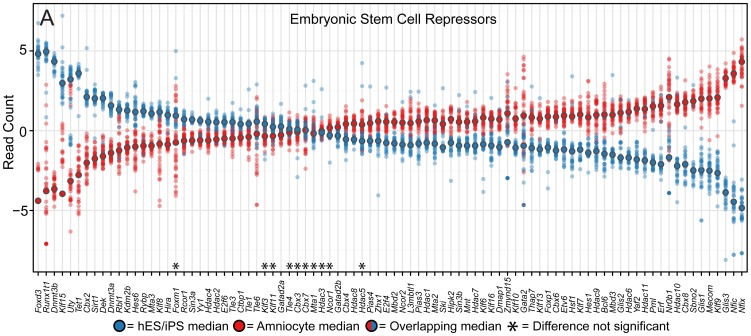
Amniocytes exist in a unique state of transcriptional repression compared to ESC and iPSC. (A) Differential expression analysis of RNA-seq datasets reveals that the repressed state in amniocytes is distinct from ESC/iPSC. Dot plots show RNA-seq read counts analyzed as in Figure 5. Eighty-nine important ES cell repressors in mouse and human ES cells (Table S4) were sorted as in Figure 5. Asterisks = no statistical difference between amniocytes and ESC/iPSC (defined as the adjusted *p*-value is greater than 0.05).

Transcriptional repression in ES cells is critically important for maintaining pluripotency and self-renewal. Since amniocytes have a unique stem cell-like identity, we asked whether the transcript levels of known ES cell repressors are similar among amniocytes, ESC, and iPSC lines. Using differential expression analyses, we selectively analyzed 89 ES cell repressors (Figure 6; Table S4). While most of these repressors were expressed at high levels (72/89 genes had raw read counts >100), or even at extremely high levels (26/89 genes had raw read counts >1000), only 10 of 89 genes have similar transcript levels between amniocytes and ESC and iPSC lines. Intriguingly, *Nr0b1* (*Dax1*) is highly enriched in amniocytes and has previously been reported to be a potent inhibitor of Oct4 transcriptional activity [44]. Overall, the transcript levels for this selected set of transcriptional repressors in amniocytes do not closely match those in ESC and iPSC lines. This finding implies gene-specific repression of amniocyte transcription is likely distinct and gene activity of signaling pathways regulating their developmental potency is likewise distinct. Based the transcriptional signature of ES cell repressors in amniocytes, we conclude although they have many characteristic features of pluripotent stem cell, they exist in a uniquely repressed state.

### Amniocytes Coexpress Lineage-specific Markers for All Three Germ Layers

**Figure 7:**
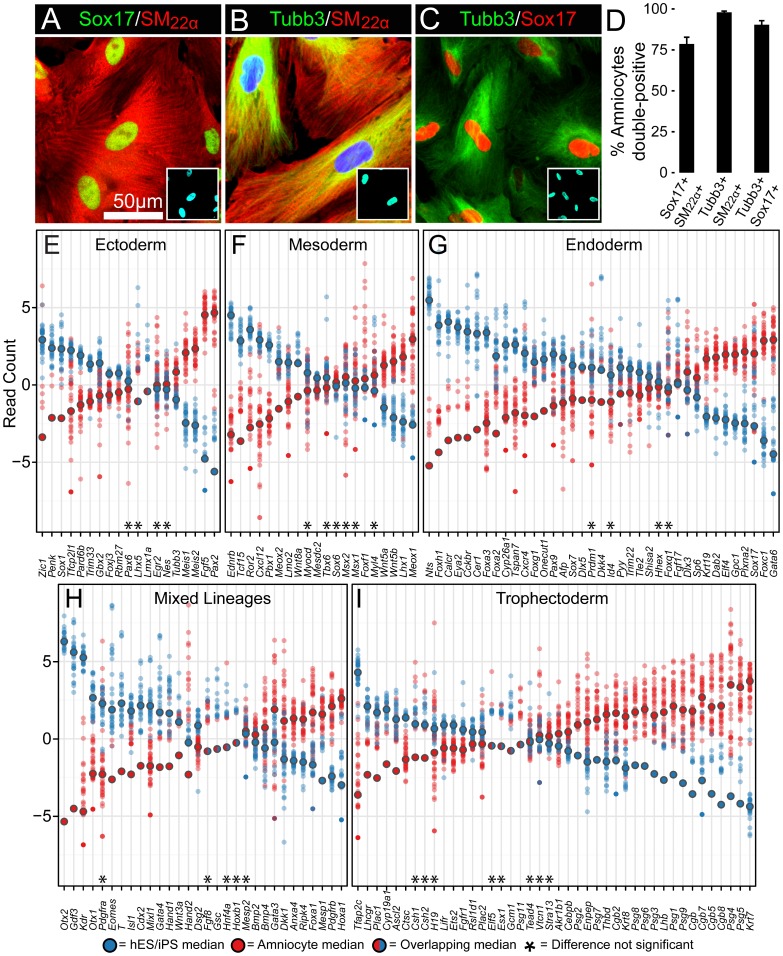
Amniocytes coexpress a complex phenotype that partially differentiates along multiple lineages. (A–C) Confocal images of amniocytes double-immunostained for well-defined lineage markers as indicated: Sox17; an endoderm marker, SM_22α_; a smooth muscle marker for mesoderm, and Tubb3; a neuronal marker for ectoderm. Hoechst dye was used to label nuclei (cyan-colored insets) in all panels. (D) The average percent of amniocytes per isolate that co-expressed lineage markers, ± standard error of mean. Over 75% of amniocytes were double-positive for two disparate lineage markers in multiple independent patient isolates: Sox17+/SM_22α_+(78.7%, n = 11); Tubb3+/SM_22α_+(98.0%, n = 11); Tubb3+/Sox17+ (90.4, n = 8). 4,998 cells were counted for all conditions. (E–I) Amniocytes show partial differentiation into each of the four embryonic primary lineages that is distinct from the partial differentiation seen in ESC and iPSC. Differential expression analysis of RNA-seq datasets reveals that lineage specification and potential in amniocytes differs from ESC/iPSC. Dot plots show RNA-seq read counts analyzed as in Figure 5. We examined 150 genes known to specify the three germ layers (E) ectoderm, (F) mesoderm, (G) endoderm, as well as (H) mixed lineages (enriched in derivatives of more than one germ layer) and (I) trophectoderm in mouse and human ES cells (Table S5). Asterisks = no statistical difference between amniocytes and ESC/iPSC (defined as the adjusted *p*-value is greater than 0.05).

Undifferentiated amniocytes are thought to contain small subsets of multipotent progenitor cells. To define how abundant these multipotent progenitor cells are in amniocyte isolates and their differentiation capability, we co-stained multiple patient isolates with well-defined germ layer markers that are considered definitive for endoderm (Sox17; an endodermal marker), mesoderm (SM_22α_; a smooth muscle marker), and ectoderm (Tubb3; a neuronal marker) (Figure 7A–D). Quite remarkably, over 75 percent of amniocytes were double-positive for two unrelated lineage-specific markers (Figure 7D), for example both mesoderm and endoderm or mesoderm and neural markers in the same cell. Given these intriguing results, lineage-specific differentiation in amniocytes may not be as rare, restricted, or independent as previously believed. The coexpression of multiple differentiation markers inside a large number of amniocytes may reflect an overlapping multilineage status of the entire population or it may be a distinct phenotype that sets this particular cell-type apart from all other known cell populations.

To further explore the multilineage differentiation phenotype of amniocytes, we examined expression of genes characteristic of three different germ layer fates in amniocytes, ESCs and iPSCs. We compiled a selected list of 150 genes known to play important roles in formation of the primary germ layers and trophectoderm. Since some of these 150 genes are enriched in multiple lineages, we included a fifth category of genes grouped as mixed lineages (Figure 7H; see Table S5 for supporting references).

There were clear differences between amniocytes and ESCs/iPSCs in the expression of markers for specific embryonic lineages. Surprisingly, amniocytes show *fewer* characteristics of the ectodermal, mesodermal, and endodermal lineages than ESC and iPSC lines (Figure 7E–G). For example, the important mesodermal markers *Eomes*, *Cdx2*, *Brachyury* (*T*), and *Goosecoid* (*Gsc*) were almost completely undetectable in amniocytes, whereas they are expressed at relatively high levels in ESC and iPSC lines. Although the median read count was located near zero for many lineage-specific genes, some samples of amniocytes expressed low levels or even relatively higher levels of specific transcripts. This variability in gene expression for germ layer markers indicates that certain amniocyte samples may have a greater degree of specification than other pluripotent cells types.

To determine whether undifferentiated amniocytes coexpress a trophectodermal phenotype, we co-stained Cgb1 (Choriogonadotropin subunit beta variant 1; a trophectoderm and placenta marker) with the other three germ layer markers. We did not detect protein expression of Cgb1 in five independent amniocyte patient isolates. However, when we examined a larger set of known trophectoderm-related genes by RNA-seq analysis, *Cgb1* and other Cgb family members had relatively high transcript levels in amniocytes. In fact, a majority of the trophectoderm markers were enriched in amniocytes (Figure 7I). Pregnancy-specific glycoprotein (*Psg*) is another classic marker of the human placenta [45]. Multiple Psg family members were enriched in amniocytes compared to ESC and iPSC lines. Taken together, these results suggest that amniocytes contain a fairly significant component of cells with trophectodermal characteristics, but they also contain progenitors that have characteristics of all three germ layers.

## Discussion

Since the developmental state of amniocytes is unclear, we sought to more precisely define the identity of this cell-type by immunostaining, flow cytometry, clonal analysis, qPCR, and RNA-seq whole-genome profiling. Our results demonstrate that amniocytes have a unique stem cell identity.

Previous studies argued that amniocytes are stem cells that exist in an intermediate state between pluripotency and lineage-specific restriction [15], [17], [20]. Recent microarray analyses performed on undifferentiated amniocytes reported a limited stem cell-like signature that can only be reverted to a more functional pluripotent state by DNA-integrating transgene [23] and transgene-free [18] approaches. Similar to Moschidou *et al.* [18], we found a large fraction of amniocytes expressing key pluripotency markers Oct4, Sox2, Nanog, and Klf4 and smaller subsets enriched for the stem cell markers such as SSEA1, SSEA3, SSEA4, Tra-1-60, and Tra-1-81. However, these studies significantly differ in the quantification of absolute percentages of these markers. It is unclear what factors could account for such significant differences, but earlier gestational aged amniocytes (10–12 G.A versus 15–36 G.A) or different culturing conditions might be two possibilities.

In a separate study by Ginsberg *et al.*, the amniocyte isolates were reported to lack Oct4 and Sox2 transcripts and protein, but the cell surface markers SSEA3 (31%), Tra-1-60 (17%), and Tra-1-80 (28%) were detectable by FACS analysis [33]. This study also performed RNA-seq analysis on a single isolate and did not detect the pluripotency factors *Oct4*, *Sox2*, *Nanog*, *Lin28*, *Sall4*, *Dppa*, *Utf1*, and *Esrrb*. In our larger RNA-seq dataset from 11 separate patients (37 replicates); the pluripotency marker Sox2 is detectable in some, albeit not all, amniocyte samples. *Dppa2*, *Utf1*, and *Esrrb* were absent. However, the critical pluripotency markers *Oct4*, *Nanog*, *Lin28*, and *Sall4* were reliably detected in most isolates. Similarly, Ginsberg *et al.* concluded that undifferentiated amniocytes are devoid of endothelial progenitor cells [33]. In contrast, our data from a large number of patient samples clearly shows expression of key lineage-specific regulators for both endothelial and smooth muscle cell-types. We suggest that the previous conclusions that amniocytes lack pluripotency or other progenitor cell types and can be directly transdifferentiated without benefit of a pluripotent state need to be reinterpreted in light of our findings of multiple pluripotent factors in a large number of amniocyte samples.

Based on evidence from a number of studies, a growing consensus suggested that amniocytes are a heterogeneous population expressing a specific set of pluripotency markers. However, due to small sampling sizes, intra-patient differences have been overlooked. In order to address the heterogeneity and variability of multiple genomes in a statistically rigorous manner, we designed our experimental analyses to include a large number of patient samples and several culture time-points, making our study more comprehensive than previous studies.

Importantly, although some amniocytes express the essential triumvirate of *Oct4*, *Sox2*, and *Nanog*, differential expression analyses show that transcript levels of these factors are an order of magnitude lower in amniocytes than in ES or iPS pluripotent cells, and in many isolates, *Sox2* is low or undetectable. When we examined an expanded set of 250 stem cell and pluripotency markers, amniocytes and ES/iPS cells shared expression of most of these factors. However, many of these markers are expressed at higher levels in ES/iPS cells, some at similar levels, and some at lower levels. The transcriptional dosage is therefore a key determinant controlling the distinct stem cell phenotype of amniocytes.

We interpret this observation to mean that amniocytes have a unique ground state that clearly differs from a true functional pluripotent state. Supporting this idea of pluripotent factor dosage, amniocytes treated with valproic acid (class I HDAC inhibitor) significantly upregulated Oct4, Nanog, and Sox2 levels relative to ESCs, and these cells were reprogrammed into a functional pluripotent state capable of forming teratoma-like masses in immunodeficient mice [18]. These authors also report that chemical reprogramming alone yielded amniocytes sharing an 82% transcriptome identity with hESC lines, yet hierarchical clustering still has reprogrammed amniocytes more closely related to untreated amniocytes than hESC lines. In contrast, our differential expression analyses show that iPSC lines (derived by standard transgene approaches) and hESC lines have a calculated genome-wide r-value of 0.99, much higher than valproic acid reprogrammed amniocytes. In the burgeoning field of iPSC reprogramming, evidence is lacking on what might be the minimum transcriptional and epigenetic changes that allow amniocytes to efficiently convert to a functional pluripotent state.

Gestational age and time in culture are major influences on the extent of stem cell characteristics in amniocytes. During fetal development, the cell-type profile in amniotic fluid varies. The mechanisms responsible for shedding fetal and placental cells into the amniotic cavity are poorly understood, but might relate to the changing fluid dynamics of diverse epithelial surfaces [2]. As fetal development progresses, pluripotency becomes more restricted. We therefore expected earlier gestational ages would contain larger stem cell populations than later gestational ages. Contrary to what we hypothesized, *Nanog* transcript levels increased with gestational age.

Gestational-influence on stem cell status could result from an expanding pool of amniocytes or a stable pool that is undergoing epigenetic reprogramming, allowing the reacquisition of pluripotency. Recent work highlights how extended culturing of human ES cells leads to progressive accumulations of both genetic and epigenetic changes [46]. Elevated levels of many senescence-associated genes in amniocytes slow proliferation rates over multiple passages [23]. In contrast, long term cultures of amniocytes in another study showed no signs of senescence or slower rates of proliferation [18]. In our study, longer culture times appear to select for an increase in stem cell markers, while maintaining a distinct set of highly expressed senescence-associated genes. We noticed that proliferation rates in different amniocyte samples are highly variable; some samples exhibiting an indefinite length of exponential growth, while other samples senesce abruptly without any obvious explanation.

Despite the different rates of growth kinetics observed among these studies, our longitudinal analysis of cultured amniocytes suggests longer culture times strongly influence global gene expression. These expression features may reflect an increase in the percentage of cells occupying a stem-like state over time, as more differentiated cell types either exit the cell cycle or exhibit a slower rate of proliferation. It is important to note that technical limitations confound the interpretation that culture time correlates with changes in gene expression. Variable diagnostic testing and release times, changes in media conditions, and natural variability across different patient isolates could influence gene expression profiles. Most likely, each of these variables imparts a specific effect on gene expression that should be considered experimentally when designing protocols examining human amniocytes.

Amniocytes have characteristics of both embryonic and extraembryonic derivatives. Based on our genome-wide analysis, amniocyte developmental state is relatively primitive, suggesting an immature state, rather than a bona fide multilineage commitment. Despite expressing some markers suggestive of commitment, most transcription factors and functional proteins that define lineage-committed and mature cell-types are extremely low or unexpressed in amniocytes. Moreover, compared to tissue-specific patterns, amniocytes lack expression of key regulatory genes for primary germ layers, indicating that they are not committed to a particular lineage.

Recognition that different types of cytoskeletal filaments are expressed within amniocyte subpopulations suggested that they originate from different tissues [47]–[50] and even potentially from all three germ layers [2], [51], [52]. However, testing this hypothesis experimentally has been difficult, and it remains an open question [53]. It is not known whether residual precursor cells within this heterogeneous population can give rise to these germ layer lineages *de novo*. Recent studies suggested that amniocytes generally do not express any standard markers affiliated with embryonic germ layers, and that germ layer markers can be upregulated through combinations of reprogramming, embryoid body differentiation, or directed differentiation [17], [18], [20], [21], [23], [30], [33].

In contrast, our genome-wide analysis reveals that second trimester amniocytes already express many of these same germ layer specific markers at robust levels, and do not require any additional manipulation in order to activate these developmental pathways. Second, our co-staining analysis (Fig. 7A–D) found that most amniocytes express multiple germ layer markers, demonstrating that a majority of amniocytes are in a baseline differentiation state that is quite ambiguous. Third, our clonal analysis from seven different patients found that individual amniocyte clones are capable of self-renewal and give rise to a heterogeneous population of progeny that have similar properties to the parent population. For example, clonal cells give rise to multiple categories of SSEA4 and Tra-1-60 expression (both on; both off; only one on) in the clonal progeny population. Together, these observations indicate that amniocytes are capable of self-renewal and generation of the mixed populations, and that the heterogeneity seen in multiple patient isolates is not a static representation of contributions to amniotic fluid from different embryonic sources, but that the amniocyte pluripotent heterogeneity is dynamically generated and maintained by amniocyte populations themselves. Thus, the multilineage potential of amniocytes might not be as clear-cut as previously interpreted. The ambiguity of amniocytes' molecular and phenotypic signatures might represent a specific differentiation state that is tightly regulated.

Alternatively, some of this distinct phenotype in amniocytes might be related to culture artifacts. Post-amniocentesis culturing might alter the differentiation state of amniocytes, dramatically changing their molecular and phenotypic makeup. We believe this possibility is less likely because amniocytes retain their core phenotype even after manipulating them with a variety of recombinant proteins and potent chemical inhibitors and activators (not shown). We conclude that the amniocytes' complex molecular and phenotypic signature reflects an early developmental state that is partially differentiated, but uncommitted.

Amniocytes have been hypothesized to be derived primarily from embryonic endoderm, trophectoderm, and possibly from primordial germ cells [18], [51], yet they share an expression signature that only partially matches these lineages. For example, amniocytes express relatively high levels of *Gata6* and *Sox17*, markers typically associated with the definitive endoderm [54] and extraembryonic primitive endoderm [55], [56]. In contrast, many other important endodermal markers are expressed at comparably low levels in amniocytes (see Figure 7). Furthermore, amniocytes do not phenocopy a true trophoblast identity because the key trophoblast stem cell markers *Elf5*, *Cdx2*, and *Eomes* are mostly undetectable. A recent study proposes that amniocytes share common gene expression profile with primordial germ cells [18]. In our large RNA-seq dataset, many key markers for primordial germ cells are not expressed, arguing against a population of amniocytes being derived from primordial germ cells. Alternatively, the primordial germ cell markers that are expressed in amniocytes might be related to these genes playing functional roles in multiple lineages besides germ cells. Based on our genome-wide analyses, we conclude that the expression signature of amniocytes is distinct from ES and other pluripotent cells. Thus, absence of key lineage markers suggests that amniocytes are not locked into lineages from which they might have been derived.

Amniotic fluid contains a complex milieu of nutrients and signaling molecules [39] that could potentially influence gene expression. Free-floating amniocytes may not be specified by the same repertoire of developmental signals as resident stem cells inside tissue, resulting in an expression signature that is unlike any other lineage. Nonetheless, our genome-wide expression study reveals that a transcriptional footprint of certain developmental regulators may be retained in amniocytes, giving a clue to the original anatomical sites of cell origin. For example, amniocytes exhibit a specific pattern of *Hox* and *Fox* genes that are consistently expressed at high levels. However, many of these highly expressed genes are erased when amniocytes are induced into a pluripotent state [23].

A surprising feature of our analysis is the large degree of lineage-specific gene expression observed in both amniocytes and in pluripotent ESC/iPSC lines (see Figure 7). Pluripotent ESC and iPSC lines are routinely derived from pre-implantation embryos or from molecular reprogramming of somatic cells [57], [58]. Despite some small differences between ESC and iPSC lines [59], both are thought to exist in a fully unrestricted ground state that remains unprimed for lineage specification and commitment. However, pluripotent stem cells are transcriptionally and epigenetically heterogeneous [60]–[63]. Their inherent heterogeneity has spurred researchers to ask whether pluripotent stem cells contain multiple metastable subpopulations that exhibit distinct differentiation biases [64]–[66]. Our genome-wide analysis provides striking evidence that pluripotent ESC and iPSC lines exhibit a transcriptional signature that has partially differentiated, countering the prevailing view that these cells are entirely unspecified.

Previous studies have asserted that a variety of key embryonic germ layer markers are unexpressed in undifferentiated amniocytes and then upregulated in response to differentiation stimuli. However, using more sensitive RNA-seq analyses, we demonstrate that many of these markers are expressed unambiguously in undifferentiated amniocytes at baseline [18], [23]. We therefore conclude that the question is not whether these key genes are on or off, but at what levels and to what extent these levels change upon differentiation.

Amniocytes also have an expression signature suggestive of partial differentiation, but it is clearly dissimilar from ESC and iPSC lines. Thus, both amniocytes and pluripotent ESC/iPSC lines may have undergone distinct forms of multilineage priming, a mechanism that could explain their precocious transcription of developmental genes. Alternatively, naïve pluripotent stem cells accumulate paused RNA polymerase II on key regulatory promoters, allowing cells to quickly respond to developmental cues and transcriptional potentiation [67]. The promiscuous transcription of some lineage-affiliated genes in amniocytes may therefore be unrelated to multilineage priming, but to a uniquely permissive, yet repressed chromatin state. Determining the epigenetic landscape in amniocytes, particularly for key lineage regulators, might help better define their developmental status and potency.

Transcriptional repression is an important feature of the developmental status of amniocytes. We demonstrate that amniocytes express a profile of repressive factors that is distinct from other pluripotent cells. Some of these repression factors were the highest differentially enriched genes in our genome-wide analyses. The unique dynamics of amniocyte differentiation are likely to depend on both transcriptional and epigenetic regulation. Little is known about epigenetic status in amniocytes. However, a recent study using array-based profiling determined that DNA-methylation levels in amniocytes are extremely high and many genes enriched with low CpG content are hypermethylated [68]. Linking highly expressed transcriptional repressors to specific epigenetic marks in amniocytes will deepen our understanding of their developmental potency. Preliminary studies in our laboratory indicate that directing amniocytes toward specific lineages fails to reduce highly expressed repression markers. Therefore, the high level of repression might need to be overcome by driving amniocytes completely back to primitive functional pluripotency. In some cases, the repressive state in amniocytes might be advantageous for direct reprogramming approaches, driving them down specific lineages.

### Conclusions

Our results reveal that amniocytes have a unique stem cell identity. The stem cell state in amniocytes shows greater heterogeneity and variability in gene expression than previously appreciated. Genome-wide, amniocytes express many of the same genes as undifferentiated pluripotent cells. However, core pluripotent genes are expressed at disproportionately lower levels in amniocytes, a key feature that distinguishes their expression signature from true pluripotent cells. The stem cell state in amniocytes is therefore transcriptionally distinct, as is their gene lineage program that allows partial differentiation into the four primary embryonic germ layers. Low levels of crucial lineage markers and high levels of repressor markers indicate that amniocytes exists in a developmentally intermediate yet uncommitted state.

## Materials and Methods

### IRB Review of Study

The University of Utah IRB determined that our project (IRB_00040970) did not meet the definitions of Human Subjects Research according to Federal regulations and IRB oversight was therefore not required.

### Human Amniocytes and Stem Cell Culture

Patient de-identified, cytogenetically normal human amniocytes were obtained from ARUP laboratories (Salt Lake City, Utah), after having been cultured in AmnioMAX (Invitrogen: 12558) for two to four weeks. Once we received samples from ARUP, the media was replaced with Knockout D-MEM (Invitrogen: 10829-018) with 15% BenchMark FBS (Gemini-Bio: 100–106), 0.4 mM 1-Thioglycerol (Sigma: M6145), 0.1 mM 2-Mercaptoethanol (Millipore: ES-007-E), 1% GlutaMAX (Invitrogen: 35050), and 1% Penicillin/Streptomycin, and cultures were maintained at 5% CO2. We used 80 independent amniocyte isolates from gestational weeks 15–17, 25, and 30, with some samples used for multiple protocols (Table S6). hESCs and hIPSCs cell lines used in the RNA-seq differential expression analysis were cultured in supplemented DMEM and maintained in a feeder-independent system as described by Phanstiel et al [41].

### Clonal analysis

Amniocytes were dissociated with TrypLE Express (Invitrogen Cat# 12604-013) for 15 minutes and mechanically dissociated until a majority of cells were singly suspended in solution. Single amniocytes were cloned by limiting dilution placed in single wells of 96-well place and allowed to expand for one week.

### Immunohistochemistry and Confocal Microscopy

Amniocyte cell lines that had reached confluency were dissociated using Accutase (Sigma: A6964), counted using the Scepter handheld automated cell counter (Millipore, Bellrica, MA), and replated in either 6-well plates or 24-well plates. Cells were fixed in 4% paraformaldehyde (PFA) for 5 min, and either not permeabilized or permeabilized (depending on the antibody) with PBS, 0.1% BSA, and 0.2% Triton X-100 for 30 minutes. Cultures were stained with primary antibodies (see Table S6) in PBS and 0.1% BSA for 30 minutes, washed twice with PBS, incubated 30 minutes with secondary antibodies (Invitrogen: AlexaFluor in PBS and 0.1% BSA) and washed twice with PBS. Nuclei were counterstained using 1.8 mM Hoechst in PBS (Invitrogen: H21486). Cultures were visualized using an Olympus FV1000/IX81 confocal microscope (Olympus America Inc., Center Valley, PA) with an Olympus LUCPlanFLN 20× objective lens (numerical aperture, 0.45). Blue, green, and red fluorescent probes were excited using a 405 nm-line LD laser (425–475 band pass), a 488 nm-multiline argon laser (500–530 band pass), and a 543 nm-line helium/neon-red laser (BA560IF filter set).

### Image Processing

Confocal images were acquired in TIFF format as either single images or stacks, converted into average Z-plane projections using ImageJ software (NIH, Bethesda, MD) and merged to produce single pseudo-colored images. Cell counting was performed by an observer blinded to the experimental conditions, using three to five random images per well for each sample. Positive nuclei were counted if the entire nucleus could be delineated, and confirmed with an overlapping nuclear dye counterstain. For the surface stem cell markers, cells were scored as positive if the entire surface extending to the lateral border was evenly stained.

### qPCR (real-time PCR)

Total RNA was isolated using Absolute RNA Microprep Kit (Agilent Stratagene: 400805) or RNeasy Mini Kit (Qiagen: 74104) and measured on a NanoDrop 2000c spectrophotometer (Thermo Scientific). qPCR reactions were mixed with cDNAs (Invitrogen: 18080-51), 10 µM SYBR Green (Qiagen: 204072) and primer sets (designed by Roche's Universal ProbeLibrary Assay Design Center, made by Operon's PCReady PCR & Sequencing Primers; see Table S6). PCR reactions were run with reference gene GAPD-Human (Roche: 05190541001) on a LightCycler 480 (Roche). Raw Cp values were generated by running the absolute quantification/2nd Derivative Max setting. Relative gene expression was calculated as raw Cp values for genes from individual patients and normalized to the Cp value of Gapdh. We followed the MIQE guidelines for evaluating qPCR results [69]. For hierarchical clustering analysis of qPCR raw Cp values, we used Pearson's r [distance = 1− |r|] to calculate the pairwise correlation distance among several pluripotency genes (+Gapdh). This analysis calculated the strength of linear dependence of all gene Cp values and is not affected by different amplification efficiencies.

### RNA-seq

We performed longitudinal culture studies by serially splitting isolates at an average culture time of 1.3 days (T1), 15.2 days (T2), and 28 days (T3). Some patient samples arrived from the ARUP laboratories as extremely small isolates and were grown for a few days to increase cell number before the first time point could be collected. RNA was isolated as above and analyzed using a Bioanalyzer 2100 (Agilent Technologies) to verify RNA integrity. 1.5 µg of total RNA from each sample was converted to mRNA-seq library at the CvDC RNA Expression Core using Illumina Tru-seq kit (Illumina: FC-122-1001). Libraries were sequenced on a HiSeq 2000 (Illumina), containing three barcoded samples per lane. Sequences were aligned to human genome build hg19 using Novoalign V2.07.10 (Novocraft Technologies). Useq software [70] was used to convert splice junction read coordinates and to count reads that overlap annotated genes (Ensgene annotation) (for a summary of RNA-seq reads see Table S6).

High-quality publically available RNA-seq datasets containing raw files for 11 samples (32 replicates) were downloaded from Stem Cell-Omics Repository (SCOR http://scor.chem.wisc.edu). These RNA-seq datasets were generated from 1) four human ESC lines (H1, H7, H9, and H14 cell lines); 2) three iPSC lines (DF4.7, DF6.9, DF19.7); and 3) a single iPSC parental newborn foreskin fibroblasts (NFF). Two additional RNA-seq datasets generated from H1 cell lines were downloaded from Gene Expression Omnibus (GEO).

Differential expression analyses were conducted in R [71] using the DESeq package [72]. Negative binomial *p*-values are based on dispersions estimated separately for each gene, treating all samples as biological replicates. *P*-values are adjusted to control for false discovery rate (FDR) within each comparison. Hierarchical clustering and bootstrapping were performed with the pvclust package for R [73]. Data were median centered by gene before clustering. Bootstrap *p*-values are reported as percentages and are based on 10,000 randomly resampled replicates unless otherwise noted. For all analyses, RNA-Seq reads are size factor corrected to allow comparison of samples with varying read depth. Additionally, for clustering and dot plots, read counts were subjected to a variance-stabilizing transformation as described by [72]. No correction for transcript length was performed.

### Flow Cytometry

Confluent amniocytes were dissociated with Accutase (Sigma: A6964) for 30 minutes, filtered using 40 µm cell strainer (BD Falcon: 352340), fixed with 4% PFA for 10 minutes, and washed 1× PBS. Cells were then stained with primary antibodies SSEA1 and SSEA4, as above. PBS suspensions of amniocytes were run on a Flow Cytometer (BD Biosciences: BD FACSAria II Flow Cytometer), using BD FACSdiva analysis software (BD biosciences: FACSDiva Version 6.1.3).

### Data Access

All 37 amniocyte RNA-seq datasets (raw FASTQ and post-processed BAM files) created for this study are publicly available at the GNomEx CvDC (Cardiovascular Development Consortium) link: https://b2b.hci.utah.edu/gnomex/gnomexGuestFlex.jsp?topicNumber=39

## Supporting Information

Figure S1 **Hierarchical clustering analysis of all samples.** RNA-seq datasets were generated from 1) amniocytes, 2) ESC (H1, H7, H9, and H14 cell lines); 2) iPSC; and 3) iPSC parental newborn foreskin fibroblasts (NFF). 71 samples clustered by variance-stabilized mRNA−seq read counts of 24,612 genes (mean read count >4). Bootstrapping values are based on 100 randomized replicates. Correlation distance is 1−|Pearson's r|.
(PDF)

Table S1 **Statistical analysis of selected stem cell markers.** (A) Statistical analysis of RNA-seq read counts comparing gestational age, time in culture (T1/T3), and gender using the negative binomial test provided by DESeq (1). * indicates FDR adjusted *p*-value less than 0.05. (B) pPCR Cp values (normalized to GAPDH) comparing gestational age, time in culture (T1/T3), and gender using the Wilcoxon rank sum test provided by R (2). * indicates FDR adjusted *p*-value less than 0.05.
(PDF)

Table S2 **Reference list for 250 stem cell markers.** The 250 stem cell markers were included in our list based on the following two criteria; 1) each stem cell marker has a reported functional analysis in stem cells and 2) each gene was reliably detected at significant levels in our RNA-seq dataset for amniocytes or ESC and iPSC.
(PDF)

Table S3 **RNA-seq differential expression analysis.** Table S3A shows transcripts identified by RNA-seq analysis (1) that were (A) consistently higher in ESC or (B) consistently higher in iPSC compared to amniocytes. Table S3B shows differential expression results that were sorted by the smallest to largest adjusted *p*-value. The top ten most significant genes for each comparison are listed.
(PDF)

Table S4 **Reference list for 89 embryonic stem cell repressors.** The 89 putative repressors were included in our list based on the following two criteria; 1) each gene has a reported repressive function during development and 2) each gene was reliably detected at significant levels in our RNA-seq dataset.
(PDF)

Table S5 **Reference list of 150 genes for primary embryonic lineages.** The 150 genes were included in our list based on the criteria; 1) previous reports show the gene playing a possible functional role in any of the three primary germ layers or in the trophectodermal lineage during development and 2) each gene was reliably detected at significant levels in our RNA-seq dataset.
(PDF)

Table S6 **Timeline and patient samples used in study, related to** **Figures 1** **–** **7** **.** Panel A is the experimental timeline that depicts time since amniocentesis, culture time at ARUP Labs (red bar) and culture time in our lab (green bar). Table S6B is a summary of total patient samples used in this study. (Patient samples were de-identified and new patient numbers were given in a general order they were received). (X marks) in table indicates type of analysis tested for each sample. Table S6C summarizes RNA-seq samples and read counts. Table S6D is a complete list of antibodies used in this study. Table S6E is a complete list of primers used in this study.
(PDF)
