# Supplementary material for: Genome-Wide Analysis Reveals the Unique Stem Cell Identity of Human Amniocytes
Source: PLoS One. 2013 Jan 10;8(1):e53372. doi: 10.1371/journal.pone.0053372 (PMC3542377; doi:10.1371/journal.pone.0053372)
Supplement: Figure S1 — Hierarchical clustering analysis of all samples. RNA-seq datasets were generated from 1) amniocytes, 2) ESC (H1, H7, H9, and H14 cell lines); 2) iPSC; and 3) iPSC parental newborn foreskin fibroblasts (NFF). 71 samples clustered by variance-stabilized mRNA−seq read counts of 24,612 genes (mean read count >4). Bootstrapping values are based on 100 randomized replicates. Correlation distance is 1−|Pearson's r|. (PDF) [file pone.0053372.s001.pdf]

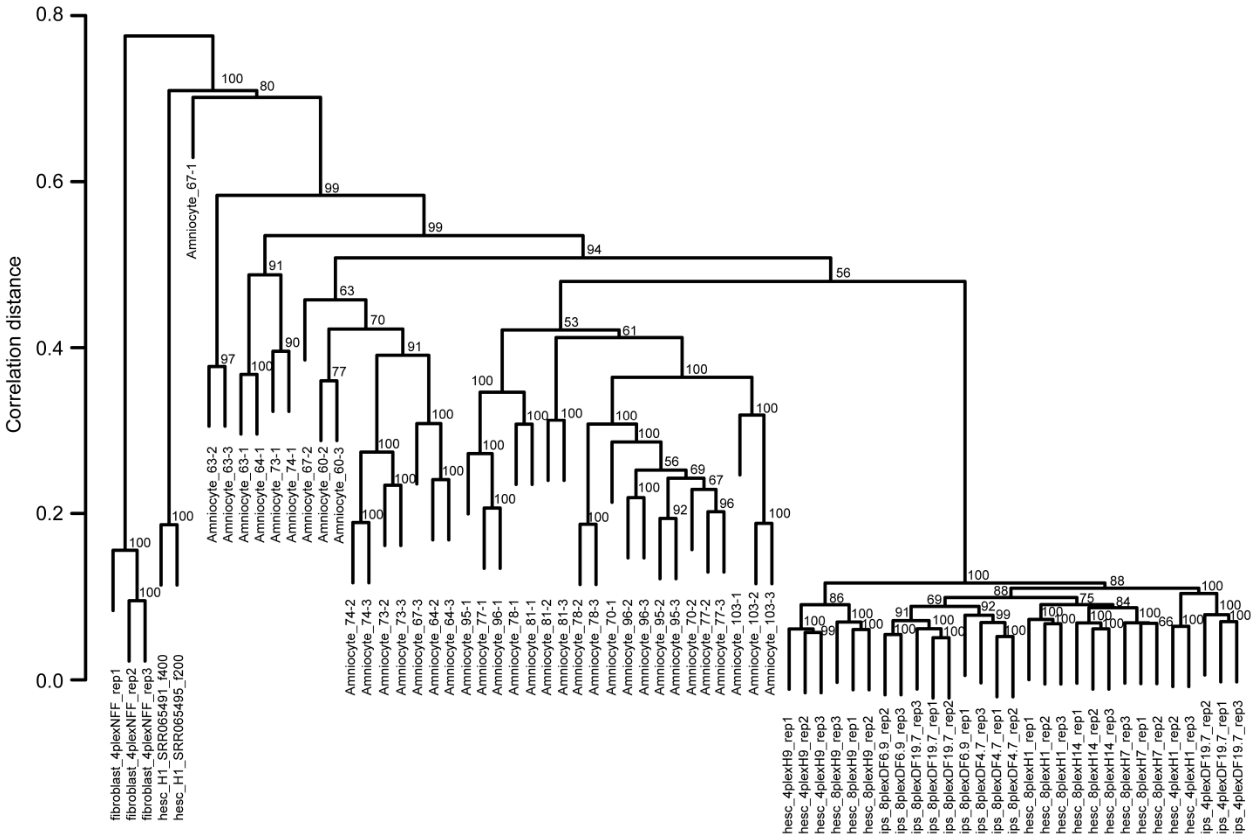

Figure S1. Hierarchical clustering analysis of all samples. RNA-seq datasets were generated from 1) amniocytes, 2) ESC (H1, H7, H9, and H14 cell lines); 2) iPSC; and 3) iPSC parental newborn foreskin fibroblasts (NFF). 71 samples clustered by variance-stabilized mRNA−seq read counts of 24,612 genes (mean read count > 4). Bootstrapping values are based on 100 randomized replicates. Correlation distance is 1 - | Pearson’s r |.
