# Supplementary material for: Genome-Wide Analysis Reveals the Unique Stem Cell Identity of Human Amniocytes
Source: PLoS One. 2013 Jan 10;8(1):e53372. doi: 10.1371/journal.pone.0053372 (PMC3542377; doi:10.1371/journal.pone.0053372)
Supplement: Table S1 — Statistical analysis of selected stem cell markers. (A) Statistical analysis of RNA-seq read counts comparing gestational age, time in culture (T1/T3), and gender using the negative binomial test provided by DESeq (1). * indicates FDR adjusted p-value less than 0.05. (B) pPCR Cp values (normalized to GAPDH) comparing gestational age, time in culture (T1/T3), and gender using the Wilcoxon rank sum test provided by R (2). * indicates FDR adjusted p-value less than 0.05. (PDF) [file pone.0053372.s002.pdf]

Table S1. Statistical analysis of selected stem cell markers. (A) Statistical analysis of RNA-seq read counts comparing gestational age, time in culture (T1/T3), and gender using the negative binomial test provided by DESeq [1]. * indicates FDR adjusted p-value less than 0.05. (B) pPCR Cp values (normalized to GAPDH) comparing gestational age, time in culture (T1/T3), and gender using the Wilcoxon rank sum test provided by R [2]. * indicates FDR adjusted p-value less than 0.05.

**Genes**	**Gestational Age (young vs. old)**	**Time in Culture (T1 vs. T3)**	**Gender (Male vs. Female)**
**(A) RNA-seq**			
Oct4	0.148	0.959	0.900
Sox2	0.582	1.000	0.960
Nanog	0.041* higher in old	0.952	0.992
Klf4	0.853	0.308	0.941
Wdr5	1.000	0.579	0.842
Fut4	0.578	0.600	0.966
Ckit	0.875	0.980	0.638
Gapdh	0.782	0.487	0.568
**(B) qPCR**			
Oct4	0.661	0.612	0.661
Sox2	0.612	0.661	0.962
Nanog	0.661	0.612	0.962
Klf4	0.661	0.612	0.661
Wdr5	0.612	0.612	0.612
Fut4	0.661	0.661	0.661
Ckit	0.661	0.661	0.661
Gapdh	0.661	0.612	0.612

REFERENCES

1. Anders S, Huber W (2010) Differential expression analysis for sequence count data. Genome Biol 11: R106.

2. R Development Core Team (2012) R: A language and environment for statistical computing. R Foundation for Statistical Computing, Vienna, Austria. ISBN 3-900051-07-0, URL http://www.r-project.org/.
