# Supplementary material for: Genome-Wide Analysis Reveals the Unique Stem Cell Identity of Human Amniocytes
Source: PLoS One. 2013 Jan 10;8(1):e53372. doi: 10.1371/journal.pone.0053372 (PMC3542377; doi:10.1371/journal.pone.0053372)
Supplement: Table S2 — Reference list for 250 stem cell markers. The 250 stem cell markers were included in our list based on the following two criteria; 1) each stem cell marker has a reported functional analysis in stem cells and 2) each gene was reliably detected at significant levels in our RNA-seq dataset for amniocytes or ESC and iPSC. (PDF) [file pone.0053372.s003.pdf]

Table S2. Reference list for 250 stem cell markers. The 250 stem cell markers were included in our list based on the following two criteria; 1) each stem cell marker has a reported functional analysis in stem cells and 2) each gene was reliably detected at significant levels in our RNA-seq dataset for amniocytes or ESC and iPSC.

Gene_ Symbol	Ensgene_id	Refseq_id	Gene_Function	References
Acvr2b	ENSG00000114739	NM_001106	Receptor for activin A, activin B and inhibin A	[1,2]
Alcam	ENSG00000170017	NM_001627	Cell adhesion, leukocytes, neural, cardiomyocytes	[3,4]
Arid1b	ENSG00000049618	NM_017519	Trithorax group	[5]
Ars2	ENSG00000087087	NM_182800	ESC identity	[6,7]
Ash2l	ENSG00000129691	NM_004674	Trithorax group	[8]
Axin2	ENSG00000168646	NM_004655	Wnt signaling	[9]
Bmi1	ENSG00000168283	NM_005180.8	Polycomb group	[10]
Brix	ENSG00000113460	NM_018321	Biogenesis of ribosomes	[11]
Cbx1	ENSG00000108468	NM_006807	Polycomb group	[12]
Cbx5	ENSG00000094916	NM_001127321	Chromatin regulator	[12]
Ccna1	ENSG00000133101	NM_003914	Proliferation	[13]
Ccnd1	ENSG00000110092	NM_053056	Proliferation	[13,14]
Ccnd2	ENSG00000118971	NM_001759	Proliferation	[13,14]
Ccne1	ENSG00000105173	NM_001238	Proliferation	[13,14]
Ccnf	ENSG00000162063	NM_001761	Proliferation	[15]
Cd24	ENSG00000185275	NM_013230	B-cell activation responses	[16]
Cd44	ENSG00000026508	NM_000610.3	Receptor for hyaluronic acid	[17]
Cd9	ENSG00000010278	NM_001769	Platelet activation	[18]
Cdh3	ENSG00000062038	NM_001793	Adhesion	[18]
Cdk2	ENSG00000123374	NM_001798	Proliferation	[13,14]
Cdk4	ENSG00000135446	NM_000075	Proliferation	[13,14]
Cdk6	ENSG00000105810	NM_001145306	Proliferation	[13]
Cdkn1b	ENSG00000111276	NM_004064	Proliferation	[13,19]
Cdyl	ENSG00000153046	NR_026590	Transcriptional repressor	[20]
Chd1	ENSG00000153922	NM_001270	Nucleosome mobilization	[21]
Chd7	ENSG00000171316	NM_017780	Nucleosome mobilization	[22,23]
Cks1b	ENSG00000173207	NM_001826	Proliferation	[24,25]

Cldn6	ENSG00000184697	NM_021195	ESC marker	[25]
Cnot1	ENSG00000125107	NM_016284	ESC identity	[26]
Cnot2	ENSG00000111596	NM_001199302	ESC identity	[26]
Cnot3	ENSG00000088038	NM_014516	Myc/Zfx cofactor	[26]
Cops2	ENSG00000113387	NM_001143887	Cop9 signalsome	[27]
Cops4	ENSG00000138663	NM_001258006	Cop9 signalsome	[27]
Cpsf3	ENSG00000119203	NM_016207	mRNA precursor processor	[28]
Crabp1	ENSG00000166426	NM_004378	Retinoic acid regulation	[29]
Dazap1	ENSG00000071626	NM_018959	RNA-binding protein	[28]
Dnmt3b	ENSG00000088305	NM_001207055	DNA methylation	[25]
Dppa2	ENSG00000163530	NM_138815	Pluripotency	[30]
Dppa3	ENSG00000187569	NM_199286	Pre-Postimplantation/ Germline determination	[31,32]
Dppa4	ENSG00000121570	NM_018189	Pluripotency	[30]
Dppa5	ENSG00000203909	NM_001025290	Pluripotency	[33]
Dpy30	ENSG00000162961	NM_032574	Epigenetic methylation	[34,35]
E2f1	ENSG00000101412	NM_005225	Transcription regulator	[36]
Eed	ENSG00000074266	NM_003797	Polycomb group	[37]
Ehmt2	ENSG00000204371	NM_006709	Chromatin regulator	[38]
Eif2b1	ENSG00000111361	NM_001414	EIF complex	[27]
Eif2b2	ENSG00000119718	NM_014239	EIF complex	[27]
Eif2b3	ENSG00000070785	NM_001166588	EIF complex	[27]
Eif2s2	ENSG00000125977	NM_003908	EIF complex	[27]
Epcam	ENSG00000119888	NM_002354	Adhesion	[18]
Eras	ENSG00000187682	NM_181532	Self-renewal	[39]
Esrrb	ENSG00000119715	NM_004452	Steriod hormone receptor	[40,41]
Ewsr1	ENSG00000182944	NM_001163285	Transcription regulator	[42]
Ezh1	ENSG00000108799	NM_001991	Polycomb group	[43]
Ezh2	ENSG00000106462	NM_001203247	Polycomb group	[44]
Fbxo15	ENSG00000141665	NM_001142958	Transcription regulator	[45]
Fgf13	ENSG00000129682	NM_004114	Differentiation	[46]
Fgf4	ENSG00000075388	NM_002007	Segregation	[47]
Flt3	ENSG00000122025	NM_004119	Receptor for fms-related tyrosine kinase	[48]

Foxd3	ENSG00000187140	NM_012183	ECS repressor	[49]
Foxh1	ENSG00000160973	NM_003923	Transcriptional regulator	[50]
Fry	ENSG00000073910	NM_023037		
Fut4	ENSG00000196371	NM_002033	Catalyzes synthesis of SSEA1	[51]
Fut9	ENSG00000172461	NM_006581	Catalyzes synthesis of SSEA1	[51]
Gabrb3	ENSG00000166206	NM_000814	Ligand-gated ionic channel	[11]
Gal	ENSG00000069482	NM_015973	Cellular messenger	[52]
Gbx2	ENSG00000168505	NM_001485	Transcriptional regulator	[53]
Gdf3	ENSG00000184344	NM_020634	Cell growth and differentiation	[54]
Gja1	ENSG00000152661	NM_000165	Cell-cell communication	[55]
Gli1	ENSG00000111087	NM_001167609	Transcriptional regulator	[56]
Gli2	ENSG00000074047	NM_005270	Transcriptional regulator	[56]
Gli3	ENSG00000106571	NM_000168.5	ESC repressor	[56]
Glis1	ENSG00000174332	NM_147193	Transcriptional regulator	[57]
Gnl3	ENSG00000163938	NM_014366.4	Proliferation	[58]
Grb7	ENSG00000141738	NM_001030002	RNA-binding protein	[59,60]
H2afz	ENSG00000164032	NM_002106	Chromatin regulator	[61]
Has2	ENSG00000170961	NM_005328	Hyaluronan/hyaluronic acid synthesis	[62]
Hcfc1	ENSG00000172534	NM_005334	Proliferation	[63]
Herc5	ENSG00000138646	NM_016323	E3 ligase	[64]
Hesx1	ENSG00000163666	NM_003865	Transcriptional repressor	[25,65]
Hira	ENSG00000100084	NM_003325	Histone cell cycle regulator	[28]
Hmga1	ENSG00000137309	NM_002131	Transcriptional regulation	[25]
Hspa4	ENSG00000170606	NM_198431	Heat shock protein	[66]
Hspb1	ENSG00000106211	NM_001540	Heat shock protein	[67]
Id1	ENSG00000125968	NM_002165	ESC identity	[68]
Id2	ENSG00000115738	NM_002166	ESC identity	[68]
Igf2bp1	ENSG00000159217	NM_001160423	Proliferation	[69,70]

Ing5	ENSG00000168395	NM_032329	Transcriptional regulator	[28]
Itga6	ENSG00000091409	NM_001079818	ECM receptor	[71,72]
Jarid2	ENSG00000008083	NM_004973	Histone methyltransferase regulator/ pluripotency	[73]
Kat2a	ENSG00000108773	NM_021078	Chromatin regulator	[74,75]
Kat5	ENSG00000172977	NM_182710	Histone acetylation	[76,77]
Kat6a	ENSG00000083168	NM_001099413	Histone acetylation	[78,79]
Kdm1a	ENSG00000004487	NM_001009999	Proliferation	[80]
Kdm3a	ENSG00000115548	NM_001146688	Cofactors	[81,82]
Kdm4a	ENSG00000066135	NM_014663	Cofactors	[83]
Kdm4c	ENSG00000107077	NM_001146694	Cofactors	[83]
Kdm5b	ENSG00000117139	NM_006618	Proliferation	[84,85]
Kit	ENSG00000157404	NM_000222	Stem cell factor receptor	[86,87]
Kitlg	ENSG00000049130	NM_000899	Pleiotropic stem cell factor	[88]
Klf12	ENSG00000118922	NM_016285	ES Identity	[89]
Klf2	ENSG00000127528	NM_016270	Transcription regulator	[90,91]
Klf4	ENSG00000136826	NM_004235	Transcription regulator	[90,91]
Klf5	ENSG00000102554	NM_001730	Transcription regulator	[90,92,93]
L1td1	ENSG00000240563	NM_001164835	MicroRNA processor	[94,95]
Lefty1	ENSG00000243709	NM_020997	Left-right asymmetry determination	[91,96]
Lefty2	ENSG00000143768	NM_003240	Left-right asymmetry determination	[97,98]
Lin28a	ENSG00000131914	NM_024674	MicroRNA processor	[12,25,99]
Lin28b	ENSG00000187772	NM_001004317	MicroRNA processor	[100,101]
Ly6e	ENSG00000160932	NM_001127213	Adhesion and signaling	[102]
Mapk1	ENSG00000100030	NM_002745	Pleiotropic cellular processes	[47]
Max	ENSG00000125952	NM_002382	Transcription regulator	[103,104]
Mcm2	ENSG00000073111	NM_004526.2	Replicative	[105]

			helicase factor	
Mcrs1	ENSG00000187778	NM_001012300	INO80 complex	[27,106]
Med1	ENSG00000125686	NM_004774	Mediator complex	[107,108]
Med10	ENSG00000133398	NM_032286	Mediator complex	[109]
Med12	ENSG00000184634	NM_005120	Mediator complex	[110]
Med13	ENSG00000108510	NM_005121	Mediator complex	[27]
Med13l	ENSG00000123066	NM_015335	Mediator complex	[27]
Med14	ENSG00000180182	NM_004229	Mediator complex	[27]
Med17	ENSG00000042429	NM_004268	Mediator complex	[27]
Med19	ENSG00000156603	NM_153450	Mediator complex	[27]
Med24	ENSG00000008838	NM_001079518	Mediator complex	[27]
Med28	ENSG00000118579	NM_025205	Mediator complex	[27]
Metap2	ENSG00000111142	NM_006838	Nascent protein processor	[28]
Mga	ENSG00000174197	NM_001080541	Transcriptional regulator	[111]
Mll	ENSG00000118058	NM_005933	Trithorax group	[112-114]
Mll2	ENSG00000167548	NM_003482	Trithorax group	[115,116]
Mll3	ENSG00000055609	NM_170606	Trithorax group	[117,118]
Mll5	ENSG00000005483	NM_018682	Trithorax group	[119,120]
Msi1	ENSG00000135097	NM_002442	Neural stem cell marker	[121,122]
Mt1a	ENSG00000205362	NM_005946	NuRD complex component	[123,124]
Mt2a	ENSG00000125148	NM_005953	NuRD complex component	[125]
Mtf2	ENSG00000143033	NM_001164391	Cofactors	[73]
Mthfd1	ENSG00000100714	NM_005956	NADP+ dependent dehydrogenase	[126]
Mybl2	ENSG00000101057	NM_002466	Transcription Regulator	[127]
Myc	ENSG00000136997	NM_002467	Proliferation	[128,129]
Mycn	ENSG00000134323	NM_005378	Transcription regulator	[130]
Nacc1	ENSG00000160877	NM_052876	Transcription regulator	[131]
Nanog	ENSG00000111704	NM_024865	Core pluripotency factor	[132,133]
Nanos1	ENSG00000188613	NM_001009553	Translational repressor	[134]
Ncam1	ENSG00000149294	NM_000615.6	Adhesion	[135]
Ncoa2	NSG00000140396	NM_006540	Steroid and nuclear receptor coactivator	[136]
Ncoa3	ENSG00000124151	NM_006534	Steroid and nuclear receptor	[28]

			coactivator	
Nfrkb	ENSG00000170322	NM_001143835	INO80 complex	[27]
Nodal	ENSG00000156574	NM_018055	Stem Cell Marker/Mesoderm Inducer	[137,138]
Npr1	ENSG00000169418	NM_000906	ESC identity	[139]
Nr0b1	ENSG00000169297	NM_000475	Oct4 Inhibitor	[140,141]
Nr6a1	ENSG00000148200	NM_001489	Trophoblast/ESC maintenance/Proliferation/Neural	[142]
Nts	ENSG00000133636	NM_006183	Neuromodulator	[28]
Onecut2	ENSG00000119547	NM_004852	Transcriptional regulator	[143]
Otx1	ENSG00000115507	NM_001199770	Transcription factor	[144]
Otx2	ENSG00000165588	NM_021728	Transcription factor	[145]
Paf1	ENSG00000006712	NM_019088	Couples Transcription with Histone Modification	[146]
Pcgf6	ENSG00000156374	NM_001011663	Cofactors	[146]
Pcid2	ENSG00000126226	NM_001127202	Proliferation and survival	[28]
Pcna	ENSG00000132646	NM_002592	DNA replication and repair	[147,148]
Phc1	ENSG00000111752	NM_004426	Polycomb group	[149]
Phc2	ENSG00000134686	NM_004427	Polycomb group	[150]
Phc3	ENSG00000173889	NM_024947	Polycomb group	[151]
Pim2	ENSG00000102096	NM_006875	ESC identity	[152]
Podxl	ENSG00000128567	NM_001018111	Adhesion and cell morphology	[1,11,153,154]
Pou5f1	ENSG00000204531	NM_001173531	Core pluripotency factor	[155,156]
Ppp1r3d	ENSG00000132825	NM_006242	Proliferation	[70,157]
Prdm14	ENSG00000147596	NM_024504	ESC identity	[27,158]
Prdm16	ENSG00000142611	NM_022114	ESC identity	[159]
Prdm5	ENSG00000138738	NM_018699	ESC identity	[160]
Prmt6	ENSG00000198890	NM_018137	ESC identity	[161]
Prom1	ENSG00000007062	NM_001145847	Stem cell maintenance	[162]
Ptprz1	ENSG00000106278	NM_002851	Developmental regulator	[163]
Pum1	ENSG00000134644	NM_001020658	Stem cell maintenance	[164]
Pum2	ENSG00000055917	NM_015317	Stem cell maintenance	[164]

Rad21	ENSG00000164754	NM_006265	ESC identity	[165]
Rb1	ENSG00000139687	NM_000321	Proliferation	[13]
Rbbp4	ENSG00000162521	NM_005610	Polycomb group	[166]
Rbbp5	ENSG00000117222	NM_001193272	Polycomb group	[167]
Rbbp7	ENSG00000102054	NM_001198719	Polycomb group	[168]
Rbbp9	ENSG00000089050	NM_006606	Stem cell maintenance	[166]
Rbl2	ENSG00000103479	NM_005611	Transcription regulator	[169]
Rbx1	ENSG00000100387	NM_014248	Ubiquitination	[28]
Rest	ENSG00000084093	NM_001193508.1	Transcription regulator	[170]
Rif1	ENSG00000080345	NM_001177664	DNA repair	[171]
Ring1	ENSG00000204227	NM_002931	Polycomb group	[172,173]
Rnf2	ENSG00000121481	NM_007212	Ubiquitination and epigenetic repression	[28]
Rtf1	ENSG00000137815	NM_015138	Transcription elongation/chromatin remodeling	[174]
Sall1	ENSG00000103449	NM_001127892.1	Transcription regulator	[175]
Sall4	ENSG00000101115	NM_020436	ICM/ESC repressor	[176-179]
Sema4a	ENSG00000196189	NM_001193300	Multifunctional cell signaling	[28]
Setdb1	ENSG00000143379	NM_012432	Silencing of lineage-specific regulators	[180-182]
Setdb2	ENSG00000136169	NM_031915	Silencing of lineage-specific regulators	[1]
Sf3a1	ENSG00000099995	NM_001005409	Spliceosome complex	[27]
Sf3a3	ENSG00000183431	NM_006802	Spliceosome complex	[27]
Sfrp2	ENSG00000145423	NM_003013	Wnt signaling	[183]
Sirt2	ENSG00000068903	NM_001193286	Chromatin regulator	[184]
Skil	ENSG00000136603	NM_005414	Cell division and differentiation	[185]
Smad1	ENSG00000170365	NM_001003688	BMP signaling to core circuitry	[186]
Smad2	ENSG00000175387	NM_001135937	TGF-ß/Activin/Nodal signaling	[137,138,187]
Smad3	ENSG00000166949	NM_001145102	TGF-ß/Activin/Nodal	[137,138,187]

			signaling	
Smarca4	ENSG00000127616	NM_001128849	Chromatin regulator	[188]
Smarca5	ENSG00000153147	NM_003601	Chromatin regulator	[12]
Smarcd1	ENSG00000163104	NM_020159	Chromatin regulator	[189]
Smarcb1	ENSG00000099956	NM_001007468	Chromatin regulator	[190]
Smarcc1	ENSG00000094916	NM_001127321	Chromatin regulator	[191]
Smarcd1	ENSG00000066117	NM_003076	Chromatin regulator	[189]
Smc1a	ENSG00000072501	NM_006306	Chromosome cohesion/DNA repair	[192]
Smo	ENSG00000128602	NM_005631	Hedgehog signaling	[193,194]
Sox2	ENSG00000181449	NM_003106	Core pluripotency factor	[25,144,195]
Sox3	ENSG00000134595	NM_005634	Transcription regulator	[196]
Sp1	ENSG00000185591	NM_001251825	Transcription regulator	[197,198]
Spp1	ENSG00000118785	NM_000582	Transcription regulator	[199]
Stag1	ENSG00000118007	NM_005862	Cohesin complex component/DNA replication	[165]
Stat3	ENSG00000168610	NM_213662	Lif signaling to core circuitry	[200]
Sub1	ENSG00000113387	NM_006713	Spliceosome complex	[27]
Suv39h2	ENSG00000152455	NM_001193424	Chromatin regulator	[201]
Suz12	ENSG00000178691	NM_015355	Polycomb group	[202]
Taf2	ENSG00000064313	NM_003184	TAF complex	[27]
Taf7	ENSG00000178913	NM_005642	TAF complex	[27]
Tcf3	ENSG00000071564	NM_003200	ES Cell Repressor	[203-205]
Tcf7l1	ENSG00000152284	NM_031283	Signaling Transcription factor	[206]
Tcl1a	ENSG00000100721	NM_001098725	Signaling transcription factor	[207]
Tdgf1	ENSG00000163828	NM_003212	ESC marker/Mesoderm Inducer	[208,209]
Terf1	ENSG00000147601	NM_003218	Telomerase inhibitor	[1,209]

Tert	ENSG00000164362	NM_198253	Replication of chromosome termini	[210]
Tgif	ENSG00000177426	NM_003244	Transcriptional regulator	[211]
Thap11	ENSG00000168286	NM_020457.2	Metabolism	[212]
Thy1	ENSG00000154096	NM_006288.3	Cell-cell and cell-ligand interactions	[213]
Tle1	ENSG00000196781	NM_005077	Transcriptional regulator	[214]
Tnfrsf8	ENSG00000120949	NM_001243	Cellular growth	[215]
Top2a	ENSG00000131747	NM_001067	DNA topoisomerase	[12]
Trim16	ENSG00000221926	NM_006470	Tumor suppressor	[28,216]
Trim24	ENSG00000122779	NM_015905	Transcriptional coactivator	[217]
Trim28	ENSG00000130726	NM_005762.2	Self-renewal	[28]
Utf1	ENSG00000171794	NM_003577	Transcriptional regulator	[218,219]
Wdr18	ENSG00000065268	NM_024100	Cofactors	[220]
Wdr5	ENSG00000196363	NM_017588	Trithorax group	[221]
Wnt2b	ENSG00000134245	NM_004185	Wnt signaling	[222]
Wnt8a	ENSG00000061492	NM_058244	Wnt signaling	[223]
Xpo7	ENSG00000130227	NM_001100161	Nuclear export mediator	[28]
Yy1	ENSG00000100811	NM_003403	INO80 complex	[27]
Zfhx3	ENSG00000140836	NM_001164766	Transcription regulator	[224,225]
Zfp41	ENSG00000181638	NM_173832	ESC identity	[70]
Zfp42	ENSG00000179059	NM_174900	Trophoblast/Preimplantation/ES Cell Repressor	[226]
Zfx	ENSG00000005889	NM_003410	Self-renewal	[28]
Zic2	ENSG00000043355	NM_007129	Transcription Regulator	[227]
Zic3	ENSG00000156925	NM_003413	ESC identity	[228]
Zic5	ENSG00000139800	NM_033132	Developmental regulator	[229]
Znf143	ENSG00000166478	NM_003442	ESC identity	[27]
Znf219	ENSG00000165804	NM_001101672	Transcription regulator	[111]
Znf281	ENSG00000162702	NM_012482	Transcription Regulator	[230]
Zscan10	ENSG00000130182	NM_032805	ESC identity	[27]

REFERENCES

1. Skottman H, Mikkola M, Lundin K, Olsson C, Stromberg AM, et al. (2005) Gene expression signatures of seven individual human embryonic stem cell lines. Stem Cells 23: 1343-56.

2. Kosaki R, Gebbia M, Kosaki K, Lewin M, Bowers P, et al. (1999) Left-right axis malformations associated with mutations in ACVR2B, the gene for human activin receptor type IIB. American journal of medical genetics 82: 70-6.

3. Rust W, Balakrishnan T, Zweigerdt R (2009) Cardiomyocyte enrichment from human embryonic stem cell cultures by selection of ALCAM surface expression. Regenerative medicine 4: 225-37.

4. Ohneda O, Ohneda K, Arai F, Lee J, Miyamoto T, et al. (2001) ALCAM (CD166): its role in hematopoietic and endothelial development. Blood 98: 2134-42.

5. Yan Z, Wang Z, Sharova L, Sharov AA, Ling C, et al. (2008) BAF250B-associated SWI/SNF chromatin-remodeling complex is required to maintain undifferentiated mouse embryonic stem cells. Stem Cells 26: 1155-65.

6. Wilson MD, Wang D, Wagner R, Breyssens H, Gertsenstein M, et al. (2008) ARS2 is a conserved eukaryotic gene essential for early mammalian development. Molecular and Cellular Biology 28: 1503-14.

7. Andreu-Agullo C, Maurin T, Thompson CB, Lai EC (2012) Ars2 maintains neural stem-cell identity through direct transcriptional activation of Sox2. Nature 481: 195-8.

8. Rampalli S, Li L, Mak E, Ge K, Brand M, et al. (2007) p38 MAPK signaling regulates recruitment of Ash2L-containing methyltransferase complexes to specific genes during differentiation. Nat Struct Mol Biol 14: 1150-6.

9. ten Berge D, Koole W, Fuerer C, Fish M, Eroglu E, et al. (2008) Wnt signaling mediates self-organization and axis formation in embryoid bodies. Cell Stem Cell 3: 508-18.

10. Ding X, Lin Q, Ensenat-Waser R, Rose-John S, Zenke M (2012) Polycomb group protein Bmi1 promotes hematopoietic cell development from embryonic stem cells. Stem cells and development 21: 121-32.

11. Adewumi O, Aflatoonian B, Ahrlund-Richter L, Amit M, Andrews PW, et al. (2007) Characterization of human embryonic stem cell lines by the International Stem Cell Initiative. Nature Biotechnology 25: 803-16.

12. Assou S, Cerecedo D, Tondeur S, Pantesco V, Hovatta O, et al. (2009) A gene expression signature shared by human mature oocytes and embryonic stem cells. BMC Genomics 10: 10.

13. Kapinas K, Grandy R, Ghule P, Medina R, Becker K, et al. (2012) The Abbreviated Pluripotent Cell Cycle. Journal of cellular physiology.

14. Ruiz S, Panopoulos AD, Herrerias A, Bissig KD, Lutz M, et al. (2011) A high proliferation rate is required for cell reprogramming and maintenance of human embryonic stem cell identity. Curr Biol 21: 45-52.

15. Tetzlaff MT, Bai C, Finegold M, Wilson J, Harper JW, et al. (2004) Cyclin F disruption compromises placental development and affects normal cell cycle execution. Molecular and Cellular Biology 24: 2487-98.

16. Jiang W, Sui X, Zhang D, Liu M, Ding M, et al. (2011) CD24: a novel surface marker for PDX1-positive pancreatic progenitors derived from human embryonic stem cells. Stem Cells 29: 609-17.

17. Haegel H, Dierich A, Ceredig R (1994) CD44 in differentiated embryonic stem cells: surface expression and transcripts encoding multiple variants. Dev Immunol 3: 239-46.

18. Kolle G, Ho M, Zhou Q, Chy HS, Krishnan K, et al. (2009) Identification of human embryonic stem cell surface markers by combined membrane-polysome translation state array analysis and immunotranscriptional profiling. Stem Cells 27: 2446-56.

19. Menchon C, Edel MJ, Izpisua Belmonte JC (2011) The cell cycle inhibitor p27Kip(1) controls self-renewal and pluripotency of human embryonic stem cells by regulating the cell cycle, Brachyury and Twist. Cell Cycle 10: 1435-47.

20. Caron C, Pivot-Pajot C, van Grunsven LA, Col E, Lestrat C, et al. (2003) Cdyl: a new transcriptional co-repressor. EMBO Rep 4: 877-82.

21. Gaspar-Maia A, Alajem A, Polesso F, Sridharan R, Mason MJ, et al. (2009) Chd1 regulates open chromatin and pluripotency of embryonic stem cells. Nature 460: 863-8.

22. Bajpai R, Chen DA, Rada-Iglesias A, Zhang J, Xiong Y, et al. (2010) CHD7 cooperates with PBAF to control multipotent neural crest formation. Nature 463: 958-62.

23. Schnetz MP, Handoko L, Akhtar-Zaidi B, Bartels CF, Pereira CF, et al. (2010) CHD7 targets active gene enhancer elements to modulate ES cell-specific gene expression. PLoS Genet 6: e1001023.

24. Shaughnessy J (2005) Amplification and overexpression of CKS1B at chromosome band 1q21 is associated with reduced levels of p27Kip1 and an aggressive clinical course in multiple myeloma. Hematology 10 Suppl 1: 117-26.

25. Richards M, Tan SP, Tan JH, Chan WK, Bongso A (2004) The transcriptome profile of human embryonic stem cells as defined by SAGE. Stem Cells 22: 51-64.

26. Zheng X, Dumitru R, Lackford BL, Freudenberg JM, Singh AP, et al. (2012) Cnot1, Cnot2, and Cnot3 Maintain Mouse and Human ESC Identity and Inhibit Extraembryonic Differentiation. Stem Cells 30: 910-22.

27. Chia N-Y, Chan Y-S, Feng B, Lu X, Orlov YL, et al. (2010) A genome-wide RNAi screen reveals determinants of human embryonic stem cell identity. Nature 468: 316-20.

28. Hu G, Kim J, Xu Q, Leng Y, Orkin SH, et al. (2009) A genome-wide RNAi screen identifies a new transcriptional module required for self-renewal. Genes & Development 23: 837-48.

29. Lane MA, Xu J, Wilen EW, Sylvester R, Derguini F, et al. (2008) LIF removal increases CRABPI and CRABPII transcripts in embryonic stem cells cultured in retinol or 4-oxoretinol. Mol Cell Endocrinol 280: 63-74.

30. Maldonado-Saldivia J, van den Bergen J, Krouskos M, Gilchrist M, Lee C, et al. (2007) Dppa2 and Dppa4 are closely linked SAP motif genes restricted to pluripotent cells and the germ line. Stem Cells 25: 19-28.

31. Bortvin A, Goodheart M, Liao M, Page DC (2004) Dppa3 / Pgc7 / stella is a maternal factor and is not required for germ cell specification in mice. BMC developmental biology 4: 2.

32. Bowles J, Teasdale RP, James K, Koopman P (2003) Dppa3 is a marker of pluripotency and has a human homologue that is expressed in germ cell tumours. Cytogenet Genome Res 101: 261-5.

33. Kim SK, Suh MR, Yoon HS, Lee JB, Oh SK, et al. (2005) Identification of developmental pluripotency associated 5 expression in human pluripotent stem cells. Stem Cells 23: 458-62.

34. Takahashi YH, Westfield GH, Oleskie AN, Trievel RC, Shilatifard A, et al. (2011) Structural analysis of the core COMPASS family of histone H3K4 methylases from yeast to human. Proceedings of the National Academy of Sciences of the United States of America 108: 20526-31.

35. Jiang H, Shukla A, Wang X, Chen WY, Bernstein BE, et al. (2011) Role for Dpy-30 in ES cell-fate specification by regulation of H3K4 methylation within bivalent domains. Cell 144: 513-25.

36. Yeo HC, Beh TT, Quek JJ, Koh G, Chan KK, et al. (2011) Integrated transcriptome and binding sites analysis implicates E2F in the regulation of self-renewal in human pluripotent stem cells. PLoS One 6: e27231.

37. Boyer LA, Plath K, Zeitlinger J, Brambrink T, Medeiros LA, et al. (2006) Polycomb complexes repress developmental regulators in murine embryonic stem cells. Nature 441: 349-53.

38. Feldman N, Gerson A, Fang J, Li E, Zhang Y, et al. (2006) G9a-mediated irreversible epigenetic inactivation of Oct-3/4 during early embryogenesis. Nature Cell Biology 8: 188-94.

39. Takahashi K, Mitsui K, Yamanaka S (2003) Role of ERas in promoting tumour-like properties in mouse embryonic stem cells. Nature 423: 541-5.

40. Zhang X, Zhang J, Wang T, Esteban MA, Pei D (2008) Esrrb activates Oct4 transcription and sustains self-renewal and pluripotency in embryonic stem cells. The Journal of biological chemistry 283: 35825-33.

41. Feng B, Jiang J, Kraus P, Ng JH, Heng JC, et al. (2009) Reprogramming of fibroblasts into induced pluripotent stem cells with orphan nuclear receptor Esrrb. Nature Cell Biology 11: 197-203.

42. Moller E, Stenman G, Mandahl N, Hamberg H, Molne L, et al. (2008) POU5F1, encoding a key regulator of stem cell pluripotency, is fused to EWSR1 in hidradenoma of the skin and mucoepidermoid carcinoma of the salivary glands. J Pathol 215: 78-86.

43. Shen X, Liu Y, Hsu YJ, Fujiwara Y, Kim J, et al. (2008) EZH1 mediates methylation on histone H3 lysine 27 and complements EZH2 in maintaining stem cell identity and executing pluripotency. Mol Cell 32: 491-502.

44. Shin DM, Liu R, Wu W, Waigel SJ, Zacharias W, et al. (2012) Global Gene Expression Analysis of Very Small Embryonic-Like Stem Cells Reveals that the Ezh2-Dependent Bivalent Domain Mechanism Contributes to Their Pluripotent State. Stem cells and development. 21(10):1639-52.

45. Okita K, Ichisaka T, Yamanaka S (2007) Generation of germline-competent induced pluripotent stem cells. Nature 448: 313-7.

46. Nishimoto S, Nishida E (2007) Fibroblast growth factor 13 is essential for neural differentiation in Xenopus early embryonic development. The Journal of biological chemistry 282: 24255-61.

47. Kunath T, Saba-El-Leil MK, Almousailleakh M, Wray J, Meloche S, et al. (2007) FGF stimulation of the Erk1/2 signalling cascade triggers transition of pluripotent embryonic stem cells from self-renewal to lineage commitment. Development 134: 2895-902.

48. Rappold I, Ziegler BL, Kohler I, Marchetto S, Rosnet O, et al. (1997) Functional and phenotypic characterization of cord blood and bone marrow subsets expressing FLT3 (CD135) receptor tyrosine kinase. Blood 90: 111-25.

49. Pan G, Li J, Zhou Y, Zheng H, Pei D (2006) A negative feedback loop of transcription factors that controls stem cell pluripotency and self-renewal. FASEB journal : official publication of the Federation of American Societies for Experimental Biology 20: 1730-2.

50. Yoon SJ, Wills AE, Chuong E, Gupta R, Baker JC (2011) HEB and E2A function as SMAD/FOXH1 cofactors. Genes & Development 25: 1654-61.

51. Kudo T, Kaneko M, Iwasaki H, Togayachi A, Nishihara S, et al. (2004) Normal embryonic and germ cell development in mice lacking alpha 1,3-fucosyltransferase IX (Fut9) which show disappearance of stage-specific embryonic antigen 1. Molecular and Cellular Biology 24: 4221-8.

52. Adewumi O, Aflatoonian B, Ahrlund-Richter L, Amit M, Andrews PW, et al. (2007) Characterization of human embryonic stem cell lines by the International Stem Cell Initiative. Nature Biotechnology 25: 803-16.

53. Chapman G, Remiszewski JL, Webb GC, Schulz TC, Bottema CD, et al. (1997) The mouse homeobox gene, Gbx2: genomic organization and expression in pluripotent cells in vitro and in vivo. Genomics 46: 223-33.

54. Clark AT, Rodriguez RT, Bodnar MS, Abeyta MJ, Cedars MI, et al. (2004) Human STELLAR, NANOG, and GDF3 genes are expressed in pluripotent cells and map to chromosome 12p13, a hotspot for teratocarcinoma. Stem Cells 22: 169-79.

55. Player A, Wang Y, Bhattacharya B, Rao M, Puri RK, et al. (2006) Comparisons between transcriptional regulation and RNA expression in human embryonic stem cell lines. Stem cells and development 15: 315-23.

56. Wu SM, Choo AB, Yap MG, Chan KK (2010) Role of Sonic hedgehog signaling and the expression of its components in human embryonic stem cells. Stem Cell Res 4: 38-49.

57. Maekawa M, Yamaguchi K, Nakamura T, Shibukawa R, Kodanaka I, et al. (2011) Direct reprogramming of somatic cells is promoted by maternal transcription factor Glis1. Nature 474: 225-9.

58. Beekman C, Nichane M, De Clercq S, Maetens M, Floss T, et al. (2006) Evolutionarily conserved role of nucleostemin: controlling proliferation of stem/progenitor cells during early vertebrate development. Molecular and Cellular Biology 26: 9291-301.

59. Tsai NP, Bi J, Wei LN (2007) The adaptor Grb7 links netrin-1 signaling to regulation of mRNA translation. Embo J 26: 1522-31.

60. Thommes K, Lennartsson J, Carlberg M, Ronnstrand L (1999) Identification of Tyr-703 and Tyr-936 as the primary association sites for Grb2 and Grb7 in the c-Kit/stem cell factor receptor. Biochem J 341 (Pt 1): 211-6.

61. Vassena R, Boue S, Gonzalez-Roca E, Aran B, Auer H, et al. (2011) Waves of early transcriptional activation and pluripotency program initiation during human preimplantation development. Development 138: 3699-709.

62. Choudhary M, Zhang X, Stojkovic P, Hyslop L, Anyfantis G, et al. (2007) Putative role of hyaluronan and its related genes, HAS2 and RHAMM, in human early preimplantation embryogenesis and embryonic stem cell characterization. Stem Cells 25: 3045-57.

63. Dejosez M, Levine SS, Frampton GM, Whyte WA, Stratton SA, et al. (2010) Ronin/Hcf-1 binds to a hyperconserved enhancer element and regulates genes involved in the growth of embryonic stem cells. Genes & Development 24: 1479-84.

64. Palmer RD, Barbosa-Morais NL, Gooding EL, Muralidhar B, Thornton CM, et al. (2008) Pediatric malignant germ cell tumors show characteristic transcriptome profiles. Cancer Res 68: 4239-47.

65. Webb GC, Thomas PQ, Ford JH, Rathjen PD (1993) Hesx1, a homeobox gene expressed by murine embryonic stem cells, maps to mouse chromosome 14, bands A3-B. Genomics 18: 464-6.

66. Held T, Barakat AZ, Mohamed BA, Paprotta I, Meinhardt A, et al. (2011) Heat-shock protein HSPA4 is required for progression of spermatogenesis. Reproduction 142: 133-44.

67. Arrigo AP (2005) In search of the molecular mechanism by which small stress proteins counteract apoptosis during cellular differentiation. J Cell Biochem 94: 241-6.

68. Sekkai D, Gruel G, Herry M, Moucadel V, Constantinescu SN, et al. (2005) Microarray analysis of LIF/Stat3 transcriptional targets in embryonic stem cells. Stem Cells 23: 1634-42.

69. Zhu H, Shah S, Shyh-Chang N, Shinoda G, Einhorn WS, et al. (2010) Lin28a transgenic mice manifest size and puberty phenotypes identified in human genetic association studies. Nature Genetics 42: 626-30.

70. Tang F, Barbacioru C, Bao S, Lee C, Nordman E, et al. (2010) Tracing the derivation of embryonic stem cells from the inner cell mass by single-cell RNA-Seq analysis. Cell Stem Cell 6: 468-78.

71. Cariati M, Naderi A, Brown JP, Smalley MJ, Pinder SE, et al. (2008) Alpha-6 integrin is necessary for the tumourigenicity of a stem cell-like subpopulation within the MCF7 breast cancer cell line. Int J Cancer 122: 298-304.

72. Buageaw A, Sukhwani M, Ben-Yehudah A, Ehmcke J, Rawe VY, et al. (2005) GDNF family receptor alpha1 phenotype of spermatogonial stem cells in immature mouse testes. Biology of Reproduction 73: 1011-6.

73. Zhang Z, Jones A, Sun CW, Li C, Chang CW, et al. (2011) PRC2 complexes with JARID2, MTF2, and esPRC2p48 in ES cells to modulate ES cell pluripotency and somatic cell reprogramming. Stem Cells 29: 229-40.

74. Candau R, Moore PA, Wang L, Barlev N, Ying CY, et al. (1996) Identification of human proteins functionally conserved with the yeast putative adaptors ADA2 and GCN5. Molecular and Cellular Biology 16: 593-602.

75. Herrera JE, Bergel M, Yang XJ, Nakatani Y, Bustin M (1997) The histone acetyltransferase activity of human GCN5 and PCAF is stabilized by coenzymes. The Journal of biological chemistry 272: 27253-8.

76. Putnik J, Zhang CD, Archangelo LF, Tizazu B, Bartels S, et al. (2007) The interaction of ETV6 (TEL) and TIP60 requires a functional histone acetyltransferase domain in TIP60. Biochim Biophys Acta 1772: 1211-24.

77. Fazzio TG, Huff JT, Panning B (2008) An RNAi screen of chromatin proteins identifies Tip60-p400 as a regulator of embryonic stem cell identity. Cell 134: 162-74.

78. Pelletier N, Champagne N, Stifani S, Yang XJ (2002) MOZ and MORF histone acetyltransferases interact with the Runt-domain transcription factor Runx2. Oncogene 21: 2729-40.

79. Yang XJ, Ullah M (2007) MOZ and MORF, two large MYSTic HATs in normal and cancer stem cells. Oncogene 26: 5408-19.

80. Adamo A, Sese B, Boue S, Castano J, Paramonov I, et al. (2011) LSD1 regulates the balance between self-renewal and differentiation in human embryonic stem cells. Nature Cell Biology 13: 652-9.

81. Ko SY, Kang HY, Lee HS, Han SY, Hong SH (2006) Identification of Jmjd1a as a STAT3 downstream gene in mES cells. Cell Struct Funct 31: 53-62.

82. Loh YH, Zhang W, Chen X, George J, Ng HH (2007) Jmjd1a and Jmjd2c histone H3 Lys 9 demethylases regulate self-renewal in embryonic stem cells. Genes & Development 21: 2545-57.

83. Katoh Y, Katoh M (2007) Comparative integromics on JMJD2A, JMJD2B and JMJD2C: preferential expression of JMJD2C in undifferentiated ES cells. Int J Mol Med 20: 269-73.

84. Dey BK, Stalker L, Schnerch A, Bhatia M, Taylor-Papidimitriou J, et al. (2008) The histone demethylase KDM5b/JARID1b plays a role in cell fate decisions by blocking terminal differentiation. Molecular and Cellular Biology 28: 5312-27.

85. Xie L, Pelz C, Wang W, Bashar A, Varlamova O, et al. (2011) KDM5B regulates embryonic stem cell self-renewal and represses cryptic intragenic transcription. Embo J 30: 1473-84.

86. Nadin BM, Goodell MA, Hirschi KK (2003) Phenotype and hematopoietic potential of side population cells throughout embryonic development. Blood 102: 2436-43.

87. Feng H, Sandlow JI, Sandra A (1997) Expression and function of the c-kit proto-oncogene protein in mouse sperm. Biology of Reproduction 57: 194-203.

88. Geissler EN, Liao M, Brook JD, Martin FH, Zsebo KM, et al. (1991) Stem cell factor (SCF), a novel hematopoietic growth factor and ligand for c-kit tyrosine kinase receptor, maps on human chromosome 12 between 12q14.3 and 12qter. Somat Cell Mol Genet 17: 207-14.

89. Roth C, Schuierer M, Gunther K, Buettner R (2000) Genomic structure and DNA binding properties of the human zinc finger transcriptional repressor AP-2rep (KLF12). Genomics 63: 384-90.

90. Jiang J, Chan YS, Loh YH, Cai J, Tong GQ, et al. (2008) A core Klf circuitry regulates self-renewal of embryonic stem cells. Nature Cell Biology 10: 353-60.

91. Hall J, Guo G, Wray J, Eyres I, Nichols J, et al. (2009) Oct4 and LIF/Stat3 additively induce Kruppel factors to sustain embryonic stem cell self-renewal. Cell Stem Cell 5: 597-609.

92. Ema M, Mori D, Niwa H, Hasegawa Y, Yamanaka Y, et al. (2008) Kruppel-like factor 5 is essential for blastocyst development and the normal self-renewal of mouse ESCs. Cell Stem Cell 3: 555-67.

93. Lin SC, Wani MA, Whitsett JA, Wells JM (2010) Klf5 regulates lineage formation in the pre-implantation mouse embryo. Development 137: 3953-63.

94. Wong RC, Ibrahim A, Fong H, Thompson N, Lock LF, et al. (2011) L1TD1 is a marker for undifferentiated human embryonic stem cells. PLoS One 6: e19355.

95. Narva E, Rahkonen N, Emani MR, Lund R, Pursiheimo JP, et al. (2012) RNA-binding protein L1TD1 interacts with LIN28 via RNA and is required for human embryonic stem cell self-renewal and cancer cell proliferation. Stem Cells 30: 452-60.

96. Mesnard D, Guzman-Ayala M, Constam DB (2006) Nodal specifies embryonic visceral endoderm and sustains pluripotent cells in the epiblast before overt axial patterning. Development 133: 2497-505.

97. Barroso-delJesus A, Lucena-Aguilar G, Sanchez L, Ligero G, Gutierrez-Aranda I, et al. (2011) The Nodal inhibitor Lefty is negatively modulated by the microRNA miR-302 in human embryonic stem cells. FASEB journal : official publication of the Federation of American Societies for Experimental Biology 25: 1497-508.

98. Smith JR, Vallier L, Lupo G, Alexander M, Harris WA, et al. (2008) Inhibition of Activin/Nodal signaling promotes specification of human embryonic stem cells into neuroectoderm. Developmental biology 313: 107-17.

99. Yu J, Vodyanik MA, Smuga-Otto K, Antosiewicz-Bourget J, Frane JL, et al. (2007) Induced pluripotent stem cell lines derived from human somatic cells. Science 318: 1917-20.

100. Lu L, Katsaros D, Shaverdashvili K, Qian B, Wu Y, et al. (2009) Pluripotent factor lin-28 and its homologue lin-28b in epithelial ovarian cancer and their associations with disease outcomes and expression of let-7a and IGF-II. Eur J Cancer 45: 2212-8.

101. Cotterman R, Knoepfler PS (2009) N-Myc regulates expression of pluripotency genes in neuroblastoma including lif, klf2, klf4, and lin28b. PLoS One 4: e5799.

102. Bresson-Mazet C, Gandrillon O, Gonin-Giraud S (2008) Stem cell antigen 2: a new gene involved in the self-renewal of erythroid progenitors. Cell proliferation 41: 726-38.

103. Blackwood EM, Luscher B, Eisenman RN (1992) Myc and Max associate in vivo. Genes & Development 6: 71-80.

104. Amati B, Brooks MW, Levy N, Littlewood TD, Evan GI, et al. (1993) Oncogenic activity of the c-Myc protein requires dimerization with Max. Cell 72: 233-45.

105. Maslov AY, Bailey KJ, Mielnicki LM, Freeland AL, Sun X, et al. (2007) Stem/progenitor cell-specific enhanced green fluorescent protein expression driven by the endogenous Mcm2 promoter. Stem Cells 25: 132-8.

106. Lin W, Zhang J, Liu X, Fei Z, Li X, et al. (2009) RNAi-mediated inhibition of MSP58 decreases tumour growth, migration and invasion in a human glioma cell line. J Cell Mol Med 13: 4608-22.

107. Sumitomo A, Ishino R, Urahama N, Inoue K, Yonezawa K, et al. (2010) The transcriptional mediator subunit MED1/TRAP220 in stromal cells is involved in hematopoietic stem/progenitor cell support through osteopontin expression. Molecular and Cellular Biology 30: 4818-27.

108. Kagey MH, Newman JJ, Bilodeau S, Zhan Y, Orlando DA, et al. (2010) Mediator and cohesin connect gene expression and chromatin architecture. Nature 467: 430-5.

109. Lin X, Rinaldo L, Fazly AF, Xu X (2007) Depletion of Med10 enhances Wnt and suppresses Nodal signaling during zebrafish embryogenesis. Developmental biology 303: 536-48.

110. Tutter AV, Kowalski MP, Baltus GA, Iourgenko V, Labow M, et al. (2009) Role for Med12 in regulation of Nanog and Nanog target genes. The Journal of biological chemistry 284: 3709-18.

111. van den Berg DLC, Snoek T, Mullin NP, Yates A, Bezstarosti K, et al. (2010) An Oct4-Centered Protein Interaction Network in Embryonic Stem Cells. Cell Stem Cell 6: 369-81.

112. Cao F, Chen Y, Cierpicki T, Liu Y, Basrur V, et al. (2010) An Ash2L/RbBP5 heterodimer stimulates the MLL1 methyltransferase activity through coordinated substrate interactions with the MLL1 SET domain. PLoS One 5: e14102.

113. Wysocka J, Swigut T, Milne TA, Dou Y, Zhang X, et al. (2005) WDR5 associates with histone H3 methylated at K4 and is essential for H3 K4 methylation and vertebrate development. Cell 121: 859-72.

114. Dou Y, Milne TA, Tackett AJ, Smith ER, Fukuda A, et al. (2005) Physical association and coordinate function of the H3 K4 methyltransferase MLL1 and the H4 K16 acetyltransferase MOF. Cell 121: 873-85.

115. Lubitz S, Glaser S, Schaft J, Stewart AF, Anastassiadis K (2007) Increased apoptosis and skewed differentiation in mouse embryonic stem cells lacking the histone methyltransferase Mll2. Mol Biol Cell 18: 2356-66.

116. Huntsman DG, Chin SF, Muleris M, Batley SJ, Collins VP, et al. (1999) MLL2, the second human homolog of the Drosophila trithorax gene, maps to 19q13.1 and is amplified in solid tumor cell lines. Oncogene 18: 7975-84.

117. Ruault M, Brun ME, Ventura M, Roizes G, De Sario A (2002) MLL3, a new human member of the TRX/MLL gene family, maps to 7q36, a chromosome region frequently deleted in myeloid leukaemia. Gene 284: 73-81.

118. Chauhan C, Zraly CB, Parilla M, Diaz MO, Dingwall AK (2012) Histone recognition and nuclear receptor co-activator functions of Drosophila Cara Mitad, a homolog of the N-terminal portion of mammalian MLL2 and MLL3. Development 139: 1997-2008.

119. Emerling BM, Bonifas J, Kratz CP, Donovan S, Taylor BR, et al. (2002) MLL5, a homolog of Drosophila trithorax located within a segment of chromosome band 7q22 implicated in myeloid leukemia. Oncogene 21: 4849-54.

120. Deng LW, Chiu I, Strominger JL (2004) MLL 5 protein forms intranuclear foci, and overexpression inhibits cell cycle progression. Proceedings of the National Academy of Sciences of the United States of America 101: 757-62.

121. Ye F, Zhou C, Cheng Q, Shen J, Chen H (2008) Stem-cell-abundant proteins Nanog, Nucleostemin and Musashi1 are highly expressed in malignant cervical epithelial cells. BMC Cancer 8: 108.

122. Kawahara H, Okada Y, Imai T, Iwanami A, Mischel PS, et al. (2011) Musashi1 cooperates in abnormal cell lineage protein 28 (Lin28)-mediated let-7 family microRNA biogenesis in early neural differentiation. The Journal of biological chemistry 286: 16121-30.

123. Liang J, Wan M, Zhang Y, Gu P, Xin H, et al. (2008) Nanog and Oct4 associate with unique transcriptional repression complexes in embryonic stem cells. Nature Cell Biology 10: 731-9.

124. Ghanta KS, Li DQ, Eswaran J, Kumar R (2011) Gene profiling of MTA1 identifies novel gene targets and functions. PLoS One 6: e17135.

125. Foygel K, Choi B, Jun S, Leong DE, Lee A, et al. (2008) A novel and critical role for Oct4 as a regulator of the maternal-embryonic transition. PLoS One 3: e4109.

126. Krajinovic M, Lemieux-Blanchard E, Chiasson S, Primeau M, Costea I, et al. (2004) Role of polymorphisms in MTHFR and MTHFD1 genes in the outcome of childhood acute lymphoblastic leukemia. Pharmacogenomics J 4: 66-72.

127. Lorvellec M, Dumon S, Maya-Mendoza A, Jackson D, Frampton J, et al. (2010) B-Myb is critical for proper DNA duplication during an unperturbed S phase in mouse embryonic stem cells. Stem Cells 28: 1751-9.

128. Cartwright P, McLean C, Sheppard A, Rivett D, Jones K, et al. (2005) LIF/STAT3 controls ES cell self-renewal and pluripotency by a Myc-dependent mechanism. Development 132: 885-96.

129. Takahashi K, Yamanaka S (2006) Induction of pluripotent stem cells from mouse embryonic and adult fibroblast cultures by defined factors. Cell 126: 663-76.

130. Varlakhanova NV, Cotterman RF, deVries WN, Morgan J, Donahue LR, et al. (2010) myc maintains embryonic stem cell pluripotency and self-renewal. Differentiation 80: 9-19.

131. Jinawath N, Vasoontara C, Yap KL, Thiaville MM, Nakayama K, et al. (2009) NAC-1, a potential stem cell pluripotency factor, contributes to paclitaxel resistance in ovarian cancer through inactivating Gadd45 pathway. Oncogene 28: 1941-8.

132. Mitsui K, Tokuzawa Y, Itoh H, Segawa K, Murakami M, et al. (2003) The homeoprotein Nanog is required for maintenance of pluripotency in mouse epiblast and ES cells. Cell 113: 631-42.

133. Chambers I, Colby D, Robertson M, Nichols J, Lee S, et al. (2003) Functional expression cloning of Nanog, a pluripotency sustaining factor in embryonic stem cells. Cell 113: 643-55.

134. Koprunner M, Thisse C, Thisse B, Raz E (2001) A zebrafish nanos-related gene is essential for the development of primordial germ cells. Genes & Development 15: 2877-85.

135. Young HE, Steele TA, Bray RA, Detmer K, Blake LW, et al. (1999) Human pluripotent and progenitor cells display cell surface cluster differentiation markers CD10, CD13, CD56, and MHC class-I. Proc Soc Exp Biol Med 221: 63-71.

136. Voegel JJ, Heine MJ, Tini M, Vivat V, Chambon P, et al. (1998) The coactivator TIF2 contains three nuclear receptor-binding motifs and mediates transactivation through CBP binding-dependent and -independent pathways. Embo J 17: 507-19.

137. James D, Levine AJ, Besser D, Hemmati-Brivanlou A (2005) TGFbeta/activin/nodal signaling is necessary for the maintenance of pluripotency in human embryonic stem cells. Development 132: 1273-82.

138. Vallier L, Alexander M, Pedersen RA (2005) Activin/Nodal and FGF pathways cooperate to maintain pluripotency of human embryonic stem cells. J Cell Sci 118: 4495-509.

139. Abdelalim EM, Tooyama I (2011) NPR-A regulates self-renewal and pluripotency of embryonic stem cells. Cell Death Dis 2: e127.

140. Niakan KK, Davis EC, Clipsham RC, Jiang M, Dehart DB, et al. (2006) Novel role for the orphan nuclear receptor Dax1 in embryogenesis, different from steroidogenesis. Mol Genet Metab 88: 261-71.

141. Sun C, Nakatake Y, Akagi T, Ura H, Matsuda T, et al. (2009) Dax1 binds to Oct3/4 and inhibits its transcriptional activity in embryonic stem cells. Molecular and Cellular Biology 29: 4574-83.

142. Gu P, LeMenuet D, Chung AC, Mancini M, Wheeler DA, et al. (2005) Orphan nuclear receptor GCNF is required for the repression of pluripotency genes during retinoic acid-induced embryonic stem cell differentiation. Molecular and Cellular Biology 25: 8507-19.

143. Clotman F, Jacquemin P, Plumb-Rudewiez N, Pierreux CE, Van der Smissen P, et al. (2005) Control of liver cell fate decision by a gradient of TGF beta signaling modulated by Onecut transcription factors. Genes & Development 19: 1849-54.

144. Boyer LA, Lee TI, Cole MF, Johnstone SE, Levine SS, et al. (2005) Core transcriptional regulatory circuitry in human embryonic stem cells. Cell 122: 947-56.

145. Acampora D, Avantaggiato V, Tuorto F, Barone P, Perera M, et al. (1999) Differential transcriptional control as the major molecular event in generating Otx1-/- and Otx2-/- divergent phenotypes. Development 126: 1417-26.

146. Ding L, Paszkowski-Rogacz M, Nitzsche A, Slabicki MM, Heninger AK, et al. (2009) A genome-scale RNAi screen for Oct4 modulators defines a role of the Paf1 complex for embryonic stem cell identity. Cell Stem Cell 4: 403-15.

147. Chen IT, Smith ML, O'Connor PM, Fornace AJ, Jr. (1995) Direct interaction of Gadd45 with PCNA and evidence for competitive interaction of Gadd45 and p21Waf1/Cip1 with PCNA. Oncogene 11: 1931-7.

148. Smith ML, Chen IT, Zhan Q, Bae I, Chen CY, et al. (1994) Interaction of the p53-regulated protein Gadd45 with proliferating cell nuclear antigen. Science 266: 1376-80.

149. Sun Y, Li H, Liu Y, Mattson MP, Rao MS, et al. (2008) Evolutionarily conserved transcriptional co-expression guiding embryonic stem cell differentiation. PLoS One 3: e3406.

150. Isono K, Fujimura Y, Shinga J, Yamaki M, J OW, et al. (2005) Mammalian polyhomeotic homologues Phc2 and Phc1 act in synergy to mediate polycomb repression of Hox genes. Molecular and Cellular Biology 25: 6694-706.

151. Deshpande AM, Akunowicz JD, Reveles XT, Patel BB, Saria EA, et al. (2007) PHC3, a component of the hPRC-H complex, associates with E2F6 during G0 and is lost in osteosarcoma tumors. Oncogene 26: 1714-22.

152. Agrawal S, Koschmieder S, Baumer N, Reddy NG, Berdel WE, et al. (2008) Pim2 complements Flt3 wild-type receptor in hematopoietic progenitor cell transformation. Leukemia : official journal of the Leukemia Society of America, Leukemia Research Fund, U.K 22: 78-86.

153. Doyonnas R, Kershaw DB, Duhme C, Merkens H, Chelliah S, et al. (2001) Anuria, omphalocele, and perinatal lethality in mice lacking the CD34-related protein podocalyxin. The Journal of experimental medicine 194: 13-27.

154. Narsinh KH, Sun N, Sanchez-Freire V, Lee AS, Almeida P, et al. (2011) Single cell transcriptional profiling reveals heterogeneity of human induced pluripotent stem cells. The Journal of clinical investigation 121: 1217-21.

155. Nichols J, Zevnik B, Anastassiadis K, Niwa H, Klewe-Nebenius D, et al. (1998) Formation of pluripotent stem cells in the mammalian embryo depends on the POU transcription factor Oct4. Cell 95: 379-91.

156. Scholer HR, Hatzopoulos AK, Balling R, Suzuki N, Gruss P (1989) A family of octamer-specific proteins present during mouse embryogenesis: evidence for germline-specific expression of an Oct factor. Embo J 8: 2543-50.

157. Armstrong CG, Browne GJ, Cohen P, Cohen PT (1997) PPP1R6, a novel member of the family of glycogen-targetting subunits of protein phosphatase 1. FEBS Lett 418: 210-4.

158. Yamaji M, Seki Y, Kurimoto K, Yabuta Y, Yuasa M, et al. (2008) Critical function of Prdm14 for the establishment of the germ cell lineage in mice. Nature Genetics 40: 1016-22.

159. Chuikov S, Levi BP, Smith ML, Morrison SJ (2010) Prdm16 promotes stem cell maintenance in multiple tissues, partly by regulating oxidative stress. Nature Cell Biology 12: 999-1006.

160. Deng Q, Huang S (2004) PRDM5 is silenced in human cancers and has growth suppressive activities. Oncogene 23: 4903-10.

161. Lee YH, Ma H, Tan TZ, Ng SS, Soong R, et al. (2012) Protein Arginine Methyltransferase 6 Regulates Embryonic Stem Cell Identity. Stem cells and development. 21(14):2613-22.

162. Jozefczuk J, Stachelscheid H, Chavez L, Herwig R, Lehrach H, et al. (2010) Molecular characterization of cultured adult human liver progenitor cells. Tissue engineering. Part C, Methods 16: 821-34.

163. Lamprianou S, Chatzopoulou E, Thomas JL, Bouyain S, Harroch S (2011) A complex between contactin-1 and the protein tyrosine phosphatase PTPRZ controls the development of oligodendrocyte precursor cells. Proceedings of the National Academy of Sciences of the United States of America 108: 17498-503.

164. Spassov DS, Jurecic R (2003) Mouse Pum1 and Pum2 genes, members of the Pumilio family of RNA-binding proteins, show differential expression in fetal and adult hematopoietic stem cells and progenitors. Blood Cells Mol Dis 30: 55-69.

165. Nitzsche A, Paszkowski-Rogacz M, Matarese F, Janssen-Megens EM, Hubner NC, et al. (2011) RAD21 cooperates with pluripotency transcription factors in the maintenance of embryonic stem cell identity. PLoS One 6: e19470.

166. O'Connor MD, Wederell E, Robertson G, Delaney A, Morozova O, et al. (2011) Retinoblastoma-binding proteins 4 and 9 are important for human pluripotent stem cell maintenance. Exp Hematol 39: 866-79 e1.

167. Avdic V, Zhang P, Lanouette S, Voronova A, Skerjanc I, et al. (2011) Fine-tuning the stimulation of MLL1 methyltransferase activity by a histone H3-based peptide mimetic. FASEB journal : official publication of the Federation of American Societies for Experimental Biology 25: 960-7.

168. Yang J, Kiefer S, Rauchman M (2002) Characterization of the gene encoding mouse retinoblastoma binding protein-7, a component of chromatin-remodeling complexes. Genomics 80: 407-15.

169. Claudio PP, Howard CM, Baldi A, De Luca A, Fu Y, et al. (1994) p130/pRb2 has growth suppressive properties similar to yet distinctive from those of retinoblastoma family members pRb and p107. Cancer Res 54: 5556-60.

170. Singh SK, Kagalwala MN, Parker-Thornburg J, Adams H, Majumder S (2008) REST maintains self-renewal and pluripotency of embryonic stem cells. Nature 453: 223-7.

171. Adams IR, McLaren A (2004) Identification and characterisation of mRif1: a mouse telomere-associated protein highly expressed in germ cells and embryo-derived pluripotent stem cells. Developmental dynamics : an official publication of the American Association of Anatomists 229: 733-44.

172. Stock JK, Giadrossi S, Casanova M, Brookes E, Vidal M, et al. (2007) Ring1-mediated ubiquitination of H2A restrains poised RNA polymerase II at bivalent genes in mouse ES cells. Nature Cell Biology 9: 1428-35.

173. Endoh M, Endo TA, Endoh T, Fujimura Y, Ohara O, et al. (2008) Polycomb group proteins Ring1A/B are functionally linked to the core transcriptional regulatory circuitry to maintain ES cell identity. Development 135: 1513-24.

174. Mueller CL, Jaehning JA (2002) Ctr9, Rtf1, and Leo1 are components of the Paf1/RNA polymerase II complex. Molecular and Cellular Biology 22: 1971-80.

175. Karantzali E, Lekakis V, Ioannou M, Hadjimichael C, Papamatheakis J, et al. (2011) Sall1 regulates embryonic stem cell differentiation in association with nanog. The Journal of biological chemistry 286: 1037-45.

176. Zhang J, Tam WL, Tong GQ, Wu Q, Chan HY, et al. (2006) Sall4 modulates embryonic stem cell pluripotency and early embryonic development by the transcriptional regulation of Pou5f1. Nature Cell Biology 8: 1114-23.

177. Yang J, Gao C, Chai L, Ma Y (2010) A novel SALL4/OCT4 transcriptional feedback network for pluripotency of embryonic stem cells. PLoS One 5: e10766.

178. Yuri S, Fujimura S, Nimura K, Takeda N, Toyooka Y, et al. (2009) Sall4 is essential for stabilization, but not for pluripotency, of embryonic stem cells by repressing aberrant trophectoderm gene expression. Stem Cells 27: 796-805.

179. Wu Q, Chen X, Zhang J, Loh YH, Low TY, et al. (2006) Sall4 interacts with Nanog and co-occupies Nanog genomic sites in embryonic stem cells. The Journal of biological chemistry 281: 24090-4.

180. Yuan P, Han J, Guo G, Orlov YL, Huss M, et al. (2009) Eset partners with Oct4 to restrict extraembryonic trophoblast lineage potential in embryonic stem cells. Genes & Development 23: 2507-20.

181. Bilodeau S, Kagey MH, Frampton GM, Rahl PB, Young RA (2009) SetDB1 contributes to repression of genes encoding developmental regulators and maintenance of ES cell state. Genes & Development 23: 2484-9.

182. Yeap LS, Hayashi K, Surani MA (2009) ERG-associated protein with SET domain (ESET)-Oct4 interaction regulates pluripotency and represses the trophectoderm lineage. Epigenetics Chromatin 2: 12.

183. Kele J, Andersson ER, Villaescusa JC, Cajanek L, Parish CL, et al. (2012) SFRP1 and SFRP2 dose-dependently regulate midbrain dopamine neuron development in vivo and in embryonic stem cells. Stem Cells 30: 865-75.

184. Dryden SC, Nahhas FA, Nowak JE, Goustin AS, Tainsky MA (2003) Role for human SIRT2 NAD-dependent deacetylase activity in control of mitotic exit in the cell cycle. Molecular and Cellular Biology 23: 3173-85.

185. Zhang F, Lundin M, Ristimaki A, Heikkila P, Lundin J, et al. (2003) Ski-related novel protein N (SnoN), a negative controller of transforming growth factor-beta signaling, is a prognostic marker in estrogen receptor-positive breast carcinomas. Cancer Res 63: 5005-10.

186. Ying QL, Nichols J, Chambers I, Smith A (2003) BMP induction of Id proteins suppresses differentiation and sustains embryonic stem cell self-renewal in collaboration with STAT3. Cell 115: 281-92.

187. Beattie GM, Lopez AD, Bucay N, Hinton A, Firpo MT, et al. (2005) Activin A maintains pluripotency of human embryonic stem cells in the absence of feeder layers. Stem Cells 23: 489-95.

188. Kidder BL, Palmer S, Knott JG (2009) SWI/SNF-Brg1 regulates self-renewal and occupies core pluripotency-related genes in embryonic stem cells. Stem Cells 27: 317-28.

189. Ho L, Jothi R, Ronan JL, Cui K, Zhao K, et al. (2009) An embryonic stem cell chromatin remodeling complex, esBAF, is an essential component of the core pluripotency transcriptional network. Proceedings of the National Academy of Sciences of the United States of America 106: 5187-91.

190. Klochendler-Yeivin A, Fiette L, Barra J, Muchardt C, Babinet C, et al. (2000) The murine SNF5/INI1 chromatin remodeling factor is essential for embryonic development and tumor suppression. EMBO Rep 1: 500-6.

191. Schaniel C, Ang YS, Ratnakumar K, Cormier C, James T, et al. (2009) Smarcc1/Baf155 couples self-renewal gene repression with changes in chromatin structure in mouse embryonic stem cells. Stem Cells 27: 2979-91.

192. Liu J, Feldman R, Zhang Z, Deardorff MA, Haverfield EV, et al. (2009) SMC1A expression and mechanism of pathogenicity in probands with X-Linked Cornelia de Lange syndrome. Hum Mutat 30: 1535-42.

193. Zhu G, Mehler MF, Zhao J, Yu Yung S, Kessler JA (1999) Sonic hedgehog and BMP2 exert opposing actions on proliferation and differentiation of embryonic neural progenitor cells. Developmental biology 215: 118-29.

194. Cai C, Thorne J, Grabel L (2008) Hedgehog serves as a mitogen and survival factor during embryonic stem cell neurogenesis. Stem Cells 26: 1097-108.

195. Palmqvist L, Glover CH, Hsu L, Lu M, Bossen B, et al. (2005) Correlation of murine embryonic stem cell gene expression profiles with functional measures of pluripotency. Stem Cells 23: 663-80.

196. Wang Z, Oron E, Nelson B, Razis S, Ivanova N (2012) Distinct lineage specification roles for NANOG, OCT4, and SOX2 in human embryonic stem cells. Cell Stem Cell 10: 440-54.

197. Yang HM, Do HJ, Oh JH, Kim JH, Choi SY, et al. (2005) Characterization of putative cis-regulatory elements that control the transcriptional activity of the human Oct4 promoter. J Cell Biochem 96: 821-30.

198. Wu DY, Yao Z (2006) Functional analysis of two Sp1/Sp3 binding sites in murine Nanog gene promoter. Cell research 16: 319-22.

199. Pacini S, Carnicelli V, Trombi L, Montali M, Fazzi R, et al. (2010) Constitutive expression of pluripotency-associated genes in mesodermal progenitor cells (MPCs). PLoS One 5: e9861.

200. Niwa H, Burdon T, Chambers I, Smith A (1998) Self-renewal of pluripotent embryonic stem cells is mediated via activation of STAT3. Genes & Development 12: 2048-60.

201. Luzzani C, Solari C, Losino N, Ariel W, Romorini L, et al. (2011) Modulation of chromatin modifying factors' gene expression in embryonic and induced pluripotent stem cells. Biochemical and biophysical research communications 410: 816-22.

202. Lee TI, Jenner RG, Boyer LA, Guenther MG, Levine SS, et al. (2006) Control of developmental regulators by Polycomb in human embryonic stem cells. Cell 125: 301-13.

203. Cole MF, Johnstone SE, Newman JJ, Kagey MH, Young RA (2008) Tcf3 is an integral component of the core regulatory circuitry of embryonic stem cells. Genes & Development 22: 746-55.

204. Marson A, Foreman R, Chevalier B, Bilodeau S, Kahn M, et al. (2008) Wnt signaling promotes reprogramming of somatic cells to pluripotency. Cell Stem Cell 3: 132-5.

205. Wray J, Kalkan T, Gomez-Lopez S, Eckardt D, Cook A, et al. (2012) Inhibition of glycogen synthase kinase-3 alleviates Tcf3 repression of the pluripotency network and increases embryonic stem cell resistance to differentiation. Nature Cell Biology 14: 555.

206. Loh Y-H, Wu Q, Chew J-L, Vega VB, Zhang W, et al. (2006) The Oct4 and Nanog transcription network regulates pluripotency in mouse embryonic stem cells. Nature Genetics 38: 431-40.

207. Fu TB, Virgilio L, Narducci MG, Facchiano A, Russo G, et al. (1994) Characterization and localization of the TCL-1 oncogene product. Cancer Res 54: 6297-301.

208. Shen MM, Wang H, Leder P (1997) A differential display strategy identifies Cryptic, a novel EGF-related gene expressed in the axial and lateral mesoderm during mouse gastrulation. Development 124: 429-42.

209. Parisi S, D'Andrea D, Lago CT, Adamson ED, Persico MG, et al. (2003) Nodal-dependent Cripto signaling promotes cardiomyogenesis and redirects the neural fate of embryonic stem cells. The Journal of cell biology 163: 303-14.

210. Pericuesta E, Ramirez MA, Villa-Diaz A, Relano-Gines A, Torres JM, et al. (2006) The proximal promoter region of mTert is sufficient to regulate telomerase activity in ES cells and transgenic animals. Reprod Biol Endocrinol 4: 5.

211. Hamid R, Brandt SJ (2009) Transforming growth-interacting factor (TGIF) regulates proliferation and differentiation of human myeloid leukemia cells. Mol Oncol 3: 451-63.

212. Dejosez M, Krumenacker JS, Zitur LJ, Passeri M, Chu LF, et al. (2008) Ronin is essential for embryogenesis and the pluripotency of mouse embryonic stem cells. Cell 133: 1162-74.

213. Babaie Y, Herwig R, Greber B, Brink TC, Wruck W, et al. (2007) Analysis of Oct4-dependent transcriptional networks regulating self-renewal and pluripotency in human embryonic stem cells. Stem Cells 25: 500-10.

214. Western P, Maldonado-Saldivia J, van den Bergen J, Hajkova P, Saitou M, et al. (2005) Analysis of Esg1 expression in pluripotent cells and the germline reveals similarities with Oct4 and Sox2 and differences between human pluripotent cell lines. Stem Cells 23: 1436-42.

215. Gattei V, Degan M, Gloghini A, De Iuliis A, Improta S, et al. (1997) CD30 ligand is frequently expressed in human hematopoietic malignancies of myeloid and lymphoid origin. Blood 89: 2048-59.

216. Marshall GM, Bell JL, Koach J, Tan O, Kim P, et al. (2010) TRIM16 acts as a tumour suppressor by inhibitory effects on cytoplasmic vimentin and nuclear E2F1 in neuroblastoma cells. Oncogene 29: 6172-83.

217. Jain AK, Allton K, Iacovino M, Mahen E, Milczarek RJ, et al. (2012) p53 regulates cell cycle and microRNAs to promote differentiation of human embryonic stem cells. PLoS Biol 10: e1001268.

218. van den Boom V, Kooistra SM, Boesjes M, Geverts B, Houtsmuller AB, et al. (2007) UTF1 is a chromatin-associated protein involved in ES cell differentiation. The Journal of cell biology 178: 913-24.

219. Kooistra SM, Thummer RP, Eggen BJ (2009) Characterization of human UTF1, a chromatin-associated protein with repressor activity expressed in pluripotent cells. Stem Cell Res 2: 211-8.

220. Wang J, Rao S, Chu J, Shen X, Levasseur DN, et al. (2006) A protein interaction network for pluripotency of embryonic stem cells. Nature 444: 364-8.

221. Ang YS, Tsai SY, Lee DF, Monk J, Su J, et al. (2011) Wdr5 mediates self-renewal and reprogramming via the embryonic stem cell core transcriptional network. Cell 145: 183-97.

222. Snow GE, Kasper AC, Busch AM, Schwarz E, Ewings KE, et al. (2009) Wnt pathway reprogramming during human embryonal carcinoma differentiation and potential for therapeutic targeting. BMC Cancer 9: 383.

223. Bouillet P, Oulad-Abdelghani M, Ward SJ, Bronner S, Chambon P, et al. (1996) A new mouse member of the Wnt gene family, mWnt-8, is expressed during early embryogenesis and is ectopically induced by retinoic acid. Mechanisms of Development 58: 141-52.

224. Morinaga T, Yasuda H, Hashimoto T, Higashio K, Tamaoki T (1991) A human alpha-fetoprotein enhancer-binding protein, ATBF1, contains four homeodomains and seventeen zinc fingers. Molecular and Cellular Biology 11: 6041-9.

225. Kaspar P, Dvorakova M, Kralova J, Pajer P, Kozmik Z, et al. (1999) Myb-interacting protein, ATBF1, represses transcriptional activity of Myb oncoprotein. The Journal of biological chemistry 274: 14422-8.

226. Shi W, Wang H, Pan G, Geng Y, Guo Y, et al. (2006) Regulation of the pluripotency marker Rex-1 by Nanog and Sox2. The Journal of biological chemistry 281: 23319-25.

227. Brown L, Brown S (2009) Zic2 is expressed in pluripotent cells in the blastocyst and adult brain expression overlaps with makers of neurogenesis. Gene Expr Patterns 9: 43-9.

228. Lim LS, Hong FH, Kunarso G, Stanton LW (2010) The pluripotency regulator Zic3 is a direct activator of the Nanog promoter in ESCs. Stem Cells 28: 1961-9.

229. Nyholm MK, Wu SF, Dorsky RI, Grinblat Y (2007) The zebrafish zic2a-zic5 gene pair acts downstream of canonical Wnt signaling to control cell proliferation in the developing tectum. Development 134: 735-46.

230. Wang ZX, Teh CH, Chan CM, Chu C, Rossbach M, et al. (2008) The transcription factor Zfp281 controls embryonic stem cell pluripotency by direct activation and repression of target genes. Stem Cells 26: 2791-9.
