# Supplementary material for: Genome-Wide Analysis Reveals the Unique Stem Cell Identity of Human Amniocytes
Source: PLoS One. 2013 Jan 10;8(1):e53372. doi: 10.1371/journal.pone.0053372 (PMC3542377; doi:10.1371/journal.pone.0053372)
Supplement: Table S3 — RNA-seq differential expression analysis. Table S3A shows transcripts identified by RNA-seq analysis (1) that were (A) consistently higher in ESC or (B) consistently higher in iPSC compared to amniocytes. Table S3B shows differential expression results that were sorted by the smallest to largest adjusted p-value. The top ten most significant genes for each comparison are listed. (PDF) [file pone.0053372.s004.pdf]

Table S3. RNA-seq differential expression analysis. Table S3A shows transcripts identified by RNA-seq analysis [1] that were (A) consistently higher in ESC or (B) consistently higher in iPSC compared to amniocytes. Table S3B shows differential expression results that were sorted by the smallest to largest adjusted p-value. The top ten most significant genes for each comparison are listed.

Table S3A. Selected 15 genes consistently higher in ESC and iPSC compared to amniocytes.

Gene	Function	ESC	iPSC	Amniocytes
**(A) Higher in ESC**				
Tcerg1l	Transcription elongation regulator 1	100.9	1.2	1.1
Fam19a5	Small secreted protein acting as regulator of immune and nervous cells.	350.6	1.8	11.9
Dpp6	Membrane serine protease, may play a role in brain development.	140.1	23.0	11.0
Irx2	Multiple roles during pattern formation	2500.1	603.3	38.2
Nnat	Neural differentiation	907.2	4.4	165.9
Sox11	Neural differentiation	18311.0	13583.3	924.3
**(B) Higher in iPSC**				
Tbx15	Transcriptional regulator of the skeleton, limb, vertebral column, and head	8.8	304.9	38.1
Pitx2	Eye, tooth, abdominal organs, left-right asymmetry.	22.1	107.5	104.6
Cr1l	Hematopoietic, lymphoid, Liver, spleen, thymus	3.6	76.7	0.4
Ctsf	Cysteine proteinases in the lysosomal proteolytic system	17.6	94.5	281.5
Grem1	BMP antagonist regulates organogenesis, body patterning, and differentiation.	210.8	238.7	11889.3
Ly6k	Carcinoembryonic antigen	1.1	2.1	23.8
Znf208	Transcriptional repression	17.4	327.0	9.0
Znf560	Transcriptional regulation	4.1	64.1	7.3
Znf662	Transcriptional regulation	3.0	5.2	44.4

Table S3B. The top ten most significant genes for each comparison.

**Amniocytes vs ESC (high in ESC)**								
Gene symbol	Ensgene id	Base Mean	Amniocyt mean	hESC mean	Fold Change	log2Fold Change	pval	padj
TDGF1	ENSG00000241186	3784.3	0.2	11153.4	0.0	-16.1	5.4E-240	1.6E-235

DPPA4	ENSG00000121570	8397.8	2.0	24747.6	0.0	-13.6	6.9E-229	6.8E-225
ZIC3	ENSG00000156925	1282.7	0.1	3780.5	0.0	-15.2	2.7E-209	2.0E-205
SOX2	ENSG00000181449	2431.0	0.8	7163.5	0.0	-13.1	1.4E-208	8.2E-205
LIN28B	ENSG00000187772	1301.6	0.3	3835.6	0.0	-13.6	1.3E-201	6.4E-198
L1TD1	ENSG00000240563	3806.7	3.2	11213.8	0.0	-11.8	1.0E-192	4.3E-189
LIN28A	ENSG00000131914	11676.9	15.9	34385.1	0.0	-11.1	8.2E-186	2.7E-182
PTPRZ1	ENSG00000106278	1559.7	2.0	4593.2	0.0	-11.2	3.9E-181	1.2E-177
SLC7A3	ENSG00000165349	1945.2	2.8	5727.6	0.0	-11.0	7.2E-177	1.9E-173
LECT1	ENSG00000136110	550.8	0.6	1622.2	0.0	-11.3	1.2E-166	2.5E-163
**Amniocytes vs ESC_(high in Amniocytes)**								
Gene symbol	Ensgene id	baseMean	Amniocyt mean	hESC mean	Fold Change	log2Fold Change	pval	padj
NNMT	ENSG00000166741	7082.3	10718.5	1.3	7990.2	13.0	1.09E-106	4.01E-104
IGFBP7	ENSG00000163453	5600.8	8476.4	1.0	8100.9	13.0	6.08E-94	1.42E-91
PAX8	ENSG00000125618	2585.9	3913.4	0.8	4746.4	12.2	6.62E-94	1.54E-91
HOXA3	ENSG00000105997	580.9	879.2	0.0	1000000.0	19.9	1.35E-86	2.72E-84
HOXA9	ENSG00000078399	457.3	692.2	0.0	1000000.0	19.9	8.06E-83	1.40E-80
HOXB9	ENSG00000170689	757.9	1146.7	0.6	1993.2	11.0	2.87E-80	4.58E-78
HOXB6	ENSG00000108511	372.3	563.5	0.0	26420.0	14.7	1.63E-78	2.47E-76
ANKRD1	ENSG00000148677	11249.0	17016.4	17.9	952.7	9.9	5.93E-78	8.70E-76
HOXA7	ENSG00000122592	400.3	605.7	0.2	2599.6	11.3	4.69E-76	6.41E-74
LRRN4	ENSG00000125872	1490.0	2253.7	2.7	821.1	9.7	5.54E-76	7.49E-74
**Amniocytes vs iPSC_(high in iPSC)**								
Gene symbol	Ensgene id	baseMean	Amniocyt mean	iPSC mean	Fold Change	log2Fold Change	pval	padj
LIN28A	ENSG00000131914	7848.4	16.0	31998.4	0.0	-11.0	>E-306	>E-306
PTPRZ1	ENSG00000106278	1053.0	2.0	4293.5	0.0	-11.1	>E-306	>E-306
ADCY2	ENSG00000078295	965.4	1.5	3937.2	0.0	-11.3	>E-306	>E-306
L1TD1	ENSG00000240563	2717.9	3.2	11088.1	0.0	-11.8	>E-306	>E-306
DPPA4	ENSG00000121570	7298.4	2.0	29795.7	0.0	-13.9	>E-306	>E-306
LIN28B	ENSG00000187772	1165.3	0.3	4757.2	0.0	-13.9	>E-306	>E-306
VRTN	ENSG00000133980	543.8	0.1	2220.1	0.0	-14.2	>E-306	>E-306
ZIC3	ENSG00000156925	917.5	0.1	3746.3	0.0	-15.1	>E-306	>E-306
TDGF1	ENSG00000241186	3719.8	0.2	15188.7	0.0	-16.5	>E-306	>E-306
ITM2A	ENSG00000078596	1254.4	4.0	5109.8	0.0	-10.3	1.8E-306	4.4E-303
**Amniocytes vs iPSC_(high in Amniocytes)**								
Gene symbol	Ensgene id	baseMean	Amniocyt mean	iPSC mean	Fold Change	log2Fold Change	pval	padj
NNMT	ENSG00000166741	8096.1	10721.7	0.3	38988.1	15.3	4.7E-152	9.5E-150
PAX8	ENSG00000125618	2956.8	3915.6	0.4	9721.0	13.2	2.1E-130	3.1E-128
MVP	ENSG00000013364	4493.7	5950.5	1.8	3336.2	11.7	1.3E-128	1.8E-126
HOXB9	ENSG00000170689	866.0	1146.8	0.1	13380.1	13.7	6.3E-109	5.8E-107

KCNS3	ENSG00000170745	343.9	36.2	1292.8	0.0	-5.2	1.4E-108	1.3E-106
HOXA7	ENSG00000122592	457.8	606.3	0.0	1000000.0	19.9	2.3E-97	1.7E-95
HOXA9	ENSG00000078399	523.0	692.6	0.2	4160.7	12.0	4.2E-97	3.2E-95
HOXA3	ENSG00000105997	664.3	879.7	0.2	3557.3	11.8	1.7E-96	1.3E-94
BMP1	ENSG00000168487	3506.7	4639.3	14.6	318.1	8.3	6.0E-93	4.1E-91
HOXC10	ENSG00000180818	358.9	475.3	0.0	1000000.0	19.9	2.4E-92	1.6E-90
**Gestational age (High in Early GA)**								
Gene symbol	Ensgene id	baseMean	Mean early	Mean late	Fold Change	log2Fold Change	pval	padj
IGF2BP1	ENSG00000159217	1190.8	1832.1	514.0	3.6	1.8	8.6E-14	1.2E-10
Tbx18	ENSG00000112837	55.8	87.2	22.6	3.9	2.0	5.6E-11	3.9E-08
COL25A1	ENSG00000188517	362.9	584.2	129.3	4.5	2.2	2.6E-10	1.5E-07
DSC3	ENSG00000134762	501.7	868.3	114.8	7.6	2.9	2.0E-08	5.4E-06
HIF1A	ENSG00000100644	8355.9	12065.4	4440.3	2.7	1.4	5.3E-08	1.2E-05
FOXP2	ENSG00000128573	39.6	61.1	16.9	3.6	1.9	7.3E-08	1.6E-05
WDR38	ENSG00000136918	5.2	9.2	0.9	10.7	3.4	9.2E-08	1.9E-05
Gata6	ENSG00000141448	567.2	820.8	299.5	2.7	1.5	1.2E-07	2.3E-05
LTBP1	ENSG00000049323	1857.6	2812.5	849.8	3.3	1.7	1.3E-07	2.6E-05
BACH1	ENSG00000156273	1103.3	1567.8	612.9	2.6	1.4	1.8E-07	3.2E-05
**Gestational age (High in Late GA)**								
Gene symbol	Ensgene id	baseMean	Mean early	Mean late	Fold Change	log2Fold Change	pval	padj
MRPL33	ENSG00000243147	2736.3	1208.1	4349.4	0.3	-1.8	3.5E-12	3.9E-09
RPS21	ENSG00000171858	8517.5	3807.0	13489.7	0.3	-1.8	3.9E-12	4.1E-09
RPS29	ENSG00000213741	16486.1	7562.2	25905.7	0.3	-1.8	1.1E-11	1.1E-08
C3orf78	ENSG00000168273	705.7	327.2	1105.3	0.3	-1.8	2.4E-11	2.1E-08
C22orf26	ENSG00000182257	45.5	19.5	73.0	0.3	-1.9	3.4E-11	2.6E-08
NDUFB1	ENSG00000183648	2039.1	1005.6	3129.9	0.3	-1.6	2.9E-10	1.6E-07
SNRPG	ENSG00000143977	2762.2	1392.3	4208.2	0.3	-1.6	3.4E-10	1.9E-07
FAM36A	ENSG00000203667	532.8	273.3	806.7	0.3	-1.6	4.1E-10	2.2E-07
HoxD4	ENSG00000170166	107.0	52.9	164.1	0.3	-1.6	4.1E-10	2.2E-07
FOLR1	ENSG00000110195	39.4	7.0	73.6	0.1	-3.4	1.4E-09	6.4E-07
**Culture time (High in Time 1)**								
Gene symbol	Ensgene id	baseMean	Mean Time_1	Mean Time_3	Fold Change	log2Fold Change	pval	padj
CA3	ENSG00000164879	30.0	57.2	2.8	20.7	4.4	2.8E-13	8.2E-09
PLBD1	ENSG00000121316	60.2	106.4	14.1	7.5	2.9	1.2E-12	1.2E-08
CPXM1	ENSG00000088882	114.0	209.1	18.8	11.1	3.5	1.8E-12	1.3E-08
E2F2	ENSG00000007968	40.2	76.1	4.2	18.2	4.2	3.9E-12	2.3E-08
KCNE3	ENSG00000175538	13.7	24.3	3.1	7.9	3.0	1.4E-11	4.9E-08
SFXN2	ENSG00000156398	134.7	220.1	49.3	4.5	2.2	1.6E-11	4.9E-08
HAAO	ENSG00000162882	9.5	18.1	0.8	21.9	4.5	7.0E-11	1.6E-07

FAM111B	ENSG00000189057	78.9	150.7	7.0	21.4	4.4	1.4E-10	2.9E-07
BRCA2	ENSG00000139618	59.8	108.3	11.2	9.7	3.3	2.2E-10	4.3E-07
ORC1	ENSG00000085840	98.3	180.5	16.1	11.2	3.5	9.9E-10	1.8E-06
**Culture Time (High in Time3)**								
Gene symbol	Ensgene id	baseMean	Mean Time_1	Mean Time_3	Fold Change	log2Fold Change	pval	padj
CDKN2A	ENSG00000147889	2214.8	775.3	3654.3	0.2	-2.2	6.3E-13	9.3E-09
FBXL16	ENSG00000127585	52.1	17.2	86.9	0.2	-2.3	4.9E-12	2.4E-08
C21orf7	ENSG00000156265	1169.0	362.3	1975.8	0.2	-2.4	9.9E-12	4.2E-08
EEF1A2	ENSG00000101210	2254.2	581.5	3927.0	0.1	-2.8	1.7E-11	4.9E-08
SPRN	ENSG00000203772	153.6	56.7	250.5	0.2	-2.1	5.1E-11	1.3E-07
OXTR	ENSG00000180914	1614.3	574.8	2653.8	0.2	-2.2	3.3E-09	4.3E-06
CALY	ENSG00000130643	28.2	9.3	47.1	0.2	-2.3	5.9E-09	6.7E-06
CCND2	ENSG00000118971	2812.5	1245.0	4380.1	0.3	-1.8	6.2E-09	6.8E-06
PDE1C	ENSG00000154678	876.9	343.7	1410.1	0.2	-2.0	6.9E-09	7.2E-06
SULT4A1	ENSG00000130540	38.1	15.7	60.6	0.3	-1.9	2.9E-08	1.9E-05
**Gender (High in Male)**								
Gene symbol	Ensgene id	baseMean	mean_M	Mean F	Fold Change	log2Fold Change	pval	padj
RPS4Y1	ENSG00000129824	1194.0	4015.0	0.5	8092.3	13.0	5.0E-239	1.5E-234
KDM5D	ENSG00000012817	137.5	462.4	0.0	1048576.0	20.0	1.3E-166	2.0E-162
TTTY15	ENSG00000233864	139.8	470.3	0.0	14841.2	13.9	1.4E-157	1.3E-153
CYorf15A.1	ENSG00000099749	61.1	205.2	0.1	1662.2	10.7	1.6E-126	1.2E-122
UTY	ENSG00000183878	66.6	218.8	2.3	96.7	6.6	7.5E-92	3.7E-88
NLGN4Y	ENSG00000165246	122.5	407.5	1.9	216.5	7.8	8.6E-87	3.6E-83
DDX3Y	ENSG00000067048	282.7	943.7	3.0	312.4	8.3	2.9E-79	9.7E-76
USP9Y	ENSG00000114374	144.9	482.1	2.2	217.6	7.8	2.8E-79	9.7E-76
EIF1AY	ENSG00000198692	388.2	1297.9	3.4	386.9	8.6	3.5E-69	1.0E-65
TTTY14	ENSG00000176728	11.3	37.9	0.0	1048576.0	20.0	3.2E-57	8.5E-54
CYorf15B.1	ENSG00000131002	38.0	127.6	0.2	813.4	9.7	3.5E-57	8.5E-54
**Gender (High in Female)**								
Gene symbol	Ensgene id	baseMean	Mean M	Mean F	Fold Change	log2Fold Change	pval	padj
XIST	ENSG00000229807	2903.1	0.2	4131.3	0.0	-14.3	2.9E-117	1.7E-113
TFAP2B	ENSG00000008196	50.4	0.5	71.5	0.0	-7.1	5.9E-07	6.4E-04
CYBA	ENSG00000051523	8507.9	4038.7	10398.8	0.4	-1.4	5.7E-06	4.4E-03
GPC4	ENSG00000076716	664.5	330.2	805.9	0.4	-1.3	6.9E-06	5.3E-03
PCDHA4	ENSG00000204967	5.7	1.1	7.6	0.1	-2.7	9.9E-06	6.3E-03
TMEM139	ENSG00000178826	270.5	117.3	335.3	0.3	-1.5	1.1E-05	6.7E-03
FAM36A	ENSG00000203667	532.8	231.6	660.2	0.4	-1.5	1.4E-05	7.9E-03
MPZL3	ENSG00000160588	226.1	114.0	273.5	0.4	-1.3	1.5E-05	8.0E-03
NCF2	ENSG00000116701	935.0	415.8	1154.7	0.4	-1.5	1.6E-05	8.1E-03

HBQ1	ENSG00000086506	22.5	8.8	28.3	0.3	-1.7	1.8E-05	8.9E-03

REFERENCES

1. Phanstiel, D.H., Brumbaugh, J., Wenger, C.D., Tian, S., Probasco, M.D., Bailey, D.J., Swaney, D.L., Tervo, M.A., Bolin, J.M., Ruotti, V., et al. 2011. Proteomic and phosphoproteomic comparison of human ES and iPS cells. *Nat Meth* 8:821-827.
