# Supplementary material for: Genome-Wide Analysis Reveals the Unique Stem Cell Identity of Human Amniocytes
Source: PLoS One. 2013 Jan 10;8(1):e53372. doi: 10.1371/journal.pone.0053372 (PMC3542377; doi:10.1371/journal.pone.0053372)
Supplement: Table S4 — Reference list for 89 embryonic stem cell repressors. The 89 putative repressors were included in our list based on the following two criteria; 1) each gene has a reported repressive function during development and 2) each gene was reliably detected at significant levels in our RNA-seq dataset. (PDF) [file pone.0053372.s005.pdf]

Table S4. Reference list for 89 embryonic stem cell repressors. The 89 putative repressors were included in our list based on the following two criteria; 1) each gene has a reported repressive function during development and 2) each gene was reliably detected at significant levels in our RNA-seq dataset.

Gene_ Symbol	Ensgene_id	Refseq_id	Function	References
Bcl6	ENSG00000113916	NM_138931	Stem Cell Repressor	[1]
Cbx2	ENSG00000173894	NM_032647	Stem Cell Repressor	[2,3]
Cbx3	ENSG00000122565	NM_007276	Stem Cell Repressor	[4]
Cbx4	ENSG00000141582	NM_003655	Stem Cell Repressor	[5,6]
Cbx6	ENSG00000183741	NM_014292	Stem Cell Repressor	[7]
Cbx7	ENSG00000100307	NM_175709	Stem Cell Repressor	[2,8]
Cbx8	ENSG00000141570	NM_020649	Stem Cell Repressor	[7]
Ctbp1	ENSG00000159692	NM_001328	Stem Cell Repressor	[9,10]
Dek	ENSG00000124795	NM_001134709	Stem Cell Repressor	[11,12]
Dmap1	ENSG00000178028	NM_019100	Stem Cell Repressor	[13,14]
Dnmt3a	ENSG00000119772	NM_022552	Stem Cell Repressor	[15,16]
Dnmt3b	ENSG00000088305	NM_006892	Stem Cell Repressor	[17]
E2f4	ENSG00000205250	NM_001950	Stem Cell Repressor	[18-20]
E2f6	ENSG00000169016	NM_001952	Stem Cell Repressor	[21,22]
Erf	ENSG00000105722	NM_006494	Stem Cell Repressor	[23]
Etv6	ENSG00000139083	NM_001987	Stem Cell Repressor	[24,25]
Foxd3	ENSG00000187140	NM_030625	Stem Cell Repressor	[26,27]
Foxm1	ENSG00000111206	NM_021953	Stem Cell Repressor	[28,29]
Foxp1	ENSG00000114861	NM_032682	Stem Cell Repressor	[30,31]
Gata2	ENSG00000179348	NM_032638	Stem Cell Repressor	[32,33]
Gatad2a	ENSG00000167491	NM_017660	Stem Cell Repressor	[34]

Gatad2b	ENSG00000143614	NM_020699	Stem Cell Repressor	[34]
Glis1	ENSG00000174332	NM_147193	Stem Cell Repressor	[35]
Glis2	ENSG00000126603	NM_032575	Stem Cell Repressor	[36,37]
Glis3	ENSG00000107249	NM_152629	Stem Cell Repressor	[38]
Hdac1	ENSG00000116478	NM_004964	Stem Cell Repressor	[34,39,40]
Hdac10	ENSG00000100429	NM_032019	Stem Cell Repressor	[41]
Hdac11	ENSG00000163517	NM_024827	Stem Cell Repressor	[42,43]
Hdac2	ENSG00000196591	NM_001527	Stem Cell Repressor	[34,44]
Hdac3	ENSG00000171720	NM_003883	Stem Cell Repressor	[45-47]
Hdac4	ENSG00000068024	NM_006037	Stem Cell Repressor	[48,49]
Hdac5	ENSG00000108840	NM_001015053	Stem Cell Repressor	[50,51]
Hdac6	ENSG00000094631	NM_006044	Stem Cell Repressor	[52,53]
Hdac7	ENSG00000061273	NM_001098416	Stem Cell Repressor	[51,54]
Hdac8	ENSG00000147099	NM_018486	Stem Cell Repressor	[55]
Hdac9	ENSG00000048052	NM_001204144	Stem Cell Repressor	[56]
Hes1	ENSG00000114315	NM_005524	Stem Cell Repressor	[57-59]
Hes6	ENSG00000144485	NM_018645	Stem Cell Repressor	[60,61]
Hipk2	ENSG00000064393	NM_022740	Stem Cell Repressor	[62,63]
Hira	ENSG00000100084	NM_003325	Stem Cell Repressor	[64]
Hsf1	ENSG00000185122	NM_005526	Stem Cell Repressor	[65,66]
Kdm2b	ENSG00000089094	NM_032590	Stem Cell Repressor	[1]
Klf10	ENSG00000155090	NM_001032282	Stem Cell Repressor	[67]
Klf11	ENSG00000172059	NM_003597	Stem Cell Repressor	[68,69]
Klf13	ENSG00000169926	NM_015995	Stem Cell Repressor	[70]
Klf15	ENSG00000163884	NM_014079	Stem Cell Repressor	[71]

Klf16	ENSG00000129911	NM_031918	Stem Cell Repressor	[71]
Klf3	ENSG00000109787	NM_016531	Stem Cell Repressor	[72]
Klf6	ENSG00000067082	NM_001160124	Stem Cell Repressor	[73,74]
Klf7	ENSG00000118263	NM_003709	Stem Cell Repressor	[75]
Klf8	ENSG00000102349	NM_007250	Stem Cell Repressor	[76]
Klf9	ENSG00000119138	NM_001206	Stem Cell Repressor	[77]
L3mbtl1	ENSG00000185513	NM_032107	Stem Cell Repressor	[25,78,79]
Mbd2	ENSG00000134046	NM_003927	Stem Cell Repressor	[16,80]
Mbd3	ENSG00000071655	NM_003926	Stem Cell Repressor	[80]
Mecom	ENSG00000085276	NM_005241	Stem Cell Repressor	[81-83]
Mnt	ENSG00000070444	NM_020310	Stem Cell Repressor	[84,85]
Mta1	ENSG00000182979	NM_001203258	Stem Cell Repressor	[34,86,87]
Mta2	ENSG00000149480	NM_004739	Stem Cell Repressor	[34,87]
Mta3	ENSG00000057935	NM_020744	Stem Cell Repressor	[87,88]
Ncor1	ENSG00000141027	NM_006311	Stem Cell Repressor	[89]
Ncor2	ENSG00000196498	NM_006312	Stem Cell Repressor	[90]
Nfic	ENSG00000141905	NM_005597	Stem Cell Repressor	[91-93]
Nfix	ENSG00000008441	NM_002501	Stem Cell Repressor	[93-95]
Nr0b1	ENSG00000169297	NM_000475	Stem Cell Repressor	[96,97]
Pias3	ENSG00000131788	NM_006099	Stem Cell Repressor	[98,99]
Pias4	ENSG00000105229	NM_015897	Stem Cell Repressor	[100-102]
Pml	ENSG00000140464	NM_002675	Stem Cell Repressor	[34,103]
Rbl1	ENSG00000080839	NM_002895	Stem Cell Repressor	[104,105]
Rcor1	ENSG00000089902	NM_015156	Stem Cell Repressor	[106-108]
Runx1t1	ENSG00000079102	NM_004349	Stem Cell Repressor	[109-111]

Rybp	ENSG00000163602	NM_012234	Stem Cell Repressor	[21,112,113]
Sbno2	ENSG00000064932	NM_014963	Stem Cell Repressor	[114]
Sin3a	ENSG00000169375	NM_015477	Stem Cell Repressor	[34,115]
Sin3b	ENSG00000127511	NM_015260	Stem Cell Repressor	[115-117]
Sirt1	ENSG00000096717	NM_001142498	Stem Cell Repressor	[118-121]
Ski	ENSG00000157933	NM_003036	Stem Cell Repressor	[122-125]
Tet1	ENSG00000138336	NM_030625	Stem Cell Repressor	[126,127]
Thap7	ENSG00000184436	NM_030573	Stem Cell Repressor	[128,129]
Tle1	ENSG00000196781	NM_005077	Stem Cell Repressor	[130-133]
Tle3	ENSG00000140332	NM_005078	Stem Cell Repressor	[134]
Tle4	ENSG00000106829	XM_212237	Stem Cell Repressor	[135-137]
Tle6	ENSG00000104953	NM_024760	Stem Cell Repressor	[138]
Uty	ENSG00000183878	NM_182660	Stem Cell Repressor	[139]
Yaf2	ENSG00000015153	NM_001190977	Stem Cell Repressor	[140]
YY1	ENSG00000100811	NM_003403	Stem Cell Repressor	[140,141]
Zhx1	ENSG00000165156	NM_001017926	Stem Cell Repressor	[142]
Zmynd15	ENSG00000141497	NM_032265	Stem Cell Repressor	[143]

REFERENCES

1. Sanchez C, Sanchez I, Demmers JA, Rodriguez P, Strouboulis J, et al. (2007) Proteomics analysis of Ring1B/Rnf2 interactors identifies a novel complex with the Fbxl10/Jhdm1B histone demethylase and the Bcl6 interacting corepressor. Mol Cell Proteomics 6: 820-34.

2. Maksakova IA, Goyal P, Bullwinkel J, Brown JP, Bilenky M, et al. (2011) H3K9me3-binding proteins are dispensable for SETDB1/H3K9me3-dependent retroviral silencing. Epigenetics Chromatin 4(1):12.

3. Volkel P, Le Faou P, Vandamme J, Pira D, Angrand PO (2012) A human Polycomb isoform lacking the Pc box does not participate to PRC1 complexes but forms protein assemblies and represses transcription. Epigenetics 7(5):482-91..

4. Trojer P, Cao AR, Gao Z, Li Y, Zhang J, et al. (2011) L3MBTL2 protein acts in concert with PcG protein-mediated monoubiquitination of H2A to establish a repressive chromatin structure. Mol Cell 42: 438-50.

5. Li B, Zhou J, Liu P, Hu J, Jin H, et al. (2007) Polycomb protein Cbx4 promotes SUMO modification of de novo DNA methyltransferase Dnmt3a. Biochem J 405: 369-78.

6. Kang X, Qi Y, Zuo Y, Wang Q, Zou Y, et al. (2010) SUMO-specific protease 2 is essential for suppression of polycomb group protein-mediated gene silencing during embryonic development. Mol Cell 38: 191-201.

7. Bernstein E, Duncan EM, Masui O, Gil J, Heard E, et al. (2006) Mouse polycomb proteins bind differentially to methylated histone H3 and RNA and are enriched in facultative heterochromatin. Molecular and Cellular Biology 26: 2560-9.

8. O'Loghlen A, Munoz-Cabello AM, Gaspar-Maia A, Wu HA, Banito A, et al. (2012) MicroRNA regulation of Cbx7 mediates a switch of Polycomb orthologs during ESC differentiation. Cell Stem Cell 10: 33-46.

9. Furusawa T, Moribe H, Kondoh H, Higashi Y (1999) Identification of CtBP1 and CtBP2 as corepressors of zinc finger-homeodomain factor deltaEF1. Molecular and Cellular Biology 19: 8581-90.

10. Kim JH, Cho EJ, Kim ST, Youn HD (2005) CtBP represses p300-mediated transcriptional activation by direct association with its bromodomain. Nat Struct Mol Biol 12: 423-8.

11. Hollenbach AD, McPherson CJ, Mientjes EJ, Iyengar R, Grosveld G (2002) Daxx and histone deacetylase II associate with chromatin through an interaction with core histones and the chromatin-associated protein Dek. J Cell Sci 115: 3319-30.

12. Kim DW, Kim JY, Choi S, Rhee S, Hahn Y, et al. (2010) Transcriptional regulation of 1-cys peroxiredoxin by the proto-oncogene protein DEK. Mol Med Report 3: 877-81.

13. Rountree MR, Bachman KE, Baylin SB (2000) DNMT1 binds HDAC2 and a new co-repressor, DMAP1, to form a complex at replication foci. Nature Genetics 25: 269-77.

14. Liu Z, Fisher RA (2004) RGS6 interacts with DMAP1 and DNMT1 and inhibits DMAP1 transcriptional repressor activity. The Journal of biological chemistry 279: 14120-8.

15. Challen GA, Sun D, Jeong M, Luo M, Jelinek J, et al. (2012) Dnmt3a is essential for hematopoietic stem cell differentiation. Nature Genetics 44: 23-31.

16. Gu P, Xu X, Le Menuet D, Chung AC, Cooney AJ (2011) Differential recruitment of methyl CpG-binding domain factors and DNA methyltransferases by the orphan receptor germ cell nuclear factor initiates the repression and silencing of Oct4. Stem Cells 29: 1041-51.

17. Rodic N, Oka M, Hamazaki T, Murawski MR, Jorgensen M, et al. (2005) DNA methylation is required for silencing of ant4, an adenine nucleotide translocase

selectively expressed in mouse embryonic stem cells and germ cells. Stem Cells 23: 1314-23.

18. Hateboer G, Kerkhoven RM, Shvarts A, Bernards R, Beijersbergen RL (1996) Degradation of E2F by the ubiquitin-proteasome pathway: regulation by retinoblastoma family proteins and adenovirus transforming proteins. Genes & Development 10: 2960-70.

19. Li JM, Hu PP, Shen X, Yu Y, Wang XF (1997) E2F4-RB and E2F4-p107 complexes suppress gene expression by transforming growth factor beta through E2F binding sites. Proceedings of the National Academy of Sciences of the United States of America 94: 4948-53.

20. Furukawa Y, Iwase S, Kikuchi J, Nakamura M, Yamada H, et al. (1999) Transcriptional repression of the E2F-1 gene by interferon-alpha is mediated through induction of E2F-4/pRB and E2F-4/p130 complexes. Oncogene 18: 2003-14.

21. Trimarchi JM, Fairchild B, Wen J, Lees JA (2001) The E2F6 transcription factor is a component of the mammalian Bmi1-containing polycomb complex. Proceedings of the National Academy of Sciences of the United States of America 98: 1519-24.

22. Attwooll C, Oddi S, Cartwright P, Prosperini E, Agger K, et al. (2005) A novel repressive E2F6 complex containing the polycomb group protein, EPC1, that interacts with EZH2 in a proliferation-specific manner. The Journal of biological chemistry 280: 1199-208.

23. Sgouras DN, Athanasiou MA, Beal GJ, Jr., Fisher RJ, Blair DG, et al. (1995) ERF: an ETS domain protein with strong transcriptional repressor activity, can suppress ets-associated tumorigenesis and is regulated by phosphorylation during cell cycle and mitogenic stimulation. Embo J 14: 4781-93.

24. Chakrabarti SR, Nucifora G (1999) The leukemia-associated gene TEL encodes a transcription repressor which associates with SMRT and mSin3A. Biochemical and biophysical research communications 264: 871-7.

25. Boccuni P, MacGrogan D, Scandura JM, Nimer SD (2003) The human L(3)MBT polycomb group protein is a transcriptional repressor and interacts physically and functionally with TEL (ETV6). The Journal of biological chemistry 278: 15412-20.

26. Yaklichkin S, Steiner AB, Lu Q, Kessler DS (2007) FoxD3 and Grg4 physically interact to repress transcription and induce mesoderm in Xenopus. The Journal of biological chemistry 282: 2548-57.

27. Thomas AJ, Erickson CA (2009) FOXD3 regulates the lineage switch between neural crest-derived glial cells and pigment cells by repressing MITF through a non-canonical mechanism. Development 136: 1849-58.

28. Xie Z, Tan G, Ding M, Dong D, Chen T, et al. (2010) Foxm1 transcription factor is required for maintenance of pluripotency of P19 embryonal carcinoma cells. Nucleic Acids Res 38: 8027-38.

29. Penzo M, Massa PE, Olivotto E, Bianchi F, Borzi RM, et al. (2009) Sustained NF-kappaB activation produces a short-term cell proliferation block in conjunction with repressing effectors of cell cycle progression controlled by E2F or FoxM1. Journal of cellular physiology 218: 215-27.

30. Shu W, Yang H, Zhang L, Lu MM, Morrisey EE (2001) Characterization of a new subfamily of winged-helix/forkhead (Fox) genes that are expressed in the lung and act as transcriptional repressors. The Journal of biological chemistry 276: 27488-97.

31. Wang B, Lin D, Li C, Tucker P (2003) Multiple domains define the expression and regulatory properties of Foxp1 forkhead transcriptional repressors. The Journal of biological chemistry 278: 24259-68.

32. Grass JA, Boyer ME, Pal S, Wu J, Weiss MJ, et al. (2003) GATA-1-dependent transcriptional repression of GATA-2 via disruption of positive autoregulation and domain-wide chromatin remodeling. Proceedings of the National Academy of Sciences of the United States of America 100: 8811-6.

33. Martowicz ML, Grass JA, Bresnick EH (2006) GATA-1-mediated transcriptional repression yields persistent transcription factor IIB-chromatin complexes. The Journal of biological chemistry 281: 37345-52.

34. Liang J, Wan M, Zhang Y, Gu P, Xin H, et al. (2008) Nanog and Oct4 associate with unique transcriptional repression complexes in embryonic stem cells. Nature Cell Biology 10: 731-9.

35. Kim YS, Lewandoski M, Perantoni AO, Kurebayashi S, Nakanishi G, et al. (2002) Identification of Glis1, a novel Gli-related, Kruppel-like zinc finger protein containing transactivation and repressor functions. The Journal of biological chemistry 277: 30901-13.

36. Zhang F, Nakanishi G, Kurebayashi S, Yoshino K, Perantoni A, et al. (2002) Characterization of Glis2, a novel gene encoding a Gli-related, Kruppel-like transcription factor with transactivation and repressor functions. Roles in kidney development and neurogenesis. The Journal of biological chemistry 277: 10139-49.

37. Kim SC, Kim YS, Jetten AM (2005) Kruppel-like zinc finger protein Gli-similar 2 (Glis2) represses transcription through interaction with C-terminal binding protein 1 (CtBP1). Nucleic Acids Res 33: 6805-15.

38. Kim YS, Nakanishi G, Lewandoski M, Jetten AM (2003) GLIS3, a novel member of the GLIS subfamily of Kruppel-like zinc finger proteins with repressor and activation functions. Nucleic Acids Res 31: 5513-25.

39. Lagger G, O'Carroll D, Rembold M, Khier H, Tischler J, et al. (2002) Essential function of histone deacetylase 1 in proliferation control and CDK inhibitor repression. Embo J 21: 2672-81.

40. Kang BG, Shin JH, Yi JK, Kang HC, Lee JJ, et al. (2007) Corepressor MMTR/DMAP1 is involved in both histone deacetylase 1- and TFIIH-mediated transcriptional repression. Molecular and Cellular Biology 27: 3578-88.

41. Kao HY, Lee CH, Komarov A, Han CC, Evans RM (2002) Isolation and characterization of mammalian HDAC10, a novel histone deacetylase. The Journal of biological chemistry 277: 187-93.

42. Feng W, Lu Z, Luo RZ, Zhang X, Seto E, et al. (2007) Multiple histone deacetylases repress tumor suppressor gene ARHI in breast cancer. Int J Cancer 120: 1664-8.

43. Gao L, Cueto MA, Asselbergs F, Atadja P (2002) Cloning and functional characterization of HDAC11, a novel member of the human histone deacetylase family. The Journal of biological chemistry 277: 25748-55.

44. LeBoeuf M, Terrell A, Trivedi S, Sinha S, Epstein JA, et al. (2010) Hdac1 and Hdac2 act redundantly to control p63 and p53 functions in epidermal progenitor cells. Developmental Cell 19: 807-18.

45. Guenther MG, Lane WS, Fischle W, Verdin E, Lazar MA, et al. (2000) A core SMRT corepressor complex containing HDAC3 and TBL1, a WD40-repeat protein linked to deafness. Genes & Development 14: 1048-57.

46. Wen YD, Perissi V, Staszewski LM, Yang WM, Krones A, et al. (2000) The histone deacetylase-3 complex contains nuclear receptor corepressors. Proceedings of the National Academy of Sciences of the United States of America 97: 7202-7.

47. Li J, Wang J, Nawaz Z, Liu JM, Qin J, et al. (2000) Both corepressor proteins SMRT and N-CoR exist in large protein complexes containing HDAC3. Embo J 19: 4342-50.

48. Miska EA, Karlsson C, Langley E, Nielsen SJ, Pines J, et al. (1999) HDAC4 deacetylase associates with and represses the MEF2 transcription factor. Embo J 18: 5099-107.

49. Wang AH, Bertos NR, Vezmar M, Pelletier N, Crosato M, et al. (1999) HDAC4, a human histone deacetylase related to yeast HDA1, is a transcriptional corepressor. Molecular and Cellular Biology 19: 7816-27.

50. Huang EY, Zhang J, Miska EA, Guenther MG, Kouzarides T, et al. (2000) Nuclear receptor corepressors partner with class II histone deacetylases in a Sin3-independent repression pathway. Genes & Development 14: 45-54.

51. Kao HY, Downes M, Ordentlich P, Evans RM (2000) Isolation of a novel histone deacetylase reveals that class I and class II deacetylases promote SMRT-mediated repression. Genes & Development 14: 55-66.

52. Westendorf JJ, Zaidi SK, Cascino JE, Kahler R, van Wijnen AJ, et al. (2002) Runx2 (Cbfa1, AML-3) interacts with histone deacetylase 6 and represses the p21(CIP1/WAF1) promoter. Molecular and Cellular Biology 22: 7982-92.

53. Ling L, Lobie PE (2004) RhoA/ROCK activation by growth hormone abrogates p300/histone deacetylase 6 repression of Stat5-mediated transcription. The Journal of biological chemistry 279: 32737-50.

54. Lemercier C, Brocard MP, Puvion-Dutilleul F, Kao HY, Albagli O, et al. (2002) Class II histone deacetylases are directly recruited by BCL6 transcriptional repressor. The Journal of biological chemistry 277: 22045-52.

55. Somoza JR, Skene RJ, Katz BA, Mol C, Ho JD, et al. (2004) Structural snapshots of human HDAC8 provide insights into the class I histone deacetylases. Structure 12: 1325-34.

56. Zhou X, Richon VM, Rifkind RA, Marks PA (2000) Identification of a transcriptional repressor related to the noncatalytic domain of histone deacetylases 4 and 5. Proceedings of the National Academy of Sciences of the United States of America 97: 1056-61.

57. Imayoshi I, Sakamoto M, Yamaguchi M, Mori K, Kageyama R (2010) Essential roles of Notch signaling in maintenance of neural stem cells in developing and adult brains. J Neurosci 30: 3489-98.

58. Castella P, Sawai S, Nakao K, Wagner JA, Caudy M (2000) HES-1 repression of differentiation and proliferation in PC12 cells: role for the helix 3-helix 4 domain in transcription repression. Molecular and Cellular Biology 20: 6170-83.

59. McLarren KW, Theriault FM, Stifani S (2001) Association with the nuclear matrix and interaction with Groucho and RUNX proteins regulate the transcription repression activity of the basic helix loop helix factor Hes1. The Journal of biological chemistry 276: 1578-84.

60. Gao X, Chandra T, Gratton MO, Quelo I, Prud'homme J, et al. (2001) HES6 acts as a transcriptional repressor in myoblasts and can induce the myogenic differentiation program. The Journal of cell biology 154: 1161-71.

61. Gratton MO, Torban E, Jasmin SB, Theriault FM, German MS, et al. (2003) Hes6 promotes cortical neurogenesis and inhibits Hes1 transcription repression activity by multiple mechanisms. Molecular and Cellular Biology 23: 6922-35.

62. Choi CY, Kim YH, Kwon HJ, Kim Y (1999) The homeodomain protein NK-3 recruits Groucho and a histone deacetylase complex to repress transcription. The Journal of biological chemistry 274: 33194-7.

63. Wiggins AK, Wei G, Doxakis E, Wong C, Tang AA, et al. (2004) Interaction of Brn3a and HIPK2 mediates transcriptional repression of sensory neuron survival. The Journal of cell biology 167: 257-67.

64. Ahmad A, Takami Y, Nakayama T (2003) WD dipeptide motifs and LXXLL motif of chicken HIRA are necessary for transcription repression and the latter motif is essential for interaction with histone deacetylase-2 in vivo. Biochemical and biophysical research communications 312: 1266-72.

65. Xie Y, Zhong R, Chen C, Calderwood SK (2003) Heat shock factor 1 contains two functional domains that mediate transcriptional repression of the c-fos and c-fms genes. The Journal of biological chemistry 278: 4687-98.

66. Akerfelt M, Vihervaara A, Laiho A, Conter A, Christians ES, et al. (2010) Heat shock transcription factor 1 localizes to sex chromatin during meiotic repression. The Journal of biological chemistry 285: 34469-76.

67. Kim J, Shin S, Subramaniam M, Bruinsma E, Kim TD, et al. (2010) Histone demethylase JARID1B/KDM5B is a corepressor of TIEG1/KLF10. Biochemical and biophysical research communications 401: 412-6.

68. Zhang JS, Moncrieffe MC, Kaczynski J, Ellenrieder V, Prendergast FG, et al. (2001) A conserved alpha-helical motif mediates the interaction of Sp1-like transcriptional repressors with the corepressor mSin3A. Molecular and Cellular Biology 21: 5041-9.

69. Gohla G, Krieglstein K, Spittau B (2008) Tieg3/Klf11 induces apoptosis in OLI-neu cells and enhances the TGF-beta signaling pathway by transcriptional repression of Smad7. J Cell Biochem 104: 850-61.

70. Kaczynski J, Zhang JS, Ellenrieder V, Conley A, Duenes T, et al. (2001) The Sp1-like protein BTEB3 inhibits transcription via the basic transcription element box by interacting with mSin3A and HDAC-1 co-repressors and competing with Sp1. The Journal of biological chemistry 276: 36749-56.

71. Uchida S, Tanaka Y, Ito H, Saitoh-Ohara F, Inazawa J, et al. (2000) Transcriptional regulation of the CLC-K1 promoter by myc-associated zinc finger protein and kidney-enriched Kruppel-like factor, a novel zinc finger repressor. Molecular and Cellular Biology 20: 7319-31.

72. Turner J, Nicholas H, Bishop D, Matthews JM, Crossley M (2003) The LIM protein FHL3 binds basic Kruppel-like factor/Kruppel-like factor 3 and its co-repressor C-terminal-binding protein 2. The Journal of biological chemistry 278: 12786-95.

73. Calderon MR, Verway M, An BS, DiFeo A, Bismar TA, et al. (2012) Ligand-dependent corepressor (LCoR) recruitment by Kruppel-like factor 6 (KLF6) regulates expression of the cyclin-dependent kinase inhibitor CDKN1A gene. The Journal of biological chemistry 287: 8662-74.

74. Narla G, Heath KE, Reeves HL, Li D, Giono LE, et al. (2001) KLF6, a candidate tumor suppressor gene mutated in prostate cancer. Science 294: 2563-6.

75. Laub F, Aldabe R, Friedrich V, Jr., Ohnishi S, Yoshida T, et al. (2001) Developmental expression of mouse Kruppel-like transcription factor KLF7 suggests a potential role in neurogenesis. Developmental biology 233: 305-18.

76. van Vliet J, Turner J, Crossley M (2000) Human Kruppel-like factor 8: a CACCC-box binding protein that associates with CtBP and represses transcription. Nucleic Acids Res 28: 1955-62.

77. Imhof A, Schuierer M, Werner O, Moser M, Roth C, et al. (1999) Transcriptional regulation of the AP-2alpha promoter by BTEB-1 and AP-2rep, a novel wt-1/egr-related zinc finger repressor. Molecular and Cellular Biology 19: 194-204.

78. Trojer P, Li G, Sims RJ, 3rd, Vaquero A, Kalakonda N, et al. (2007) L3MBTL1, a histone-methylation-dependent chromatin lock. Cell 129: 915-28.

79. Kalakonda N, Fischle W, Boccuni P, Gurvich N, Hoya-Arias R, et al. (2008) Histone H4 lysine 20 monomethylation promotes transcriptional repression by L3MBTL1. Oncogene 27: 4293-304.

80. Gu P, Le Menuet D, Chung AC, Cooney AJ (2009) Differential recruitment of methylated CpG binding domains by the orphan receptor GCNF initiates the repression and silencing of Oct4 expression. Molecular and Cellular Biology 29: 1987.

81. Bartholomew C, Kilbey A, Clark AM, Walker M (1997) The Evi-1 proto-oncogene encodes a transcriptional repressor activity associated with transformation. Oncogene 14: 569-77.

82. Izutsu K, Kurokawa M, Imai Y, Maki K, Mitani K, et al. (2001) The corepressor CtBP interacts with Evi-1 to repress transforming growth factor beta signaling. Blood 97: 2815-22.

83. Cattaneo F, Nucifora G (2008) EVI1 recruits the histone methyltransferase SUV39H1 for transcription repression. J Cell Biochem 105: 344-52.

84. Hurlin PJ, Queva C, Eisenman RN (1997) Mnt, a novel Max-interacting protein is coexpressed with Myc in proliferating cells and mediates repression at Myc binding sites. Genes & Development 11: 44-58.

85. Popov N, Wahlstrom T, Hurlin PJ, Henriksson M (2005) Mnt transcriptional repressor is functionally regulated during cell cycle progression. Oncogene 24: 8326-37.

86. Mazumdar A, Wang RA, Mishra SK, Adam L, Bagheri-Yarmand R, et al. (2001) Transcriptional repression of oestrogen receptor by metastasis-associated protein 1 corepressor. Nature Cell Biology 3: 30-7.

87. Roche AE, Bassett BJ, Samant SA, Hong W, Blobel GA, et al. (2008) The zinc finger and C-terminal domains of MTA proteins are required for FOG-2-mediated transcriptional repression via the NuRD complex. Journal of molecular and cellular cardiology 44: 352-60.

88. Fujita N, Jaye DL, Geigerman C, Akyildiz A, Mooney MR, et al. (2004) MTA3 and the Mi-2/NuRD complex regulate cell fate during B lymphocyte differentiation. Cell 119: 75-86.

89. Hermanson O, Jepsen K, Rosenfeld MG (2002) N-CoR controls differentiation of neural stem cells into astrocytes. Nature 419: 934-9.

90. Jepsen K, Solum D, Zhou T, McEvilly RJ, Kim HJ, et al. (2007) SMRT-mediated repression of an H3K27 demethylase in progression from neural stem cell to neuron. Nature 450: 415-9.

91. Belikov S, Astrand C, Holmqvist PH, Wrange O (2004) Chromatin-mediated restriction of nuclear factor 1/CTF binding in a repressed and hormone-activated promoter in vivo. Molecular and Cellular Biology 24: 3036-47.

92. Morel Y, Coumoul X, Nalpas A, Barouki R (2000) Nuclear factor I/CCAAT box transcription factor trans-activating domain is a negative sensor of cellular stress. Mol Pharmacol 58: 1239-46.

93. Qian F, Kruse U, Lichter P, Sippel AE (1995) Chromosomal localization of the four genes (NFIA, B, C, and X) for the human transcription factor nuclear factor I by FISH. Genomics 28: 66-73.

94. Campbell CE, Piper M, Plachez C, Yeh YT, Baizer JS, et al. (2008) The transcription factor Nfix is essential for normal brain development. BMC developmental biology 8: 52.

95. Singh SK, Wilczynska KM, Grzybowski A, Yester J, Osrah B, et al. (2011) The unique transcriptional activation domain of nuclear factor-I-X3 is critical to specifically induce marker gene expression in astrocytes. The Journal of biological chemistry 286: 7315-26.

96. Niakan KK, Davis EC, Clipsham RC, Jiang M, Dehart DB, et al. (2006) Novel role for the orphan nuclear receptor Dax1 in embryogenesis, different from steroidogenesis. Mol Genet Metab 88: 261-71.

97. Sun C, Nakatake Y, Akagi T, Ura H, Matsuda T, et al. (2009) Dax1 Binds to Oct3/4 and Inhibits Its Transcriptional Activity in Embryonic Stem Cells. Molecular and Cellular Biology 29: 4574-83.

98. Zentner MD, Lin HH, Deng HT, Kim KJ, Shih HM, et al. (2001) Requirement for high mobility group protein HMGI-C interaction with STAT3 inhibitor PIAS3 in repression of alpha-subunit of epithelial Na+ channel (alpha-ENaC) transcription by Ras activation in salivary epithelial cells. The Journal of biological chemistry 276: 29805-14.

99. Nakagawa K, Yokosawa H (2002) PIAS3 induces SUMO-1 modification and transcriptional repression of IRF-1. FEBS Lett 530: 204-8.

100. Sachdev S, Bruhn L, Sieber H, Pichler A, Melchior F, et al. (2001) PIASy, a nuclear matrix-associated SUMO E3 ligase, represses LEF1 activity by sequestration into nuclear bodies. Genes & Development 15: 3088-103.

101. Long J, Matsuura I, He D, Wang G, Shuai K, et al. (2003) Repression of Smad transcriptional activity by PIASy, an inhibitor of activated STAT. Proceedings of the National Academy of Sciences of the United States of America 100: 9791-6.

102. Gross M, Yang R, Top I, Gasper C, Shuai K (2004) PIASy-mediated repression of the androgen receptor is independent of sumoylation. Oncogene 23: 3059-66.

103. Yeap LS, Hayashi K, Surani MA (2009) ERG-associated protein with SET domain (ESET)-Oct4 interaction regulates pluripotency and represses the trophectoderm lineage. Epigenetics Chromatin 7;2(1):12.

104. Zamanian M, La Thangue NB (1993) Transcriptional repression by the Rb-related protein p107. Mol Biol Cell 4: 389-96.

105. Burkhart DL, Wirt SE, Zmoos AF, Kareta MS, Sage J (2010) Tandem E2F binding sites in the promoter of the p107 cell cycle regulator control p107 expression and its cellular functions. PLoS Genet 6: e1001003.

106. Andres ME, Burger C, Peral-Rubio MJ, Battaglioli E, Anderson ME, et al. (1999) CoREST: a functional corepressor required for regulation of neural-specific gene expression. Proceedings of the National Academy of Sciences of the United States of America 96: 9873-8.

107. Lunyak VV, Burgess R, Prefontaine GG, Nelson C, Sze SH, et al. (2002) Corepressor-dependent silencing of chromosomal regions encoding neuronal genes. Science 298: 1747-52.

108. Ouyang J, Shi Y, Valin A, Xuan Y, Gill G (2009) Direct binding of CoREST1 to SUMO-2/3 contributes to gene-specific repression by the LSD1/CoREST1/HDAC complex. Mol Cell 34: 145-54.

109. Hildebrand D, Tiefenbach J, Heinzel T, Grez M, Maurer AB (2001) Multiple regions of ETO cooperate in transcriptional repression. The Journal of biological chemistry 276: 9889-95.

110. Lausen J, Cho S, Liu S, Werner MH (2004) The nuclear receptor co-repressor (N-CoR) utilizes repression domains I and III for interaction and co-repression with ETO. The Journal of biological chemistry 279: 49281-8.

111. Salat D, Liefke R, Wiedenmann J, Borggrefe T, Oswald F (2008) ETO, but not leukemogenic fusion protein AML1/ETO, augments RBP-Jkappa/SHARP-mediated repression of notch target genes. Molecular and Cellular Biology 28: 3502-12.

112. Garcia E, Marcos-Gutierrez C, del Mar Lorente M, Moreno JC, Vidal M (1999) RYBP, a new repressor protein that interacts with components of the mammalian Polycomb complex, and with the transcription factor YY1. Embo J 18: 3404-18.

113. Hisada K, Sanchez C, Endo TA, Endoh M, Roman-Trufero M, et al. (2012) RYBP represses endogenous retroviruses and preimplantation- and germ line-specific genes in mouse embryonic stem cells. Molecular and Cellular Biology 32: 1139-49.

114. El Kasmi KC, Smith AM, Williams L, Neale G, Panopoulos AD, et al. (2007) Cutting edge: A transcriptional repressor and corepressor induced by the STAT3-regulated anti-inflammatory signaling pathway. J Immunol 179: 7215-9.

115. Alland L, Muhle R, Hou H, Jr., Potes J, Chin L, et al. (1997) Role for N-CoR and histone deacetylase in Sin3-mediated transcriptional repression. Nature 387: 49-55.

116. Naruse Y, Aoki T, Kojima T, Mori N (1999) Neural restrictive silencer factor recruits mSin3 and histone deacetylase complex to repress neuron-specific target genes. Proceedings of the National Academy of Sciences of the United States of America 96: 13691-6.

117. Rayman JB, Takahashi Y, Indjeian VB, Dannenberg JH, Catchpole S, et al. (2002) E2F mediates cell cycle-dependent transcriptional repression in vivo by recruitment of an HDAC1/mSin3B corepressor complex. Genes & Development 16: 933-47.

118. Takata T, Ishikawa F (2003) Human Sir2-related protein SIRT1 associates with the bHLH repressors HES1 and HEY2 and is involved in HES1- and HEY2-mediated transcriptional repression. Biochemical and biophysical research communications 301: 250-7.

119. Bouras T, Fu M, Sauve AA, Wang F, Quong AA, et al. (2005) SIRT1 deacetylation and repression of p300 involves lysine residues 1020/1024 within the cell cycle regulatory domain 1. The Journal of biological chemistry 280: 10264-76.

120. Zhang Q, Wang SY, Fleuriel C, Leprince D, Rocheleau JV, et al. (2007) Metabolic regulation of SIRT1 transcription via a HIC1:CtBP corepressor complex. Proceedings of the National Academy of Sciences of the United States of America 104: 829-33.

121. Binda O, Nassif C, Branton PE (2008) SIRT1 negatively regulates HDAC1-dependent transcriptional repression by the RBP1 family of proteins. Oncogene 27: 3384-92.

122. Nicol R, Stavnezer E (1998) Transcriptional repression by v-Ski and c-Ski mediated by a specific DNA binding site. The Journal of biological chemistry 273: 3588-97.

123. Cohen SB, Nicol R, Stavnezer E (1998) A domain necessary for the transforming activity of SnoN is required for specific DNA binding, transcriptional repression and interaction with TAF(II)110. Oncogene 17: 2505-13.

124. Nomura T, Khan MM, Kaul SC, Dong HD, Wadhwa R, et al. (1999) Ski is a component of the histone deacetylase complex required for transcriptional repression by Mad and thyroid hormone receptor. Genes & Development 13: 412-23.

125. Luo K, Stroschein SL, Wang W, Chen D, Martens E, et al. (1999) The Ski oncoprotein interacts with the Smad proteins to repress TGFbeta signaling. Genes & Development 13: 2196-206.

126. Wu H, D'Alessio AC, Ito S, Xia K, Wang Z, et al. (2011) Dual functions of Tet1 in transcriptional regulation in mouse embryonic stem cells. Nature 473: 389-93.

127. Williams K, Christensen J, Pedersen MT, Johansen JV, Cloos PA, et al. (2011) TET1 and hydroxymethylcytosine in transcription and DNA methylation fidelity. Nature 473: 343-8.

128. Macfarlan T, Kutney S, Altman B, Montross R, Yu J, et al. (2005) Human THAP7 is a chromatin-associated, histone tail-binding protein that represses transcription via recruitment of HDAC3 and nuclear hormone receptor corepressor. The Journal of biological chemistry 280: 7346-58.

129. Macfarlan T, Parker JB, Nagata K, Chakravarti D (2006) Thanatos-associated protein 7 associates with template activating factor-Ibeta and inhibits histone acetylation to repress transcription. Mol Endocrinol 20: 335-47.

130. Levanon D, Goldstein RE, Bernstein Y, Tang H, Goldenberg D, et al. (1998) Transcriptional repression by AML1 and LEF-1 is mediated by the TLE/Groucho corepressors. Proceedings of the National Academy of Sciences of the United States of America 95: 11590-5.

131. Ren B, Chee KJ, Kim TH, Maniatis T (1999) PRDI-BF1/Blimp-1 repression is mediated by corepressors of the Groucho family of proteins. Genes & Development 13: 125-37.

132. Dasen JS, Barbera JP, Herman TS, Connell SO, Olson L, et al. (2001) Temporal regulation of a paired-like homeodomain repressor/TLE corepressor complex and a related activator is required for pituitary organogenesis. Genes & Development 15: 3193-207.

133. Ali SA, Zaidi SK, Dobson JR, Shakoori AR, Lian JB, et al. (2010) Transcriptional corepressor TLE1 functions with Runx2 in epigenetic repression of ribosomal RNA genes. Proceedings of the National Academy of Sciences of the United States of America 107: 4165-9.

134. Villanueva CJ, Waki H, Godio C, Nielsen R, Chou WL, et al. (2011) TLE3 is a dual-function transcriptional coregulator of adipogenesis. Cell Metab 13: 413-27.

135. Burks PJ, Isaacs HV, Pownall ME (2009) FGF signalling modulates transcriptional repression by Xenopus groucho-related-4. Biol Cell 101: 301-8.

136. Patel SR, Bhumbra SS, Paknikar RS, Dressler GR (2012) Epigenetic mechanisms of Groucho/Grg/TLE mediated transcriptional repression. Mol Cell 45: 185-95.

137. Zhu CC, Dyer MA, Uchikawa M, Kondoh H, Lagutin OV, et al. (2002) Six3-mediated auto repression and eye development requires its interaction with members of the Groucho-related family of co-repressors. Development 129: 2835-49.

138. Marcal N, Patel H, Dong Z, Belanger-Jasmin S, Hoffman B, et al. (2005) Antagonistic effects of Grg6 and Groucho/TLE on the transcription repression activity of brain factor 1/FoxG1 and cortical neuron differentiation. Molecular and Cellular Biology 25: 10916-29.

139. Grbavec D, Lo R, Liu Y, Greenfield A, Stifani S (1999) Groucho/transducin-like enhancer of split (TLE) family members interact with the yeast transcriptional co-repressor SSN6 and mammalian SSN6-related proteins: implications for evolutionary conservation of transcription repression mechanisms. Biochem J 337 (Pt 1): 13-7.

140. Garcia-Tunon I, Guallar D, Alonso-Martin S, Benito AA, Benitez-Lazaro A, et al. (2011) Association of Rex-1 to target genes supports its interaction with Polycomb function. Stem Cell Res 7: 1-16.

141. O'Carroll D, Erhardt S, Pagani M, Barton SC, Surani MA, et al. (2001) The polycomb-group gene Ezh2 is required for early mouse development. Mol Cell Biol 21: 4330-6.

142. Kim SH, Park J, Choi MC, Kim HP, Park JH, et al. (2007) Zinc-fingers and homeoboxes 1 (ZHX1) binds DNA methyltransferase (DNMT) 3B to enhance DNMT3B-mediated

transcriptional repression. Biochemical and biophysical research communications 355: 318-23.

143. Yan W, Si Y, Slaymaker S, Li J, Zheng H, et al. (2010) Zmynd15 encodes a histone deacetylase-dependent transcriptional repressor essential for spermiogenesis and male fertility. The Journal of biological chemistry 285: 31418-26.
