# Supplementary material for: Genome-Wide Analysis Reveals the Unique Stem Cell Identity of Human Amniocytes
Source: PLoS One. 2013 Jan 10;8(1):e53372. doi: 10.1371/journal.pone.0053372 (PMC3542377; doi:10.1371/journal.pone.0053372)
Supplement: Table S5 — Reference list of 150 genes for primary embryonic lineages. The 150 genes were included in our list based on the criteria; 1) previous reports show the gene playing a possible functional role in any of the three primary germ layers or in the trophectodermal lineage during development and 2) each gene was reliably detected at significant levels in our RNA-seq dataset. (PDF) [file pone.0053372.s006.pdf]

Table S5. Reference list of 150 genes for primary embryonic lineages. The 150 genes were included in our list based on the criteria; 1) previous reports show the gene playing a possible functional role in any of the three primary germ layers or in the trophectodermal lineage during development and 2) each gene was reliably detected at significant levels in our RNA-seq dataset.

Gene_ Symbol	Ensgene_id	Refseq_id	Lineage	References
Egr2	ENSG00000122877	NM_000399	Ectoderm	[1,2]
Fgf5	ENSG00000138675	NM_004464	Ectoderm	[3,4]
Foxj3	ENSG00000198815	NM_001198850	Ectoderm	[5]
Gbx2	ENSG00000168505	NM_001485.2	Ectoderm	[4,6]
Lhx5	ENSG00000089116	NM_022363	Ectoderm	[7]
Lmx1a	ENSG00000162761	NM_001033507	Ectoderm	[8]
Meis1	ENSG00000143995	NM_002398	Ectoderm	[9,10]
Meis2	ENSG00000134138	NM_170677	Ectoderm	[9,10]
Nes	ENSG00000132688	NM_006617	Ectoderm	[11]
Pard6b	ENSG00000124171	NM_032521	Ectoderm	[12]
Pax2	ENSG00000075891	NM_000278	Ectoderm	[13-16]
Pax6	ENSG00000007372	NM_000280	Ectoderm	[17-19]
Penk	ENSG00000181195	NM_001135690	Ectoderm	[12]
Rbm27	ENSG00000091009	XM_291128	Ectoderm	[3]
Sox1	ENSG00000182968	NM_005986	Ectoderm	[20]
Tfcp2l1	ENSG00000115112	NM_014553	Ectoderm	[3]
Trim33	ENSG00000197323	NM_015906.3	Ectoderm	[21]
Tubb3	ENSG00000198211	NM_006086	Ectoderm	[22]
Zic1	ENSG00000152977	NM_003412	Ectoderm	[23]
Afp	ENSG00000081051	NM_001134	Endoderm	[24,25]
Calcr	ENSG00000004948	NM_001164737.1	Endoderm	[26,27]
Cckbr	ENSG00000110148	NM_176875.3	Endoderm	[26]
Cer1	ENSG00000147869	NM_005454	Endoderm	[27]
Cxcr4	ENSG00000121966	NM_001008540	Endoderm	[27-30]
Cyp26a1	ENSG00000095596	NM_000783.3	Endoderm	[26]
Dab2	ENSG00000153071	NM_001343	Endoderm	[31,32]
Dkk4	ENSG00000104371	NM_014420	Endoderm	[27]
Dlx3	ENSG00000064195	NM_005220	Endoderm	[33]
Dlx5	ENSG00000105880	NM_005221	Endoderm	[33]
Elf4	ENSG00000102034	NM_001421	Endoderm	[27]
Eya2	ENSG00000064655	NM_005244	Endoderm	[27]

Fgf17	ENSG00000158815	NM_003867.2	Endoderm	[26,27]
Foxa2	ENSG00000125798	NM_153675	Endoderm	[26,34,35]
Foxa3	ENSG00000170608	NM_004497	Endoderm	[35,36]
Foxc1	ENSG00000054598	NM_001453	Endoderm	[27]
Foxg1	ENSG00000176165	NM_005249	Endoderm	[33]
Foxh1	ENSG00000160973	NM_003923	Endoderm	[37,38]
Foxq1	ENSG00000164379	NM_033260	Endoderm	[27]
Gata6	ENSG00000141448	NM_005257	Endoderm	[27,39-42]
Gpc1	ENSG00000063660	NM_002081	Endoderm	[30,43]
Hhex	ENSG00000152804	NM_002729	Endoderm	[27,44]
Id4	ENSG00000172201	NM_001546	Endoderm	[33]
Krt19	ENSG00000171345	NM_002276	Endoderm	[45]
Nts	ENSG00000133636	NM_006183	Endoderm	[27]
Onecut1	ENSG00000169856	NM_004498	Mixed Lineages	[46-48]
Pax9	ENSG00000198807	NM_006194	Endoderm	[33]
Plxna2	ENSG00000076356	NM_025179	Endoderm	[27]
Prdm1	ENSG00000057657	NM_001198.3	Endoderm	[26,49]
Pyy	ENSG00000131096	NM_004160	Endoderm	[50]
Shisa2	ENSG00000180730	NM_001007538	Endoderm	[26,51]
Sox17	ENSG00000164736	NM_022454	Endoderm	[52,53]
Sox7	ENSG00000171056	NM_031439	Endoderm	[54,55]
Sp6	ENSG00000189120	NM_199262	Endoderm	[33]
Tle2	ENSG00000065717	NM_003260	Endoderm	[27]
Trim22	ENSG00000132274	NM_006074	Endoderm	[27]
Tspan7	ENSG00000156298	NM_004615	Endoderm	[30,56,57]
Cxcl12	ENSG00000107562	NM_000609	Mesoderm	[28,29]
Ednrb	ENSG00000136160	NM_000115	Mesoderm	[58]
Foxf1	ENSG00000103241	NM_001451	Mesoderm	[59-61]
Lhx1	ENSG00000132130	NM_005568	Mesoderm	[62-64]
Lmo2	ENSG00000135363	NM_001142315.1	Mesoderm	[26]
Meox1	ENSG00000005102	NM_001040002	Mesoderm	[65,66]
Meox2	ENSG00000106511	NM_005924	Mesoderm	[65,66]
Mesdc2	ENSG00000117899	NM_015154	Mesoderm	[67]
Msx1	ENSG00000163132	NM_002448	Mesoderm	[68-70]
Msx2	ENSG00000120149	NM_002449	Mesoderm	[70,71]
Myl4	ENSG00000198336	NM_001002841	Mesoderm	[72]
Myocd	ENSG00000141052	NM_153604	Mesoderm	[73]
Pbx1	ENSG00000185630	NM_001204961	Mesoderm	[74-76]
Ror2	ENSG00000169071	NM_004560	Mesoderm	[77,78]
Sox6	ENSG00000110693	NM_001145819	Mesoderm	[79,80]
Tbx6	ENSG00000149922	NM_004608	Mesoderm	[81-85]

Tcf15	ENSG00000125878	NM_004609	Mesoderm	[86]
Wnt5a	ENSG00000114251	NM_001256105.1	Mesoderm	[87,88]
Wnt5b	ENSG00000111186	NM_030775.2	Mesoderm	[89]
Wnt8a	ENSG00000061492	NM_058244	Mesoderm	[90-92]
Anxa4	ENSG00000196975	NM_001153	Mixed Lineages	[45]
Bmp2	ENSG00000125845	NM_001200	Mixed Lineages	[93-96]
Bmp4	ENSG00000125378	NM_001202	Mixed Lineages	[93,97,98]
Cdx2	ENSG00000165556	NM_001265	Mixed Lineages	[99-101]
Dkk1	ENSG00000107984	NM_012242	Mixed Lineages	[102-105]
Dsg2	ENSG00000046604	NM_001943	Mixed Lineages	[45]
Eomes	ENSG00000163508	NM_005442	Mixed Lineages	[99,106]
Fgf8	ENSG00000107831	NM_001206389.1	Mixed Lineages	[107-109]
Foxa1	ENSG00000129514	NM_004496	Mixed Lineages	[34,110,111]
Gata3	ENSG00000107485	NM_002051	Mixed Lineages	[112-114]
Gata4	ENSG00000136574	NM_002052	Mixed Lineages	[60,115-118]
Gdf3	ENSG00000184344	NM_020634	Mixed Lineages	[119,120]
Gsc	ENSG00000133937	NM_173849	Mixed Lineages	[30,121-125]
Hand1	ENSG00000113196	NM_004821	Mixed Lineages	[126-129]
Hand2	ENSG00000164107	NM_021973	Mixed Lineages	[127,130-132]
Hnf4a	ENSG00000101076	NM_001030003	Mixed Lineages	[133,134]
Hox1a	ENSG00000105991	NM_005522	Mixed Lineages	[135-137]
Hoxb1	ENSG00000120094	NM_002144	Mixed Lineages	[137-139]
Isl1	ENSG00000016082	NM_002202	Mixed Lineages	[140-142]
Kdr	ENSG00000128052	NM_002253	Mixed Lineages	[143,144]
Mesp1	ENSG00000166823	NM_018670	Mixed Lineages	[145]
Mesp2	ENSG00000188095	NM_001039958	Mixed Lineages	[146]

Mixl1	ENSG00000185155	NM_031944	Mixed Lineages	[147,148]
Otx1	ENSG00000115507	NM_001199770	Mixed Lineages	[13,149,150]
Otx2	ENSG00000165588	NM_021728.2	Mixed Lineages	[13,149]
Pdgfra	ENSG00000134853	NM_006206	Mixed Lineages	[42,151-153]
Pdgfrb	ENSG00000113721	NM_002609	Mixed Lineages	[154,155]
Ripk4	ENSG00000183421	NM_020639	Mixed Lineages	[45]
T	ENSG00000164458	NM_003181	Mixed Lineages	[81,156-161]
Wnt3a	ENSG00000154342	NM_033131	Mixed Lineages	[81,85,162]
Akr1b1	ENSG00000085662	NM_001628	Trophectoderm	[163]
Ascl2	ENSG00000183734	NM_005170	Trophectoderm	[164-166]
Cebpb	ENSG00000172216	NM_005194	Trophectoderm	[167]
Cgb	ENSG00000104827	NM_000737	Trophectoderm	[168]
Cgb1	ENSG00000204748	NM_033377	Trophectoderm	[169]
Cgb2	ENSG00000104818	NM_033378	Trophectoderm	[169]
Cgb5	ENSG00000189052	NM_033043	Trophectoderm	[170,171]
Cgb7	ENSG00000196337	NM_033142	Trophectoderm	[170]
Cgb8	ENSG00000213030	NM_033183	Trophectoderm	[170]
Csh1	ENSG00000136488	NM_001317	Trophectoderm	[165,172]
Csh2	ENSG00000213218	NM_020991	Trophectoderm	[173]
Ctsc	ENSG00000109861	NM_001814	Trophectoderm	[77]
Cyp19a1	ENSG00000137869	NM_000103	Trophectoderm	[77,174]
Elf5	ENSG00000135374	NM_001422	Trophectoderm	[175-178]
Enpep	ENSG00000138792	NM_001977	Trophectoderm	[77]
Esx1	ENSG00000123576	NM_153448	Trophectoderm	[179]
Ets2	ENSG00000157557	NM_001256295	Trophectoderm	[180,181]
Fgfr1	ENSG00000077782	NM_023108	Trophectoderm	[182]
Gcm1	ENSG00000137270	NM_003643	Trophectoderm	[183,184]
H19	ENSG00000130600	NR_002196	Trophectoderm	[185]
Krt7	ENSG00000135480	NM_005556	Trophectoderm	[186]
Krt8	ENSG00000170421	NM_002273	Trophectoderm	[187]
Lhb	ENSG00000104826	NM_000894	Trophectoderm	[188]
Lhcgr	ENSG00000138039	NM_000233	Trophectoderm	[189]
Lifr	ENSG00000113594	NM_001127671	Trophectoderm	[190]
Plac1	ENSG00000170965	NM_021796	Trophectoderm	[191,192]
Plac2	ENSG00000223573	NR_027064	Trophectoderm	[77]
Psg1	ENSG00000231924	NM_006905	Trophectoderm	[193,194]

Psg11	ENSG00000243130	NM_203287	Trophectoderm	[193]
Psg2	ENSG00000242221	NM_031246	Trophectoderm	[193]
Psg3	ENSG00000221826	NM_021016	Trophectoderm	[194]
Psg4	ENSG00000243137	NM_213633	Trophectoderm	[195]
Psg5	ENSG00000204941	NM_001130014	Trophectoderm	[194]
Psg6	ENSG00000170848	NM_002782	Trophectoderm	[196,197]
Psg7	ENSG00000221878	NM_001206650	Trophectoderm	[194]
Psg8	ENSG00000124467	NM_001130167	Trophectoderm	[198]
Psg9	ENSG00000183668	NM_002784	Trophectoderm	[198]
Rsl1d1	ENSG00000171490	NM_015659	Trophectoderm	[199]
Stra13	ENSG00000169689	NM_144998	Trophectoderm	[200]
Tead4	ENSG00000197905	NM_003213	Trophectoderm	[201-203]
Tfap2c	ENSG00000087510	NM_003222	Trophectoderm	[204]
Thbd	ENSG00000178726	NM_000361	Trophectoderm	[205,206]
Vtcn1	ENSG00000134258	NM_024626	Trophectoderm	[77]

REFERENCES

1. Lee EK, Bae GU, You JS, Lee JC, Jeon YJ, et al. (2009) Reversine increases the plasticity of lineage-committed cells toward neuroectodermal lineage. The Journal of biological chemistry 284: 2891-901.

2. Sun Z, Zhao J, Zhang Y, Meng A (2006) Sp5l is a mediator of Fgf signals in anteroposterior patterning of the neuroectoderm in zebrafish embryo. Developmental dynamics : an official publication of the American Association of Anatomists 235: 2999-3006.

3. Pelton TA, Sharma S, Schulz TC, Rathjen J, Rathjen PD (2002) Transient pluripotent cell populations during primitive ectoderm formation: correlation of in vivo and in vitro pluripotent cell development. J Cell Sci 115: 329-39.

4. Rathjen J, Lake JA, Bettess MD, Washington JM, Chapman G, et al. (1999) Formation of a primitive ectoderm like cell population, EPL cells, from ES cells in response to biologically derived factors. J Cell Sci 112 (Pt 5): 601-12.

5. Landgren H, Carlsson P (2004) FoxJ3, a novel mammalian forkhead gene expressed in neuroectoderm, neural crest, and myotome. Developmental dynamics : an official publication of the American Association of Anatomists 231: 396-401.

6. Kimura C, Shen MM, Takeda N, Aizawa S, Matsuo I (2001) Complementary functions of Otx2 and Cripto in initial patterning of mouse epiblast. Developmental biology 235: 12-32.

7. Toyama R, Curtiss PE, Otani H, Kimura M, Dawid IB, et al. (1995) The LIM class homeobox gene lim5: implied role in CNS patterning in Xenopus and zebrafish. Developmental biology 170: 583-93.

8. Chizhikov VV, Millen KJ (2004) Control of roof plate formation by Lmx1a in the developing spinal cord. Development 131: 2693-705.

9. Mercader N, Leonardo E, Piedra ME, Martinez AC, Ros MA, et al. (2000) Opposing RA and FGF signals control proximodistal vertebrate limb development through regulation of Meis genes. Development 127: 3961-70.

10. Zhang X, Friedman A, Heaney S, Purcell P, Maas RL (2002) Meis homeoproteins directly regulate Pax6 during vertebrate lens morphogenesis. Genes & Development 16: 2097-107.

11. Dubois NC, Hofmann D, Kaloulis K, Bishop JM, Trumpp A (2006) Nestin-Cre transgenic mouse line Nes-Cre1 mediates highly efficient Cre/loxP mediated recombination in the nervous system, kidney, and somite-derived tissues. Genesis 44: 355-60.

12. Harvey NT, Hughes JN, Lonic A, Yap C, Long C, et al. (2010) Response to BMP4 signalling during ES cell differentiation defines intermediates of the ectoderm lineage. J Cell Sci 123: 1796-804.

13. Acampora D, Annino A, Puelles E, Alfano I, Tuorto F, et al. (2003) OTX1 compensates for OTX2 requirement in regionalisation of anterior neuroectoderm. Gene Expr Patterns 3: 497-501.

14. Bovolenta P, Mallamaci A, Briata P, Corte G, Boncinelli E (1997) Implication of OTX2 in pigment epithelium determination and neural retina differentiation. J Neurosci 17: 4243-52.

15. Lun K, Brand M (1998) A series of no isthmus (noi) alleles of the zebrafish pax2.1 gene reveals multiple signaling events in development of the midbrain-hindbrain boundary. Development 125: 3049-62.

16. Rhinn M, Dierich A, Shawlot W, Behringer RR, Le Meur M, et al. (1998) Sequential roles for Otx2 in visceral endoderm and neuroectoderm for forebrain and midbrain induction and specification. Development 125: 845-56.

17. Grindley JC, Davidson DR, Hill RE (1995) The role of Pax-6 in eye and nasal development. Development 121: 1433-42.

18. Li HS, Yang JM, Jacobson RD, Pasko D, Sundin O (1994) Pax-6 is first expressed in a region of ectoderm anterior to the early neural plate: implications for stepwise determination of the lens. Developmental biology 162: 181-94.

19. Quinn JC, West JD, Hill RE (1996) Multiple functions for Pax6 in mouse eye and nasal development. Genes & Development 10: 435-46.

20. Pevny LH, Sockanathan S, Placzek M, Lovell-Badge R (1998) A role for SOX1 in neural determination. Development 125: 1967-78.

21. Morsut L, Yan KP, Enzo E, Aragona M, Soligo SM, et al. (2010) Negative control of Smad activity by ectodermin/Tif1gamma patterns the mammalian embryo. Development 137: 2571-8.

22. Anokye-Danso F, Trivedi CM, Juhr D, Gupta M, Cui Z, et al. (2011) Highly efficient miRNA-mediated reprogramming of mouse and human somatic cells to pluripotency. Cell Stem Cell 8: 376-88.

23. Nagai T, Aruga J, Takada S, Gunther T, Sporle R, et al. (1997) The expression of the mouse Zic1, Zic2, and Zic3 gene suggests an essential role for Zic genes in body pattern formation. Developmental biology 182: 299-313.

24. Jones EA, Clement-Jones M, James OF, Wilson DI (2001) Differences between human and mouse alpha-fetoprotein expression during early development. Journal of anatomy 198: 555-9.

25. Kwon GS, Fraser ST, Eakin GS, Mangano M, Isern J, et al. (2006) Tg(Afp-GFP) expression marks primitive and definitive endoderm lineages during mouse development. Developmental dynamics : an official publication of the American Association of Anatomists 235: 2549-58.

26. Bernardo Andreia S, Faial T, Gardner L, Niakan Kathy K, Ortmann D, et al. (2011) BRACHYURY and CDX2 Mediate BMP-Induced Differentiation of Human and Mouse Pluripotent Stem Cells into Embryonic and Extraembryonic Lineages. Cell Stem Cell 9: 144-55.

27. McLean AB, D'Amour KA, Jones KL, Krishnamoorthy M, Kulik MJ, et al. (2007) Activin a efficiently specifies definitive endoderm from human embryonic stem cells only when phosphatidylinositol 3-kinase signaling is suppressed. Stem Cells 25: 29-38.

28. D'Amour KA, Agulnick AD, Eliazer S, Kelly OG, Kroon E, et al. (2005) Efficient differentiation of human embryonic stem cells to definitive endoderm. Nature Biotechnology 23: 1534-41.

29. McGrath KE, Koniski AD, Maltby KM, McGann JK, Palis J (1999) Embryonic expression and function of the chemokine SDF-1 and its receptor, CXCR4. Developmental biology 213: 442-56.

30. Yasunaga M, Tada S, Torikai-Nishikawa S, Nakano Y, Okada M, et al. (2005) Induction and monitoring of definitive and visceral endoderm differentiation of mouse ES cells. Nature Biotechnology 23: 1542-50.

31. Niakan KK, Ji H, Maehr R, Vokes SA, Rodolfa KT, et al. (2010) Sox17 promotes differentiation in mouse embryonic stem cells by directly regulating extraembryonic gene expression and indirectly antagonizing self-renewal. Genes & Development 24: 312-26.

32. Morrisey EE, Musco S, Chen MY, Lu MM, Leiden JM, et al. (2000) The gene encoding the mitogen-responsive phosphoprotein Dab2 is differentially regulated by GATA-6 and GATA-4 in the visceral endoderm. The Journal of biological chemistry 275: 19949-54.

33. Sherwood RI, Jitianu C, Cleaver O, Shaywitz DA, Lamenzo JO, et al. (2007) Prospective isolation and global gene expression analysis of definitive and visceral endoderm. Developmental biology 304: 541-55.

34. Ang SL, Wierda A, Wong D, Stevens KA, Cascio S, et al. (1993) The formation and maintenance of the definitive endoderm lineage in the mouse: involvement of HNF3/forkhead proteins. Development 119: 1301-15.

35. Dufort D, Schwartz L, Harpal K, Rossant J (1998) The transcription factor HNF3beta is required in visceral endoderm for normal primitive streak morphogenesis. Development 125: 3015-25.

36. Hiemisch H, Schutz G, Kaestner KH (1997) Transcriptional regulation in endoderm development: characterization of an enhancer controlling Hnf3g expression by transgenesis and targeted mutagenesis. Embo J 16: 3995-4006.

37. Hoodless PA, Pye M, Chazaud C, Labbe E, Attisano L, et al. (2001) FoxH1 (Fast) functions to specify the anterior primitive streak in the mouse. Genes & Development 15: 1257-71.

38. McKnight KD, Hou J, Hoodless PA (2010) Foxh1 and Foxa2 are not required for formation of the midgut and hindgut definitive endoderm. Developmental biology 337: 471-81.

39. Hay DC, Sutherland L, Clark J, Burdon T (2004) Oct-4 knockdown induces similar patterns of endoderm and trophoblast differentiation markers in human and mouse embryonic stem cells. Stem Cells 22: 225-35.

40. Chazaud C, Yamanaka Y, Pawson T, Rossant J (2006) Early lineage segregation between epiblast and primitive endoderm in mouse blastocysts through the Grb2-MAPK pathway. Developmental Cell 10: 615-24.

41. Li L, Arman E, Ekblom P, Edgar D, Murray P, et al. (2004) Distinct GATA6- and laminin-dependent mechanisms regulate endodermal and ectodermal embryonic stem cell fates. Development 131: 5277-86.

42. Plusa B, Piliszek A, Frankenberg S, Artus J, Hadjantonakis AK (2008) Distinct sequential cell behaviours direct primitive endoderm formation in the mouse blastocyst. Development 135: 3081-91.

43. Filmus J (2001) Glypicans in growth control and cancer. Glycobiology 11(3):19R-23R.

44. Thomas PQ, Brown A, Beddington RS (1998) Hex: a homeobox gene revealing peri-implantation asymmetry in the mouse embryo and an early transient marker of endothelial cell precursors. Development 125: 85-94.

45. Brown K, Legros S, Artus J, Doss MX, Khanin R, et al. (2010) A comparative analysis of extra-embryonic endoderm cell lines. PLoS One 5: e12016.

46. Jacquemin P, Yoshitomi H, Kashima Y, Rousseau GG, Lemaigre FP, et al. (2006) An endothelial-mesenchymal relay pathway regulates early phases of pancreas development. Developmental biology 290: 189-99.

47. Rausa F, Samadani U, Ye H, Lim L, Fletcher CF, et al. (1997) The cut-homeodomain transcriptional activator HNF-6 is coexpressed with its target gene HNF-3 beta in the developing murine liver and pancreas. Developmental biology 192: 228-46.

48. Pierreux CE, Vanhorenbeeck V, Jacquemin P, Lemaigre FP, Rousseau GG (2004) The transcription factor hepatocyte nuclear factor-6/Onecut-1 controls the expression of its paralog Onecut-3 in developing mouse endoderm. The Journal of biological chemistry 279: 51298-304.

49. Chang DH, Cattoretti G, Calame KL (2002) The dynamic expression pattern of B lymphocyte induced maturation protein-1 (Blimp-1) during mouse embryonic development. Mechanisms of Development 117: 305-9.

50. Hou J, Charters AM, Lee SC, Zhao Y, Wu MK, et al. (2007) A systematic screen for genes expressed in definitive endoderm by Serial Analysis of Gene Expression (SAGE). BMC developmental biology 7: 92.

51. Filipe M, Goncalves L, Bento M, Silva AC, Belo JA (2006) Comparative expression of mouse and chicken Shisa homologues during early development. Developmental dynamics : an official publication of the American Association of Anatomists 235: 2567-73.

52. Frankenberg S, Gerbe F, Bessonnard S, Belville C, Pouchin P, et al. (2011) Primitive endoderm differentiates via a three-step mechanism involving Nanog and RTK signaling. Developmental Cell 21: 1005-13.

53. Kanai-Azuma M, Kanai Y, Gad JM, Tajima Y, Taya C, et al. (2002) Depletion of definitive gut endoderm in Sox17-null mutant mice. Development 129: 2367-79.

54. Niimi T, Hayashi Y, Futaki S, Sekiguchi K (2004) SOX7 and SOX17 regulate the parietal endoderm-specific enhancer activity of mouse laminin alpha1 gene. The Journal of biological chemistry 279: 38055-61.

55. Futaki S, Hayashi Y, Emoto T, Weber CN, Sekiguchi K (2004) Sox7 plays crucial roles in parietal endoderm differentiation in F9 embryonal carcinoma cells through regulating Gata-4 and Gata-6 expression. Molecular and Cellular Biology 24: 10492-503.

56. Holtzinger A, Rosenfeld GE, Evans T (2010) Gata4 directs development of cardiac-inducing endoderm from ES cells. Developmental biology 337: 63-73.

57. Hemler ME (2001) Specific tetraspanin functions. The Journal of cell biology 155: 1103-7.

58. Welsh IC, O'Brien TP (2000) Loss of late primitive streak mesoderm and interruption of left-right morphogenesis in the Ednrb(s-1Acrg) mutant mouse. Developmental biology 225: 151-68.

59. Mahlapuu M, Ormestad M, Enerback S, Carlsson P (2001) The forkhead transcription factor Foxf1 is required for differentiation of extra-embryonic and lateral plate mesoderm. Development 128: 155-66.

60. Rojas A, De Val S, Heidt AB, Xu SM, Bristow J, et al. (2005) Gata4 expression in lateral mesoderm is downstream of BMP4 and is activated directly by Forkhead and GATA transcription factors through a distal enhancer element. Development 132: 3405-17.

61. Ormestad M, Astorga J, Carlsson P (2004) Differences in the embryonic expression patterns of mouse Foxf1 and -2 match their distinct mutant phenotypes. Developmental dynamics : an official publication of the American Association of Anatomists 229: 328-33.

62. Barnes JD, Crosby JL, Jones CM, Wright CV, Hogan BL (1994) Embryonic expression of Lim-1, the mouse homolog of Xenopus Xlim-1, suggests a role in lateral mesoderm differentiation and neurogenesis. Developmental biology 161: 168-78.

63. Tsang TE, Shawlot W, Kinder SJ, Kobayashi A, Kwan KM, et al. (2000) Lim1 activity is required for intermediate mesoderm differentiation in the mouse embryo. Developmental biology 223: 77-90.

64. Cirio MC, Hui Z, Haldin CE, Cosentino CC, Stuckenholz C, et al. (2011) Lhx1 is required for specification of the renal progenitor cell field. PLoS One 6: e18858.

65. Candia AF, Hu J, Crosby J, Lalley PA, Noden D, et al. (1992) Mox-1 and Mox-2 define a novel homeobox gene subfamily and are differentially expressed during early mesodermal patterning in mouse embryos. Development 116: 1123-36.

66. Candia AF, Wright CV (1996) Differential localization of Mox-1 and Mox-2 proteins indicates distinct roles during development. The International Journal of Developmental Biology 40: 1179-84.

67. Wines ME, Lee L, Katari MS, Zhang L, DeRossi C, et al. (2001) Identification of mesoderm development (mesd) candidate genes by comparative mapping and genome sequence analysis. Genomics 72: 88-98.

68. Rashbass P, Wilson V, Rosen B, Beddington RS (1994) Alterations in gene expression during mesoderm formation and axial patterning in Brachyury (T) embryos. The International Journal of Developmental Biology 38: 35-44.

69. Satokata I, Maas R (1994) Msx1 deficient mice exhibit cleft palate and abnormalities of craniofacial and tooth development. Nature Genetics 6: 348-56.

70. Bensoussan-Trigano V, Lallemand Y, Saint Cloment C, Robert B (2011) Msx1 and Msx2 in limb mesenchyme modulate digit number and identity. Developmental dynamics : an official publication of the American Association of Anatomists 240: 1190-202.

71. Liu YH, Ma L, Wu LY, Luo W, Kundu R, et al. (1994) Regulation of the Msx2 homeobox gene during mouse embryogenesis: a transgene with 439 bp of 5' flanking sequence is expressed exclusively in the apical ectodermal ridge of the developing limb. Mechanisms of Development 48: 187-97.

72. Jagtap S, Meganathan K, Gaspar J, Wagh V, Winkler J, et al. (2011) Cytosine arabinoside induces ectoderm and inhibits mesoderm expression in human embryonic stem cells during multilineage differentiation. Br J Pharmacol 162: 1743-56.

73. Han Z, Li X, Wu J, Olson EN (2004) A myocardin-related transcription factor regulates activity of serum response factor in Drosophila. Proceedings of the National Academy of Sciences of the United States of America 101: 12567-72.

74. Capellini TD, Zewdu R, Di Giacomo G, Asciutti S, Kugler JE, et al. (2008) Pbx1/Pbx2 govern axial skeletal development by controlling Polycomb and Hox in mesoderm and Pax1/Pax9 in sclerotome. Developmental biology 321: 500-14.

75. Schnabel CA, Selleri L, Jacobs Y, Warnke R, Cleary ML (2001) Expression of Pbx1b during mammalian organogenesis. Mechanisms of Development 100: 131-5.

76. Schnabel CA, Godin RE, Cleary ML (2003) Pbx1 regulates nephrogenesis and ureteric branching in the developing kidney. Developmental biology 254: 262-76.

77. Drukker M, Tang C, Ardehali R, Rinkevich Y, Seita J, et al. (2012) Isolation of primitive endoderm, mesoderm, vascular endothelial and trophoblast progenitors from human pluripotent stem cells. Nature Biotechnology 30(6):531-42

78. Hikasa H, Shibata M, Hiratani I, Taira M (2002) The Xenopus receptor tyrosine kinase Xror2 modulates morphogenetic movements of the axial mesoderm and neuroectoderm via Wnt signaling. Development 129: 5227-39.

79. Chimal-Monroy J, Rodriguez-Leon J, Montero JA, Ganan Y, Macias D, et al. (2003) Analysis of the molecular cascade responsible for mesodermal limb chondrogenesis: Sox genes and BMP signaling. Developmental biology 257: 292-301.

80. Brent AE, Braun T, Tabin CJ (2005) Genetic analysis of interactions between the somitic muscle, cartilage and tendon cell lineages during mouse development. Development 132: 515-28.

81. Yamaguchi TP, Takada S, Yoshikawa Y, Wu N, McMahon AP (1999) T (Brachyury) is a direct target of Wnt3a during paraxial mesoderm specification. Genes & Development 13: 3185-90.

82. Chapman DL, Agulnik I, Hancock S, Silver LM, Papaioannou VE (1996) Tbx6, a mouse T-Box gene implicated in paraxial mesoderm formation at gastrulation. Developmental biology 180: 534-42.

83. Chapman DL, Cooper-Morgan A, Harrelson Z, Papaioannou VE (2003) Critical role for Tbx6 in mesoderm specification in the mouse embryo. Mechanisms of Development 120: 837-47.

84. Yasuhiko Y, Kitajima S, Takahashi Y, Oginuma M, Kagiwada H, et al. (2008) Functional importance of evolutionally conserved Tbx6 binding sites in the presomitic mesoderm-specific enhancer of Mesp2. Development 135: 3511-9.

85. Nowotschin S, Ferrer-Vaquer A, Concepcion D, Papaioannou VE, Hadjantonakis AK (2012) Interaction of Wnt3a, Msgn1 and Tbx6 in neural versus paraxial mesoderm lineage commitment and paraxial mesoderm differentiation in the mouse embryo. Developmental biology 367(1):1-14.

86. Quertermous EE, Hidai H, Blanar MA, Quertermous T (1994) Cloning and characterization of a basic helix-loop-helix protein expressed in early mesoderm and the developing somites. Proceedings of the National Academy of Sciences of the United States of America 91: 7066-70.

87. Tajbakhsh S, Borello U, Vivarelli E, Kelly R, Papkoff J, et al. (1998) Differential activation of Myf5 and MyoD by different Wnts in explants of mouse paraxial mesoderm and the later activation of myogenesis in the absence of Myf5. Development 125: 4155-62.

88. Yamaguchi TP, Bradley A, McMahon AP, Jones S (1999) A Wnt5a pathway underlies outgrowth of multiple structures in the vertebrate embryo. Development 126: 1211-23.

89. Fazzi R, Pacini S, Carnicelli V, Trombi L, Montali M, et al. (2011) Mesodermal progenitor cells (MPCs) differentiate into mesenchymal stromal cells (MSCs) by activation of Wnt5/calmodulin signalling pathway. PLoS One 6: e25600.

90. Kelly GM, Greenstein P, Erezyilmaz DF, Moon RT (1995) Zebrafish wnt8 and wnt8b share a common activity but are involved in distinct developmental pathways. Development 121: 1787-99.

91. Hoppler S, Moon RT (1998) BMP-2/-4 and Wnt-8 cooperatively pattern the Xenopus mesoderm. Mechanisms of Development 71: 119-29.

92. Lekven AC, Thorpe CJ, Waxman JS, Moon RT (2001) Zebrafish wnt8 encodes two wnt8 proteins on a bicistronic transcript and is required for mesoderm and neurectoderm patterning. Developmental Cell 1: 103-14.

93. Neave B, Holder N, Patient R (1997) A graded response to BMP-4 spatially coordinates patterning of the mesoderm and ectoderm in the zebrafish. Mechanisms of Development 62: 183-95.

94. Zhang H, Bradley A (1996) Mice deficient for BMP2 are nonviable and have defects in amnion/chorion and cardiac development. Development 122: 2977-86.

95. Ying Y, Zhao GQ (2001) Cooperation of endoderm-derived BMP2 and extraembryonic ectoderm-derived BMP4 in primordial germ cell generation in the mouse. Developmental biology 232: 484-92.

96. Madabhushi M, Lacy E (2011) Anterior visceral endoderm directs ventral morphogenesis and placement of head and heart via BMP2 expression. Developmental Cell 21: 907-19.

97. Winnier G, Blessing M, Labosky PA, Hogan BL (1995) Bone morphogenetic protein-4 is required for mesoderm formation and patterning in the mouse. Genes & Development 9: 2105-16.

98. Pourquie O, Fan CM, Coltey M, Hirsinger E, Watanabe Y, et al. (1996) Lateral and axial signals involved in avian somite patterning: a role for BMP4. Cell 84: 461-71.

99. Strumpf D, Mao CA, Yamanaka Y, Ralston A, Chawengsaksophak K, et al. (2005) Cdx2 is required for correct cell fate specification and differentiation of trophectoderm in the mouse blastocyst. Development 132: 2093-102.

100. van den Akker E, Forlani S, Chawengsaksophak K, de Graaff W, Beck F, et al. (2002) Cdx1 and Cdx2 have overlapping functions in anteroposterior patterning and posterior axis elongation. Development 129: 2181-93.

101. Grainger S, Lam J, Savory JG, Mears AJ, Rijli FM, et al. (2012) Cdx regulates Dll1 in multiple lineages. Developmental biology 361: 1-11.

102. Monaghan AP, Kioschis P, Wu W, Zuniga A, Bock D, et al. (1999) Dickkopf genes are co-ordinately expressed in mesodermal lineages. Mechanisms of Development 87: 45-56.

103. Hashimoto H, Itoh M, Yamanaka Y, Yamashita S, Shimizu T, et al. (2000) Zebrafish Dkk1 functions in forebrain specification and axial mesendoderm formation. Developmental biology 217: 138-52.

104. Mukhopadhyay M, Shtrom S, Rodriguez-Esteban C, Chen L, Tsukui T, et al. (2001) Dickkopf1 is required for embryonic head induction and limb morphogenesis in the mouse. Developmental Cell 1: 423-34.

105. Miura S, Singh AP, Mishina Y (2010) Bmpr1a is required for proper migration of the AVE through regulation of Dkk1 expression in the pre-streak mouse embryo. Developmental biology 341: 246-54.

106. Russ AP, Wattler S, Colledge WH, Aparicio SA, Carlton MB, et al. (2000) Eomesodermin is required for mouse trophoblast development and mesoderm formation. Nature 404: 95-9.

107. Crossley PH, Martin GR (1995) The mouse Fgf8 gene encodes a family of polypeptides and is expressed in regions that direct outgrowth and patterning in the developing embryo. Development 121: 439-51.

108. Ohuchi H, Yoshioka H, Tanaka A, Kawakami Y, Nohno T, et al. (1994) Involvement of androgen-induced growth factor (FGF-8) gene in mouse embryogenesis and morphogenesis. Biochemical and biophysical research communications 204: 882-8.

109. Zheng Z, de Iongh RU, Rathjen PD, Rathjen J (2010) A requirement for FGF signalling in the formation of primitive streak-like intermediates from primitive ectoderm in culture. PLoS One 5: e12555.

110. Miura N, Wanaka A, Tohyama M, Tanaka K (1993) MFH-1, a new member of the fork head domain family, is expressed in developing mesenchyme. FEBS Lett 326: 171-6.

111. Monaghan AP, Kaestner KH, Grau E, Schutz G (1993) Postimplantation expression patterns indicate a role for the mouse forkhead/HNF-3 alpha, beta and gamma genes in determination of the definitive endoderm, chordamesoderm and neuroectoderm. Development 119: 567-78.

112. Neave B, Rodaway A, Wilson SW, Patient R, Holder N (1995) Expression of zebrafish GATA 3 (gta3) during gastrulation and neurulation suggests a role in the specification of cell fate. Mechanisms of Development 51: 169-82.

113. Sheng G, Stern CD (1999) Gata2 and Gata3: novel markers for early embryonic polarity and for non-neural ectoderm in the chick embryo. Mechanisms of Development 87: 213-6.

114. Manaia A, Lemarchandel V, Klaine M, Max-Audit I, Romeo P, et al. (2000) Lmo2 and GATA-3 associated expression in intraembryonic hemogenic sites. Development 127: 643-53.

115. Kelley C, Blumberg H, Zon LI, Evans T (1993) GATA-4 is a novel transcription factor expressed in endocardium of the developing heart. Development 118: 817-27.

116. Soudais C, Bielinska M, Heikinheimo M, MacArthur CA, Narita N, et al. (1995) Targeted mutagenesis of the transcription factor GATA-4 gene in mouse embryonic stem cells disrupts visceral endoderm differentiation in vitro. Development 121: 3877-88.

117. Arceci RJ, King AA, Simon MC, Orkin SH, Wilson DB (1993) Mouse GATA-4: a retinoic acid-inducible GATA-binding transcription factor expressed in endodermally derived tissues and heart. Molecular and Cellular Biology 13: 2235-46.

118. Bossard P, Zaret KS (1998) GATA transcription factors as potentiators of gut endoderm differentiation. Development 125: 4909-17.

119. Chen C, Ware SM, Sato A, Houston-Hawkins DE, Habas R, et al. (2006) The Vg1-related protein Gdf3 acts in a Nodal signaling pathway in the pre-gastrulation mouse embryo. Development 133: 319-29.

120. Andersson O, Bertolino P, Ibanez CF (2007) Distinct and cooperative roles of mammalian Vg1 homologs GDF1 and GDF3 during early embryonic development. Developmental biology 311: 500-11.

121. Christian JL, Moon RT (1993) Interactions between Xwnt-8 and Spemann organizer signaling pathways generate dorsoventral pattern in the embryonic mesoderm of Xenopus. Genes & Development 7: 13-28.

122. Blum M, Gaunt SJ, Cho KW, Steinbeisser H, Blumberg B, et al. (1992) Gastrulation in the mouse: the role of the homeobox gene goosecoid. Cell 69: 1097-106.

123. Tadano T, Otani H, Taira M, Dawid IB (1993) Differential induction of regulatory genes during mesoderm formation in Xenopus laevis embryos. Developmental genetics 14: 204-11.

124. Lemaire P, Gurdon JB (1994) A role for cytoplasmic determinants in mesoderm patterning: cell-autonomous activation of the goosecoid and Xwnt-8 genes along the dorsoventral axis of early Xenopus embryos. Development 120: 1191-9.

125. Thisse C, Thisse B, Halpern ME, Postlethwait JH (1994) Goosecoid expression in neurectoderm and mesendoderm is disrupted in zebrafish cyclops gastrulas. Developmental biology 164: 420-9.

126. Cross JC, Flannery ML, Blanar MA, Steingrimsson E, Jenkins NA, et al. (1995) Hxt encodes a basic helix-loop-helix transcription factor that regulates trophoblast cell development. Development 121: 2513-23.

127. Firulli AB, McFadden DG, Lin Q, Srivastava D, Olson EN (1998) Heart and extra-embryonic mesodermal defects in mouse embryos lacking the bHLH transcription factor Hand1. Nature Genetics 18: 266-70.

128. Srivastava D, Cserjesi P, Olson EN (1995) A subclass of bHLH proteins required for cardiac morphogenesis. Science 270: 1995-9.

129. Barnes RM, Firulli BA, Conway SJ, Vincentz JW, Firulli AB (2010) Analysis of the Hand1 cell lineage reveals novel contributions to cardiovascular, neural crest, extra-embryonic, and lateral mesoderm derivatives. Developmental dynamics : an official publication of the American Association of Anatomists 239: 3086-97.

130. Tsuchihashi T, Maeda J, Shin CH, Ivey KN, Black BL, et al. (2011) Hand2 function in second heart field progenitors is essential for cardiogenesis. Developmental biology 351: 62-9.

131. Srivastava D, Thomas T, Lin Q, Kirby ML, Brown D, et al. (1997) Regulation of cardiac mesodermal and neural crest development by the bHLH transcription factor, dHAND. Nature Genetics 16: 154-60.

132. Reichenbach B, Delalande JM, Kolmogorova E, Prier A, Nguyen T, et al. (2008) Endoderm-derived Sonic hedgehog and mesoderm Hand2 expression are required for enteric nervous system development in zebrafish. Developmental biology 318: 52-64.

133. Vincent SD, Robertson EJ (2004) Targeted insertion of an IRES Cre into the Hnf4alpha locus: Cre-mediated recombination in the liver, kidney, and gut epithelium. Genesis 39: 206-11.

134. Weber H, Holewa B, Jones EA, Ryffel GU (1996) Mesoderm and endoderm differentiation in animal cap explants: identification of the HNF4-binding site as an activin A responsive element in the Xenopus HNF1alpha promoter. Development 122: 1975-84.

135. Kolm PJ, Sive HL (1995) Regulation of the Xenopus labial homeodomain genes, HoxA1 and HoxD1: activation by retinoids and peptide growth factors. Developmental biology 167: 34-49.

136. Thompson JR, Chen SW, Ho L, Langston AW, Gudas LJ (1998) An evolutionary conserved element is essential for somite and adjacent mesenchymal expression of the Hoxa1 gene. Developmental dynamics : an official publication of the American Association of Anatomists 211: 97-108.

137. Wendling O, Dennefeld C, Chambon P, Mark M (2000) Retinoid signaling is essential for patterning the endoderm of the third and fourth pharyngeal arches. Development 127: 1553-62.

138. Murphy P, Hill RE (1991) Expression of the mouse labial-like homeobox-containing genes, Hox 2.9 and Hox 1.6, during segmentation of the hindbrain. Development 111: 61-74.

139. Huang D, Chen SW, Langston AW, Gudas LJ (1998) A conserved retinoic acid responsive element in the murine Hoxb-1 gene is required for expression in the developing gut. Development 125: 3235-46.

140. Roche E, Sepulcre P, Reig JA, Santana A, Soria B (2005) Ectodermal commitment of insulin-producing cells derived from mouse embryonic stem cells. FASEB journal : official publication of the Federation of American Societies for Experimental Biology 19: 1341-3.

141. Cai CL, Liang X, Shi Y, Chu PH, Pfaff SL, et al. (2003) Isl1 identifies a cardiac progenitor population that proliferates prior to differentiation and contributes a majority of cells to the heart. Developmental Cell 5: 877-89.

142. Nathan E, Monovich A, Tirosh-Finkel L, Harrelson Z, Rousso T, et al. (2008) The contribution of Islet1-expressing splanchnic mesoderm cells to distinct branchiomeric muscles reveals significant heterogeneity in head muscle development. Development 135: 647-57.

143. Shalaby F, Rossant J, Yamaguchi TP, Gertsenstein M, Wu XF, et al. (1995) Failure of blood-island formation and vasculogenesis in Flk-1-deficient mice. Nature 376: 62-6.

144. Yang L, Soonpaa MH, Adler ED, Roepke TK, Kattman SJ, et al. (2008) Human cardiovascular progenitor cells develop from a KDR+ embryonic-stem-cell-derived population. Nature 453: 524-28.

145. Saga Y, Hata N, Kobayashi S, Magnuson T, Seldin MF, et al. (1996) MesP1: a novel basic helix-loop-helix protein expressed in the nascent mesodermal cells during mouse gastrulation. Development 122: 2769-78.

146. Saga Y, Hata N, Koseki H, Taketo MM (1997) Mesp2: a novel mouse gene expressed in the presegmented mesoderm and essential for segmentation initiation. Genes & Development 11: 1827-39.

147. Hart AH, Hartley L, Sourris K, Stadler ES, Li R, et al. (2002) Mixl1 is required for axial mesendoderm morphogenesis and patterning in the murine embryo. Development 129: 3597-608.

148. Ng ES, Azzola L, Sourris K, Robb L, Stanley EG, et al. (2005) The primitive streak gene Mixl1 is required for efficient haematopoiesis and BMP4-induced ventral mesoderm patterning in differentiating ES cells. Development 132: 873-84.

149. Acampora D, Avantaggiato V, Tuorto F, Barone P, Perera M, et al. (1999) Differential transcriptional control as the major molecular event in generating Otx1-/- and Otx2-/- divergent phenotypes. Development 126: 1417-26.

150. Simeone A, Acampora D, Mallamaci A, Stornaiuolo A, D'Apice MR, et al. (1993) A vertebrate gene related to orthodenticle contains a homeodomain of the bicoid class and

demarcates anterior neuroectoderm in the gastrulating mouse embryo. Embo J 12: 2735-47.

151. Schatteman GC, Morrison-Graham K, van Koppen A, Weston JA, Bowen-Pope DF (1992) Regulation and role of PDGF receptor alpha-subunit expression during embryogenesis. Development 115: 123-31.

152. Orr-Urtreger A, Lonai P (1992) Platelet-derived growth factor-A and its receptor are expressed in separate, but adjacent cell layers of the mouse embryo. Development 115: 1045-58.

153. Tada S, Era T, Furusawa C, Sakurai H, Nishikawa S, et al. (2005) Characterization of mesendoderm: a diverging point of the definitive endoderm and mesoderm in embryonic stem cell differentiation culture. Development 132: 4363-74.

154. Shinbrot E, Peters KG, Williams LT (1994) Expression of the platelet-derived growth factor beta receptor during organogenesis and tissue differentiation in the mouse embryo. Developmental dynamics : an official publication of the American Association of Anatomists 199: 169-75.

155. Ghil JS, Chung HM (1999) Evidence that platelet derived growth factor (PDGF) action is required for mesoderm patterning in early amphibian (Xenopus laevis) embryogenesis. The International Journal of Developmental Biology 43: 329-34.

156. Wilkinson DG, Bhatt S, Herrmann BG (1990) Expression pattern of the mouse T gene and its role in mesoderm formation. Nature 343: 657-9.

157. Rashbass P, Cooke LA, Herrmann BG, Beddington RS (1991) A cell autonomous function of Brachyury in T/T embryonic stem cell chimaeras. Nature 353: 348-51.

158. Smith JC, Price BM, Green JB, Weigel D, Herrmann BG (1991) Expression of a Xenopus homolog of Brachyury (T) is an immediate-early response to mesoderm induction. Cell 67: 79-87.

159. Kispert A, Herrmann BG (1994) Immunohistochemical analysis of the Brachyury protein in wild-type and mutant mouse embryos. Developmental biology 161: 179-93.

160. Kubo A, Shinozaki K, Shannon JM, Kouskoff V, Kennedy M, et al. (2004) Development of definitive endoderm from embryonic stem cells in culture. Development 131: 1651-62.

161. Inman KE, Downs KM (2006) Localization of Brachyury (T) in embryonic and extraembryonic tissues during mouse gastrulation. Gene Expr Patterns 6: 783-93.

162. Takada S, Stark KL, Shea MJ, Vassileva G, McMahon JA, et al. (1994) Wnt-3a regulates somite and tailbud formation in the mouse embryo. Genes & Development 8: 174-89.

163. Gomez E, Gutierrez-Adan A, Diez C, Bermejo-Alvarez P, Munoz M, et al. (2009) Biological differences between in vitro produced bovine embryos and parthenotes. Reproduction 137: 285-95.

164. Mann MR, Lee SS, Doherty AS, Verona RI, Nolen LD, et al. (2004) Selective loss of imprinting in the placenta following preimplantation development in culture. Development 131: 3727-35.

165. Bai H, Sakurai T, Someya Y, Konno T, Ideta A, et al. (2011) Regulation of trophoblast-specific factors by GATA2 and GATA3 in bovine trophoblast CT-1 cells. J Reprod Dev 57: 518-25.

166. Guillemot F, Caspary T, Tilghman SM, Copeland NG, Gilbert DJ, et al. (1995) Genomic imprinting of Mash2, a mouse gene required for trophoblast development. Nature Genetics 9: 235-42.

167. Holland MP, Bliss SP, Berghorn KA, Roberson MS (2004) A role for CCAAT/enhancer-binding protein beta in the basal regulation of the distal-less 3 gene promoter in placental cells. Endocrinology 145: 1096-105.

168. Marchand M, Horcajadas JA, Esteban FJ, McElroy SL, Fisher SJ, et al. (2011) Transcriptomic signature of trophoblast differentiation in a human embryonic stem cell model. Biology of Reproduction 84: 1258-71.

169. Rull K, Hallast P, Uuskula L, Jackson J, Punab M, et al. (2008) Fine-scale quantification of HCG beta gene transcription in human trophoblastic and non-malignant non-trophoblastic tissues. Mol Hum Reprod 14: 23-31.

170. Rull K, Laan M (2005) Expression of beta-subunit of HCG genes during normal and failed pregnancy. Human reproduction 20: 3360-8.

171. Uuskula L, Rull K, Nagirnaja L, Laan M (2011) Methylation allelic polymorphism (MAP) in chorionic gonadotropin beta5 (CGB5) and its association with pregnancy success. J Clin Endocrinol Metab 96: E199-207.

172. Ushizawa K, Takahashi T, Hosoe M, Ishiwata H, Kaneyama K, et al. (2007) Global gene expression analysis and regulation of the principal genes expressed in bovine placenta in relation to the transcription factor AP-2 family. Reprod Biol Endocrinol 5: 17.

173. Lytras A, Detillieux K, Cattini PA (2011) Identification of functional CCAAT/enhancer-binding protein and Ets protein binding sites in the human chorionic somatomammotropin enhancer sequences. J Mol Endocrinol 47: 179-93.

174. Yamada K, Ogawa H, Honda S, Harada N, Okazaki T (1999) A GCM motif protein is involved in placenta-specific expression of human aromatase gene. The Journal of biological chemistry 274: 32279-86.

175. Donnison M, Beaton A, Davey HW, Broadhurst R, L'Huillier P, et al. (2005) Loss of the extraembryonic ectoderm in Elf5 mutants leads to defects in embryonic patterning. Development 132: 2299-308.

176. Ng RK, Dean W, Dawson C, Lucifero D, Madeja Z, et al. (2008) Epigenetic restriction of embryonic cell lineage fate by methylation of Elf5. Nature Cell Biology 10: 1280-90.

177. Pearton DJ, Broadhurst R, Donnison M, Pfeffer PL (2011) Elf5 regulation in the trophectoderm. Developmental biology 360: 343-50.

178. Hemberger M, Udayashankar R, Tesar P, Moore H, Burton GJ (2010) ELF5-enforced transcriptional networks define an epigenetically regulated trophoblast stem cell compartment in the human placenta. Human Molecular Genetics 19: 2456-67.

179. Li Y, Lemaire P, Behringer RR (1997) Esx1, a novel X chromosome-linked homeobox gene expressed in mouse extraembryonic tissues and male germ cells. Developmental biology 188: 85-95.

180. Wen F, Tynan JA, Cecena G, Williams R, Munera J, et al. (2007) Ets2 is required for trophoblast stem cell self-renewal. Developmental biology 312: 284-99.

181. Georgiades P, Rossant J (2006) Ets2 is necessary in trophoblast for normal embryonic anteroposterior axis development. Development 133: 1059-68.

182. Yamamoto H, Flannery ML, Kupriyanov S, Pearce J, McKercher SR, et al. (1998) Defective trophoblast function in mice with a targeted mutation of Ets2. Genes & Development 12: 1315-26.

183. Basyuk E, Cross JC, Corbin J, Nakayama H, Hunter P, et al. (1999) Murine Gcm1 gene is expressed in a subset of placental trophoblast cells. Developmental dynamics : an official publication of the American Association of Anatomists 214: 303-11.

184. Anson-Cartwright L, Dawson K, Holmyard D, Fisher SJ, Lazzarini RA, et al. (2000) The glial cells missing-1 protein is essential for branching morphogenesis in the chorioallantoic placenta. Nature Genetics 25: 311-4.

185. Rachmilewitz J, Gileadi O, Eldar-Geva T, Schneider T, de-Groot N, et al. (1992) Transcription of the H19 gene in differentiating cytotrophoblasts from human placenta. Molecular Reproduction and Development 32: 196-202.

186. Potgens AJ, Gaus G, Frank HG, Kaufmann P (2001) Characterization of trophoblast cell isolations by a modified flow cytometry assay. Placenta 22: 251-5.

187. Jaquemar D, Kupriyanov S, Wankell M, Avis J, Benirschke K, et al. (2003) Keratin 8 protection of placental barrier function. The Journal of cell biology 161: 749-56.

188. Aronow BJ, Richardson BD, Handwerger S (2001) Microarray analysis of trophoblast differentiation: gene expression reprogramming in key gene function categories. Physiol Genomics 6: 105-16.

189. Gallego MJ, Porayette P, Kaltcheva MM, Bowen RL, Vadakkadath Meethal S, et al. (2010) The pregnancy hormones human chorionic gonadotropin and progesterone induce human embryonic stem cell proliferation and differentiation into neuroectodermal rosettes. Stem Cell Res Ther 1: 28.

190. Kojima K, Kanzaki H, Iwai M, Hatayama H, Fujimoto M, et al. (1995) Expression of leukaemia inhibitory factor (LIF) receptor in human placenta: a possible role for LIF in the growth and differentiation of trophoblasts. Human reproduction 10: 1907-11.

191. Fant M, Weisoly DL, Cocchia M, Huber R, Khan S, et al. (2002) PLAC1, a trophoblast-specific gene, is expressed throughout pregnancy in the human placenta and modulated by keratinocyte growth factor. Molecular Reproduction and Development 63: 430-6.

192. Massabbal E, Parveen S, Weisoly DL, Nelson DM, Smith SD, et al. (2005) PLAC1 expression increases during trophoblast differentiation: evidence for regulatory interactions with the fibroblast growth factor-7 (FGF-7) axis. Molecular Reproduction and Development 71: 299-304.

193. Zhou GQ, Baranov V, Zimmermann W, Grunert F, Erhard B, et al. (1997) Highly specific monoclonal antibody demonstrates that pregnancy-specific glycoprotein (PSG) is limited to syncytiotrophoblast in human early and term placenta. Placenta 18: 491-501.

194. Camolotto S, Racca A, Rena V, Nores R, Patrito LC, et al. (2010) Expression and transcriptional regulation of individual pregnancy-specific glycoprotein genes in differentiating trophoblast cells. Placenta 31: 312-9.

195. Teglund S, Zhou GQ, Hammarstrom S (1995) Characterization of cDNA encoding novel pregnancy-specific glycoprotein variants. Biochemical and biophysical research communications 211: 656-64.

196. Lei KJ, Gluzman Y, Pan CJ, Chou JY (1992) Immortalization of virus-free human placental cells that express tissue-specific functions. Mol Endocrinol 6: 703-12.

197. Kim J, Zhao K, Jiang P, Lu ZX, Wang J, et al. (2012) Transcriptome landscape of the human placenta. BMC Genomics 13: 115.

198. Khan WN, Teglund S, Bremer K, Hammarstrom S (1992) The pregnancy-specific glycoprotein family of the immunoglobulin superfamily: identification of new members and estimation of family size. Genomics 12: 780-7.

199. Huch G, Hohn HP, Denker HW (1998) Identification of differentially expressed genes in human trophoblast cells by differential-display RT-PCR. Placenta 19: 557-67.

200. Hughes M, Dobric N, Scott IC, Su L, Starovic M, et al. (2004) The Hand1, Stra13 and Gcm1 transcription factors override FGF signaling to promote terminal differentiation of trophoblast stem cells. Developmental biology 271: 26-37.

201. Yagi R, Kohn MJ, Karavanova I, Kaneko KJ, Vullhorst D, et al. (2007) Transcription factor TEAD4 specifies the trophectoderm lineage at the beginning of mammalian development. Development 134: 3827-36.

202. Nishioka N, Inoue K, Adachi K, Kiyonari H, Ota M, et al. (2009) The Hippo signaling pathway components Lats and Yap pattern Tead4 activity to distinguish mouse trophectoderm from inner cell mass. Developmental Cell 16: 398-410.

203. Ralston A, Cox BJ, Nishioka N, Sasaki H, Chea E, et al. (2010) Gata3 regulates trophoblast development downstream of Tead4 and in parallel to Cdx2. Development 137: 395-403.

204. Werling U, Schorle H (2002) Transcription factor gene AP-2 gamma essential for early murine development. Molecular and Cellular Biology 22: 3149-56.

205. Isermann B, Sood R, Pawlinski R, Zogg M, Kalloway S, et al. (2003) The thrombomodulin-protein C system is essential for the maintenance of pregnancy. Nature medicine 9: 331-7.

206. Sood R, Sholl L, Isermann B, Zogg M, Coughlin SR, et al. (2008) Maternal Par4 and platelets contribute to defective placenta formation in mouse embryos lacking thrombomodulin. Blood 112: 585-91.
