# Supplementary material for: Genome-Wide Analysis Reveals the Unique Stem Cell Identity of Human Amniocytes
Source: PLoS One. 2013 Jan 10;8(1):e53372. doi: 10.1371/journal.pone.0053372 (PMC3542377; doi:10.1371/journal.pone.0053372)
Supplement: Table S6 — Timeline and patient samples used in study, related to Figures 1 – 7 . Panel A is the experimental timeline that depicts time since amniocentesis, culture time at ARUP Labs (red bar) and culture time in our lab (green bar). Table S6B is a summary of total patient samples used in this study. (Patient samples were de-identified and new patient numbers were given in a general order they were received). (X marks) in table indicates type of analysis tested for each sample. Table S6C summarizes RNA-seq samples and read counts. Table S6D is a complete list of antibodies used in this study. Table S6E is a complete list of primers used in this study. (PDF) [file pone.0053372.s007.pdf]

**Table S6.** Timeline and patient samples used in study, related to Figures 1-7. Panel A is the experimental timeline that depicts time since amniocentesis, culture time at ARUP Labs (red bar) and culture time in our lab (green bar). **Table S6B** is a summary of total patient samples used in this study. (Patient samples were de-identified and new patient numbers were given in a general order they were received). (X marks) in table indicates type of analysis tested for each sample. **Table S6C** summarizes RNA-seq samples and read counts. **Table S6D** is a complete list of antibodies used in this study. **Table S6E** is a complete list of primers used in this study.

A

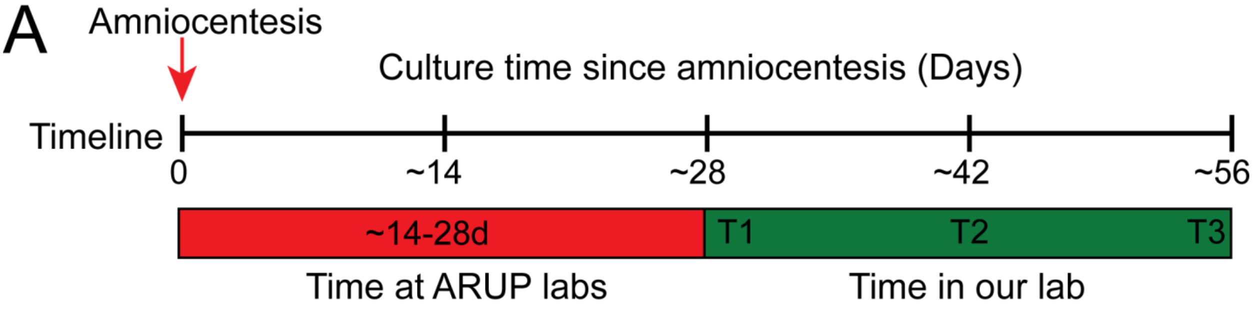


**Table S6B.** Summary of 60 patient samples used in this study.

| Patient | IHC | FACS | qPCR | RNA-seq | Clonal Analysis | G.A. | Gender | T1 | T2 | T3 |
|---|---|---|---|---|---|---|---|---|---|---|
| A7 | X | | | | | 15 | XX | | | |
| A8 | X | | | | | 34 | XX | | | |
| A10 | X | | | | | 16 | XX | | | |
| A11 | X | | | | | 16 | XX | | | |
| A19 | X | X | | | | 17 | XY | | | |
| A26 | X | | | | | 16 | XX | | | |
| A29 | X | | | | | 19 | XX | | | |
| A35 | X | | | | | 28 | XY | | | |
| A39 | X | | | | | 33 | XY | | | |
| A42 | X | | | | | 15 | XY | | | |
| A48 | X | | | | | 14 | XY | | | |
| A49 | X | | | | | 25 | XY | | | |
| A50 | X | | | | | 30 | XX | | | |
| A52 | X | | | | | 15 | XX | | | |
| A53 | X | | | | | 30 | XX | | | |
| A54 | X | | | | | 15 | XX | | | |
| A60 | | | | X | | 16 | XY | X | X | X |
| A63 | X | | X | X | | 15 | XY | X | X | X |
| A64 | | | | X | | 16 | XY | X | X | X |
| A65 | | | X | X | | 15 | XY | | | |
| A67 | | | X | X | | 16 | XX | X | X | X |
| A68 | | | X | X | | 32 | XY | | | |
| A70 | X | | | | | 17 | XX | X | X | |

| | | | | | | | | | | |
|---|---|---|---|---|---|---|---|---|---|---|
| A71 | X | | X | X | | 15 | XY | | | |
| A72 | X | | X | X | | 16 | XY | | | |
| A73 | | | | X | | 16 | XX | X | X | X |
| A74 | X | | | X | | 17 | XX | X | X | X |
| A77 | X | | X | X | | 25 | XX | X | X | X |
| A78 | X | | X | X | | 36 | XX | X | X | X |
| A79 | X | | | | | 16 | XY | | | |
| A81 | X | | X | X | | 30 | XY | X | X | X |
| A90 | X | | | | | 17 | XY | | | |
| A92 | X | | | | | 30 | XX | | | |
| A94 | X | | | | | 17 | XX | | | |
| A95 | | | X | X | | 25 | XX | X | X | X |
| A96 | | | | X | | 35 | XX | X | X | X |
| A97 | X | | | | | 14 | XY | | | |
| A98 | X | | | | | 17 | XY | | | |
| A99 | X | | | | | 18 | XX | | | |
| A101 | X | | | | | 17 | XY | | | |
| A102 | | | X | X | | 33 | XX | | | |
| A103 | | | | X | | 33 | XX | X | X | X |
| A104 | X | | | | | 16 | XX | | | |
| A105 | X | | | | | 16 | XY | | | |
| A106 | | | X | X | | 35 | XX | | | |
| A108 | X | | | | | 17 | XY | | | |
| A111 | X | | X | X | | 17 | XX | | | |
| A112 | X | | | | | 17 | XX | | | |
| A113 | X | | X | X | | 17 | XY | | | |
| A114 | X | | X | X | | 16 | XY | | | |
| A115 | X | | X | X | | 17 | XY | | | |
| A116 | X | | | | | 17 | XY | | | |
| A117 | X | | X | X | | 30 | XY | | | |
| A123 | X | | | | | 30 | XY | | | |
| A190 | X | | | | | 19 | XX | | | |
| A194 | X | | | | | 15 | XY | | | |
| A200 | X | | | | | 28 | XX | | | |
| A203 | X | | | | | 25 | XX | | | |
| A208 | X | | | | | 21 | XX | | | |
| FAF2 | X | | | | | 28 | XX | | | |
| A256 | X | | | | | 18 | XX | | | |
| A259 | X | | | | | 16 | XY | | | |
| A262 | X | | | | | 19 | XY | | | |
| A263 | X | | | | | 19 | XY | | | |
| A264 | X | | | | X | 16 | XY | | | |
| A265 | X | | | | | 17 | XY | | | |
| A267 | X | | | | | 20 | XY | | | |
| A270 | | | | | X | 22 | XY | | | |
| A272 | X | | | | X | 16 | XY | | | |
| A273 | X | | | | X | 17 | XX | | | |
| A274 | X | | | | X | 17 | XY | | | |
| A275 | | | | | X | 16 | XX | | | |

| | | | | | | | | | | |
|---|---|---|---|---|---|---|---|---|---|---|
| A276 | | | | | X | 17 | XX | | | |
| A277 | | | | | X | 26 | XY | | | |
| A279 | X | | | | | 18 | XY | | | |
| A280 | X | | | | | 18 | XY | | | |
| A283 | X | | | | | 12 | XY | | | |
| A285 | X | | | | | 17 | XX | | | |
| A292 | X | | | | | 21 | XY | | | |
| A293 | X | | | | | 37 | XY | | | |

**Table S6C.** Summary of RNA-seq samples and read counts.

| Sample type | Samples | Annot. reads | Bam reads | Fq reads |
|---|---|---|---|---|
| Amniocyte | 37 | 762,109,834 | 854,402,347 | 1,062,390,986 |
| Fibroblast (NFF) | 3 | 46,912,878 | 58,622,479 | 69,029,796 |
| ESC | 19 | 341,808,026 | 428,229,138 | 487,291,117 |
| IPSC | 12 | 208,763,583 | 266,818,498 | 299,695,459 |

**Table S6D.** Complete list of antibodies used in this study.

| **Antigen** | **Cat #** | **Antigen species** | **Host** | **Mono/ poly** | **Isotype** | **Conc. (Working Dilution)** | **Staining detected in amniocytes** |
|---|---|---|---|---|---|---|---|
| α-Actinin | Sigma (A7732) | Rabbit | M | M | IgG1 | 1.0mg/ml (1:300) | Yes |
| Cgb1 | Abcam (ab9376) | Human | Rb | P | IgG | N/A (1:300) | No |
| cKit | Millipore (MAB1163) | Human | M | M | IgG2a | 1mg/ml (1:200) | No |
| cKit | Biolegend (313201) | Human | M | M | IgG1 | 0.5mg/ml (1:200) | No |
| cKit | Cell Signaling (Ab81) | Human | M | M | IgG1 | N/A (1:200) | Yes |
| c-myc | DSHB (9E10) | Human | M | M | IgG1 | 50ug/ml (1:50) | No |
| α-Fetoprotei | R&D Systems | Human | M | M | IgG1 | 500ug/ml | Yes |

| | | | | | | | |
|---|---|---|---|---|---|---|---|
| n | (MAB1368) | | | | | (1:300) | |
| JMJD3 | Novus Biologicals (NBP1-06640) | Mouse | Rb | P | IgG | 1.75mg/ml (1:500) | No |
| Klf4 | Millipore (09-821) | Synthetic mouse | Rb | P | IgG | N/A (1:300) | Yes |
| Lin28 | Cell Signaling (3695) | human | Rb | P | IgG | N/A (1:200) | No |
| Nanog | Abcam (ab21624) | Human | Rb | P | IgG | 0.2mg/ml (1:200) | No |
| Nanog | Cell signaling (4903) | Human | Rb | P | IgG | N/A (1:200) | No |
| Nanog | Millipore (AB5731) | Mouse | Rb | P | IgG | 1.9mg/ml (1:200) | Yes |
| Oct4 | Abcam (19857) | Human | Rb | P | IgG | 0.6mg/ml (1:200) | Yes |
| Oct4 | Cell Signaling (2750) | Human | Rb | P | IgG | N/A (1:200) | No |
| Oct4 | Santa Cruz (sc-9081) | Human | Rb | P | IgG | 200ug/ml (1:200) | Yes |
| PCNA | Abcam (Ab29) | Rat | M | M | IgG2a | 1mg/ml (1:300) | Yes |
| PH3 | Santa Cruz (sc-8656-R) | Human | Rb | P | IgG | 200ug/ml (1:300) | Yes |
| Sox2 | Cell Signaling (5024) | Human | Rb | P | IgG | N/A (1:200) | Yes |
| Sox2 | Cell Signaling (3509) | Human | Rb | P | IgG | N/A (1:200) | No |
| Sox2 | BioLegend (630802) | Human | Rb | P | IgG | 0.25mg/ml (1:200) | Yes |

| | | | | | | | |
|---|---|---|---|---|---|---|---|
| Sox17 | R&D Systems (AF1924) | Human | Goat | P | IgG | 0.2mg/ml (1:300) | Yes |
| SM22α | Abcam (ab14106) | Mouse | Rb | P | IgG | N/A (1:800) | Yes |
| SSEA1 | DSHB (MC-480) | Mouse | M | M | IgM | 28ug/ml (1:50) | Yes |
| SSEA3 | DSHB (MC-631) | Rat | M | M | IgM | 40ug/ml (1:50) | Yes |
| SSEA4 | DSHB (MC-813-70) | Mouse | M | M | IgG3 | 52ug/ml (1:50) | Yes |
| Tra-1-60 | Millipore (MAB4360) | Human | M | M | IgM | 1mg/ml (1:300) | Yes |
| Tra-1-60 | Cell Signaling (4746) | Human | M | M | IgM | N/A (1:300) | No |
| Tra-1-81 | Millipore (MAB4381) | Human | M | M | IgM | 1mg/ml (1:300) | Yes |
| Tra-1-81 | Abcam (ab16289) | Human | M | M | IgM | 1mg/ml (1:300) | No |
| Tra-2-49 | DSHB (6E) | Mouse | M | M | IgG1 | 36ug/ml (1:50) | No |
| Tra-2-54 | DSHB (2J) | Mouse | M | M | IgG1 | 16ug/ml (1:50) | No |
| Tubb3 | Abcam (ab78078) | Rat | M | M | IgG2a | 1mg/ml (1:300) | Yes |
| TSA-1 | Santa Cruz (sc-23087) | Human | G | P | IgG | 200 μg/ml (1:200) | Questionable |
| Wdr5 | Abcam | Human | M | M | IgG2b | 0.50mg/ml (1:200) | Yes |

**Table S6E.** Genes, accession numbers, and primer sets used in this study.

| Gene | Ensgene_id | Refseq_id | Marker | Forward Seq (5'-3') |
|---|---|---|---|---|

| | | | | **Reverse Seq (5’-3’)** |
|---|---|---|---|---|
| **Oct4 (Pou5f1)** | ENSG00000204531 | NM_001173531 | Puripotency/iPSC factor | caatttgccaagctcctga |
| | | | | cagatggtcttttggctgaac |
| **Sox2** | ENSG00000181449 | NM_003106 | Pluripotency/iPSC factor | ctccgggacatgatcagc |
| | | | | ggtagtgctgggacatgtgaa |
| **Nanog** | ENSG00000111704 | NM_024865 | Pluripotency/iPSC factor | tctccaacatcctgaacctca |
| | | | | ttgctattcttcggccagtt |
| **Klf4** | ENSG00000136826 | NM_004235 | Transcriptional repressor/iPSC factor | gggagaagacactgcgtca |
| | | | | ggaagcactgggggaagt |
| **Wdr5** | ENSG00000196363 | NM_017588 | Pluripotency | agagtggctggcaagttcat |
| | | | | tgacgaccaggctacatcg |
| **Fut4** | ENSG00000196371 | NM_002033 | Pluripotency | tcttggtgttaccgggattc |
| | | | | aggttaccttcaaaacctgaactc |
| **Kit (ckit)** | ENSG00000157404 | NM_000222 | Stem cell marker | gcagatttcagagagcacca |
| | | | | gtcgtgcacaagcagagg |
| **Gapdh** | ENSG00000111640 | NM_002046 | Reference | ctctgctcctcctgttcgac |
| | | | | gcccaatacgaccaaatcc |
